# *Hetero*-Diels-Alder Reactions of In Situ-Generated Azoalkenes with Thioketones; Experimental and Theoretical Studies †

**DOI:** 10.3390/molecules26092544

**Published:** 2021-04-27

**Authors:** Grzegorz Mlostoń, Katarzyna Urbaniak, Malwina Sobiecka, Heinz Heimgartner, Ernst-Ulrich Würthwein, Reinhold Zimmer, Dieter Lentz, Hans-Ulrich Reissig

**Affiliations:** 1 Department of Organic and Applied Chemistry, Faculty of Chemistry, University of Lodz, 12 Tamka Street, 91-403 Lodz, Poland; katarzyna.urbaniak@chemia.uni.lodz.pl (K.U.); melkies@wp.pl (M.S.); 2 Department of Chemistry, University of Zurich, Winterthurerstrasse 190, CH-8057 Zurich, Switzerland; heinz.heimgartner@chem.uzh.ch; 3 Organisch-Chemisches Institut and Center for Multiscale Theory and Computation (CMTC), Westfälische Wilhelms-Universität Münster, Corrensstrasse 40, 48149 Münster, Germany; 4 Institut für Chemie und Biochemie, Freie Universität Berlin, Takustrasse 3, 14195 Berlin, Germany; rzimmer@chemie.fu-berlin.de (R.Z.); dieter.lentz@fu-berlin.de (D.L.)

**Keywords:** (4 + 2)-cycloadditions, *hetero*-Diels-Alder reactions, azoalkenes, thioketones, sulfur heterocycles, organic reaction mechanisms, DFT computations

## Abstract

The *hetero*-Diels-Alder reactions of in situ-generated azoalkenes with thioketones were shown to offer a straightforward method for an efficient and regioselective synthesis of scarcely known N-substituted 1,3,4-thiadiazine derivatives. The scope of the method was fairly broad, allowing the use of a series of aryl-, ferrocenyl-, and alkyl-substituted thioketones. However, in the case of N-tosyl-substituted cycloadducts derived from 1-thioxo-2,2,4,4-tetramethylcyclobutan-3-one and the structurally analogous 1,3-dithione, a more complicated pathway was observed. By elimination of toluene sulfinic acid, the initially formed cycloadducts afforded 2*H*-1,3,4-thiadiazines as final products. Advanced DFT calculations revealed that the observed high regioselectivity was due to kinetic reaction control and that the (4 + 2)-cycloadditions of sterically less unhindered thiones occurred via highly unsymmetric transition states with shorter C..S-distances (2.27–2.58 Å) and longer N..C-distances (3.02–3.57 Å). In the extreme case of the sterically very hindered 2,2,4,4-tetramethylcyclobutan-1,3-dione-derived thioketones, a zwitterionic intermediate with a fully formed C‒S bond was detected, which underwent ring closure to the 1,3,4-thiadiazine derivative in a second step. For the hypothetical formation of the regioisomeric 1,2,3-thiadiazine derivatives, the DFT calculations proposed more symmetric transition states with considerably higher energies.

## 1. Introduction

**Figure 1:**
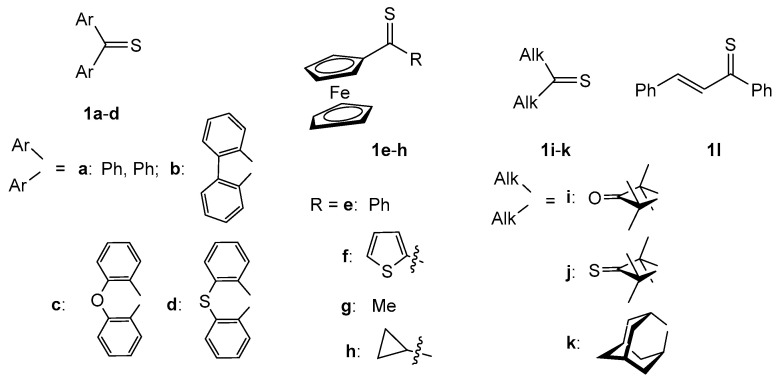
Synthetically useful representatives of aromatic **1a**–**d**; ferrocenyl **1e**–**h**; cycloaliphatic **1i**–**k**; and α,β-unsaturated **1l** thioketones.

In the most recent three decades, the poor reputation of thioketones (Figure 1) as smelly and unstable sulfur analogues of ketones changed dramatically and currently, many of them belonging to aromatic **1a**–**d**, ferrocenyl **1e**–**h**, or cycloaliphatic **1i**–**k** representatives are considered to be superior reagents for cycloaddition chemistry and related applications (Figure 1) [1]. Recently, thiochalcones, considered α,β-unsaturated analogues of thioketones, e.g., Figure 1 (**1l**), were demonstrated to act as active C=S dieno- and dipolarophiles [2,3,4].

Based on the results of kinetic studies, R. Huisgen named them first ‘superdipolarophiles’ [5] and almost at the same time, his former PhD student J. Sauer coined the term ‘superdienophiles’ for thioketones [6]. In our very recent publications, diverse thioketones were demonstrated to act as reactive building blocks yielding five-membered *S*-heterocycles (or products of their secondary conversions) not only with typical 1,3-dipoles such as thiocarbonyl *S*-methanides [7,8], diazo alkanes [9,10,11], nitrile imines [12,13], nitrile oxides [14], etc., but also with donor-acceptor cyclopropanes in the presence of an activating Lewis acid [15]. Moreover, trienamine-mediated asymmetric *hetero*-Diels-Alder reactions of thioketones leading to optically active 4*H*-thiopyran derivatives were described [16]. In numerous cases, mechanistic studies demonstrated that both (3 + 2)- as well as (4 + 2)-cycloadditions with aromatic and ferrocenyl thioketones do not follow the standard concerted pathways [17,18] but they occur via step-wise mechanisms involving diradicaloid or zwitterionic intermediates [7,9,19,20,21].

**Scheme 1:**
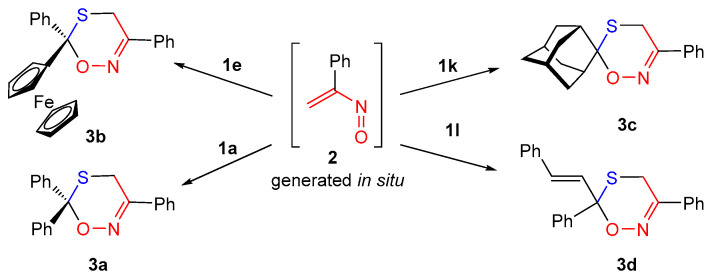
Regioselective (4 + 2)-cycloadditions of thioketones (**1a**, **1e**, **1k**) and thiochalcone (**1l**) with in situ-generated α-nitrosoethylene (**2**) leading to 1,5,2-oxathiazines (**3a**–**d**) [3,22].

In a very recent study from our laboratory, some thioketones, such as **1a**,**1e**,**1k** (Scheme 1) as well as thiochalcone **1l** (Scheme 1), were shown to behave as reactive hetero-dienophiles towards the in situ-generated α-nitrosostyrene **2** (Scheme 1), and the desired 1,5,2-oxathiazines Scheme 1 (**3a**–**d**) were obtained under mild conditions in a regioselective manner, in good to high yields (Scheme 1) [3,22].

**Scheme 2:**
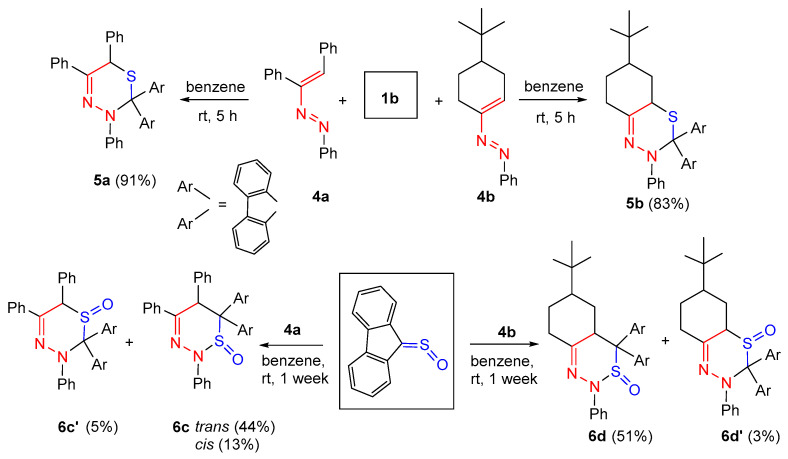
Comparison of the course of *hetero*-Diels-Alder reactions of thiofluorenone (**1b**) and the corresponding *S*-oxide (sulfine) with stable, isolable azoalkenes (**4a**,**b**) [32].

Azoalkenes (1,2-diaza-1,3-dienes) **4** (Scheme 2) are structurally and electronically related to 1-oxa-2-aza-1,3-dienes **2** (Scheme 2) and currently attract great attention as versatile substrates for *hetero*-Diels-Alder reactions performed with various dienophiles. The importance of this research is reflected by a large number of excellent review articles, which appeared in the last two decades [23,24,25,26,27]. Numerous original publications appear regularly. In addition, very recent reports focused on asymmetric cycloaddition reactions with this type of hetero-dienes and a few of them can be cited as representative examples [28,29,30,31].

In spite of this fact, the (4 + 2)-cycloadditions of azoalkenes **4** (Scheme 2) with ‘superdienophilic’ thioketones (Scheme 1 (**1**)) are practically unknown. However, a single, brief report by B. Bonini et al. on the *hetero*-Diels-Alder reaction of thiofluorenone (Scheme 2 (**1b**)) with some stable, isolable azoalkenes (Scheme 2 (**4a**,**b**)) appeared in 1981. These reactions were performed at room temperature in benzene solution and regioselectively led to 3,6-dihydro-2*H*-1,3,4-thiadiazines (Scheme 2 (**5a**,**b**)) in high to fair yields (Scheme 2) [32]. Notably, thiofluorenone *S*-oxide (sulfine), derived from the studied aromatic thioketone, reacted with Scheme 2 (**4a**) and Scheme 2 (**4b**) to give the corresponding *S*-oxides Scheme 2 (**6c**) and Scheme 2 (**6d**), respectively, derived from isomeric 1,2,3-thiadiazines as major products, however (Scheme 2, below). Thus, the (4 + 2)-cycloaddition onto the C=S(O) bond of these dienophiles occurred with inversed regioselectivity. Two other aromatic thioketones derived from thiobenzophenone (Scheme 1 (**1a**)), bearing two 4-MeC_6_H_4_ and 4-O_2_NC_6_H_4_ groups, respectively, failed to react with the tested azoalkenes (Scheme 2 (**4a**,**b**)). Therefore, the synthetic utility of this reported method seems to be very limited.

**Scheme 3:**
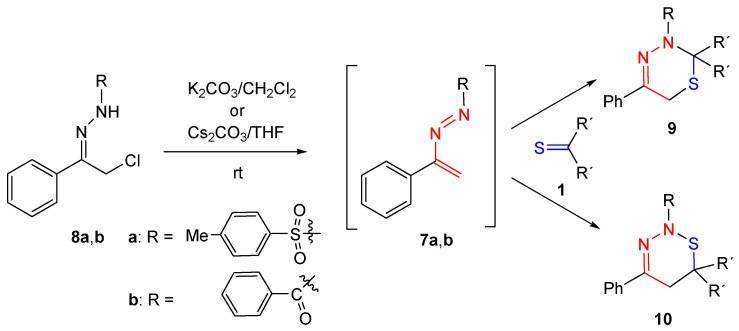
Generation of the reactive azoalkenes (**7a**,**7b**) from hydrazones (**8a**,**8b**), respectively, and their (4 + 2)-cycloadditions with thioketones **1** leading competitively either to 1,3,4-thiadiazines (**9**) or the regioisomeric 1,2,3-thiadiazines (**10**).

The goal of the present study was the examination of *hetero*-Diels-Alder reactions of in situ-generated, highly reactive azoalkenes (Scheme 3 (**7a**,**b**)) with a larger series of aromatic, ferrocenyl, and cycloaliphatic thioketones (Scheme 1 (**1**)). The regioselectivity of these cycloadditions was of particular interest since either the scarcely known 1,3,4-thiadiazines (Scheme 3 (**9**)) or the similarly rare 1,2,3-thiadiazines (Scheme 3 (**10**)) can be formed (Scheme 3). A careful study of typical examples of these (4 + 2)-cycloadditions by appropriate computational methods should help to understand the involved reaction mechanism(s) and the observed regioselectivity.

## 2. Results and Discussion

### 2.1. Experimental Work

The reactive azoalkenes (Scheme 3 (**7a**,**7b**)) were generated in situ from hydrazones (Scheme 3 (**8a,8b**)) derived from α-chloroacetophenone and *p*-toluenesulfonyl hydrazide or benzoylhydrazine, respectively, following a well-known and widely applied protocol [33]. In both cases, elimination of hydrogen chloride, leading to the required azoalkene, occurs smoothly at room temperature in the presence of potassium carbonate in CH_2_Cl_2_ solution (Scheme 3).

**Scheme 4:**
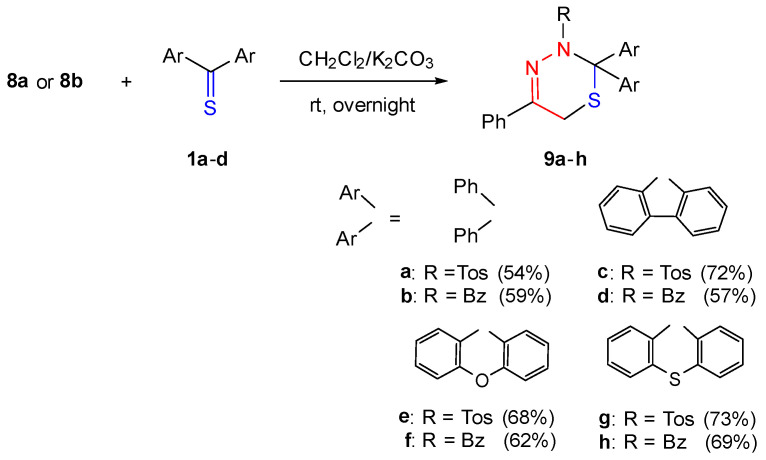
*Hetero*-Diels-Alder reactions of aromatic thioketones **1a**–**d** with azoalkenes **7a** and **7b** regioselectively leading to 3,6-dihydro-2*H*-1,3,4-thiadiazine derivatives **9a**–**h**.

As a typical reaction with thiobenzophenone (**1a**) and hydrazone (**8a**), the solution was stirred in the presence of potassium carbonate overnight, and after that time, the characteristic blue color of **1a** disappeared. After filtration and evaporation of the solvent, the ^1^H-NMR spectrum of the crude mixture revealed the presence of a characteristic singlet located at 3.21 ppm, which was attributed to the CH_2_ group present in cycloadduct (**9a**), formed as the sole product. After chromatographic purification, colorless crystals were isolated and identified as compound **9a** (Scheme 4, 54% yield). The same reaction carried out in THF using Cs_2_CO_3_ as a base for deprotonation of (**8a**) led to the same product in 45% yield, only. However, comparison of both procedures in two parallel performed experiments showed that the reaction carried out in CH_2_Cl_2_ was more efficient (shorter reaction time, better yield) and for that reason, further experiments were performed in CH_2_Cl_2_ using K_2_CO_3_ as an inexpensive and easily available base. The structure of **9a** was confirmed by further spectroscopic data and the most important indication was offered by signals in the ^13^C-NMR spectrum at 143.7 ppm, which was assigned to the C atom of the C=N group, and at 24.4 ppm attributed to the 6-CH_2_ unit. In addition, a less intense but characteristic signal of the C-2 atom was found at 77.7 ppm. The elemental analysis confirmed the molecular formula of **9a** as C_28_H_24_N_2_O_2_S_2_. Interestingly, while heating in a capillary, the colorless **9a** turned blue, indicating a retro-cycloaddition reaction leading to the extrusion of thiobenzophenone (**1a**).

An analogous course of the reaction was observed when hydrazone **8b** served as the precursor of azoalkene **7b**, which was trapped in situ by (**1a**), and cycloadduct (**9b**) was obtained as the sole product. After chromatographic purification, the yield of the latter compound was determined to be 59%. There was no evidence that regioisomeric 1,2,3-thiadiazine derivatives **10a** or **10b** were formed in these experiments.

**Figure 2:**
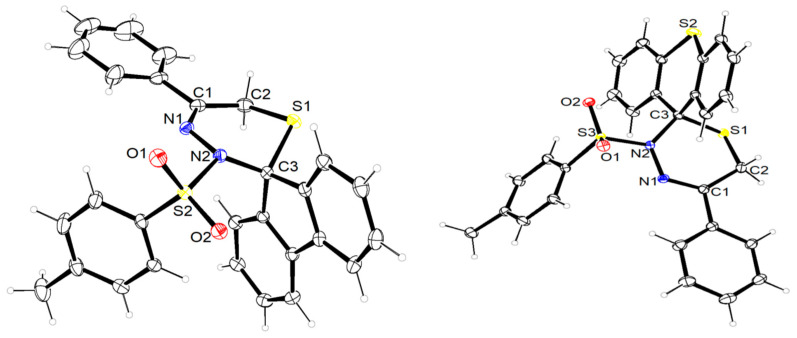
ORTEP Plots [34] of the molecular structures of compounds **9c** (**left**) and **9g** (**right**). Displacement ellipsoids are drawn at the 50% probability level.

In analogy to **1a**, the (4 + 2)-cycloadducts **9c**−**h** were obtained as crystalline products from aromatic thioketones **1b**–**d** using **8a** or **8b** as precursors of the in situ-generated azoalkenes **7a** and **7b**, respectively. Finally, the molecular structures of **9c** and **9g** were unambiguously confirmed by X-ray analyses (Figure 2).

Quite unexpectedly, two other aromatic thioketones, namely 2,3,6,7-dibenzocycloheptene-1-thione (**1m**) and its 10,11-dihydro analogue (**1n**), which have been applied in our earlier studies focused on *hetero*-Diels-Alder reactions [35], failed to react with **7a** even after 3 days at rt and no discoloration of the blue solutions was observed after that time.

**Scheme 5:**
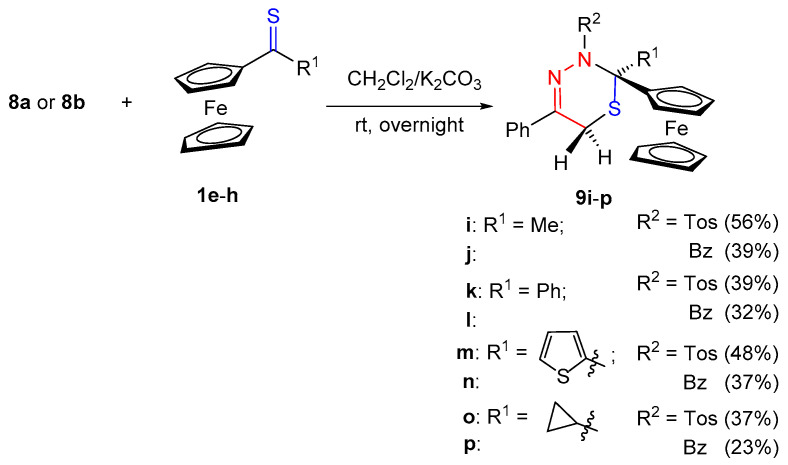
*Hetero*-Diels-Alder reactions of ferrocenyl-substituted thioketones **1e**–**h** with azoalkenes (**7a**) and (**7b**) leading to 2-ferrocenyl-substituted 3,6-dihydro-2*H*-1,3,4-thiadiazines (**9i**–**p**).

Ferrocenyl-substituted thioketones **1e**–**h** reacted similarly to aromatic representatives, and the reactions carried out at rt in CH_2_Cl_2_ with **8a**, applied as a precursor of the reactive azoalkene **7a**, led to the ferrocenyl substituted 1,3,4-thiadiazine derivatives (**9i**,**k**,**m**,**o**) in 39–56% yield (Scheme 5). A similar course of the studied reactions, regioselectively leading to cycloadducts **9j**,**l**,**n**, and **9p**, was observed with the *N*-benzoyl functionalized azoalkene **7b**. In this series, the lower yields of isolated products **9** can be explained by the fact, that along with the desired (4 + 2)-cycloadducts, substantial amounts (40–45%) of the corresponding ferrocenyl ketones were formed as side products and isolated during chromatographic separation of the crude mixtures as more polar fractions. However, the detailed mechanism of their formation under the applied reaction conditions (simple hydrolysis or oxidation of the C=S group) is currently unknown. In accordance with the postulated structure of heterocycles **9i**–**p**, the 6-CH_2_ group (the H atoms present in this group are diastereotopic in products **9i**–**p** appeared in the ^1^H-NMR spectra in all cases as *AB*-system in the region of 3.08–3.67 ppm with a coupling constant of approximately ^2^*J*_H,H_ = 17 Hz.

In a recent publication from our group, a possible non-concerted pathway of *hetero*-Diels-Alder reactions of aryl/ferrocenyl thioketones with a trienamine was discussed [16]. In the light of this discussion, the reaction of azoalkene **7b** with the cyclopropyl ferrocenyl thioketone **1h**, which is used for the first time in the herein studied *hetero*-Diels-Alder reaction, deserves a brief comment. The cyclopropyl substituent is known to act as so called ‘radical clock’ [36] and, therefore, cyclopropyl-substituted dienophiles found application in mechanistic studies aimed at the detection of a postulated diradicaloid intermediate [37,38] in the course of a non-concerted cycloaddition reaction. Isolation of **9o** as the sole product of the studied reaction of **1h** with **8b** suggests that its formation results from the concerted *hetero*-Diels-Alder reaction. Notably, diferrocenyl thioketone [39], which gave the desired (4 + 2)-cycloadduct in the earlier reported reaction with α-nitrosostyrene in a good yield of 75% [22], failed to react with both azoalkenes **7a** and **7b**. It is very likely that steric hindrance was the reason for the negative results in both cases.

**Scheme 6:**
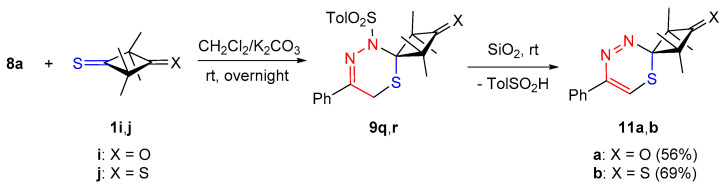
Elimination of sulfinic acid MeC_6_H_4_SO_2_H from initially formed cycloadducts of **9q**,**r**, leading to 2*H*-1,3,4-thiadiazine derivatives **11a**,**b**, respectively.

The reactions of the cycloaliphatic thioketones **1i**–**k** with azoalkenes **7a** and **7b** were performed analogously following the standard, overnight procedure. After a typical work-up, the ^1^H-NMR analysis of the crude reaction mixture obtained from **1i** and **7a** revealed the presence of two products with characteristic singlets localized at 3.50 and 6.54 ppm, respectively. Whereas one of them (the high-field shifted signal) might be attributed to the 6-CH_2_ group of (4 + 2)-cycloadduct **9p** (Scheme 5), the second, low-field shifted singlet suggested formation of a new product with a hitherto unknown structure. After column chromatography on silica gel, the product showing the high-field signal completely disappeared and the isolated material consisted exclusively of yellowish crystals with mp 93–95 °C. Notably, after its isolation, a substantial reduction of the mass was observed. Further spectroscopic data were required to solve the structure of this compound. In the ^1^H-NMR spectrum, neither signals characteristic for the tosyl moiety could be found nor those for the 6-CH_2_ group of 3,6-dihydro-2*H*-1,3,4-thiadiazine derivatives of type 9. Instead, a new singlet at 6.54 ppm suggested the presence of an olefinic fragment. On the other hand, the ^13^C-NMR spectrum showed, along with other absorptions characteristic for the Ph ring at 125.0 (2CH), 128.8 (2CH), 129.1 (1CH), and 134.7 (C_ar_) ppm, two signals localized at 107.4 and 151.8 ppm. In accordance with the ^1^H-NMR data, they were attributed to two C atoms of the alkene fragment –S–CH=C(Ph)–N=. In addition, the signal of the carbonyl group was found in the ^13^C-NMR spectrum at 217.6 ppm. This information allowed to postulate the structure of the isolated product as 2*H*-1,3,4-thiadiazine derivative (**11a**) (Scheme 6), which was supported by combustion analysis confirming the molecular formula C_16_H_18_N_2_OS. We assume that the elimination was favorable in these two examples since it relieved steric compression exerted by the close proximity of the N-tosyl group and the four methyl groups. Compounds with a 2*H*-1,3,4-thiadiazine substructure are very rare and only derivatives anellated to arene rings are reported in literature. Compounds such as the orange-colored **11a** can be regarded as *cis*-configured azoalkenes, which is in conjugation with a thioether moiety. The UV/Vis spectrum of **11a** in dichloromethane shows a strong absorption at 239 nm (log ε = 4.1 with a shoulder at 283 nm) and a weaker absorption at 358 nm (log ε = 2.8 with at 430 nm).

The analogous structure **11b** was attributed to the product isolated after an attempted *hetero*-Diels-Alder reaction of **1j** with **7a** (molar ratio of **1j**:**7a** = 1:1) (Scheme 6). The presence of the C=S group was proven by the characteristic orange-red color of the product and an absorption in the ^13^C-NMR spectrum at 277.4 ppm. Unexpectedly, the same 1:1 product **11b** was obtained in another experiment aimed at the synthesis of the bis-cycloadduct, starting with a molar ratio of **1j**:**7a** = 1:2. Apparently, steric hindrance in the initially formed cycloadduct **9r** prevents an efficient interaction of azoalkene **7a** with the second, unconverted C=S group. In contrast to **7a**, the *N*-benzoyl substituted azoalkene **7b** reacted with both thioketones **1i** and **1j**, yielding complex mixtures of products, which undergo decomposition during attempted chromatographic separation. It is worth mentioning that the ^1^H-NMR spectra of the crude product mixtures revealed the presence of a product with a characteristic *AB*-system found at 3.77 and 4.14 ppm, respectively, with *J* = 12.4 Hz. These signals were accompanied by a less intense singlet located at 3.83 ppm. It is very likely that similar products were formed in both reactions, but all attempts aimed at their isolation, either by column chromatography or by treatment with organic solvents, were unsuccessful and in both experiments, only further decomposition was observed. Similarly, adamantanethione (**1k**) did not yield stable products neither with **7a** nor **7b**.

**Scheme 7:**
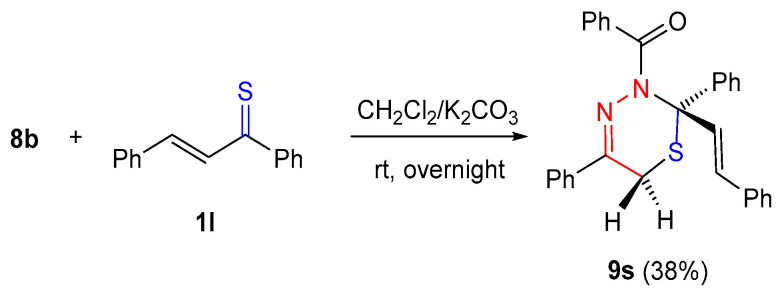
Regio- and periselective formation of the highly substituted cycloadduct **9s** from azoalkene **7b** and thiochalcone **1l**.

The experiments performed with thiochalcone **1l** and both azoalkenes **7** deserve a brief comment. Whereas the *N*-tosyl-substituted **7a** formed an unstable cycloadduct which could not be isolated in a pure form, the *N*-benzoyl analogue **7b** delivered the 1,3,4-thiadiazine derivative **9s** as a stable, crystalline product, isolated after chromatography in 38% yield (Scheme 7).

The (4 + 2)-cycloaddition also occurred regioselectively in this case leading to **9s** as the sole product. It was isolated as a stable crystalline material with no tendency to decomposition at ambient conditions. Its structure was easily elucidated based on spectroscopic data. For example, diagnostic signals of the 6-CH_2_ fragment appeared in the ^1^H-NMR spectrum as the expected *AB*-system localized at 3.50 and 3.62 ppm (^2^*J*_H,H_ = 17.0 Hz), and the styryl group appeared as two doublets found at 6.67 and 6.97 ppm (^3^*J*_H,H_ = 16.0 Hz). Furthermore, the C=N and C=O absorptions appeared in the ^13^C-NMR spectrum at 140.6 ppm and 170.2 ppm, respectively. As discussed in our publication about the *hetero*-Diels-Alder reactions of thiochalcones with α-nitrosostyrene [3], the combination of these two 4π systems can theoretically afford eight isomers, but it provided selectively one isomer. Apparently, azoalkene **7b** shows a similarly high regio- and periselectivity.

In summary of this experimental section, one can conclude that in most of the studied cases, the ‘superdienophilic’ thioketones **1** react with azoalkenes **7a**,**b** yielding a series of scarcely known 6*H*-1,3,4-thiadiazine derivatives of type **9** and **11**. In the latter case, the initially formed (4 + 2)-cycloadducts **9q**,**r** with the *N*-tosyl group located at N-3 underwent elimination of sulfinic acid and converted into the isolated 2*H*-1,3,4-thiadiazine derivatives **11**. Remarkably, the stability of the cycloadducts obtained with thiochalcone **1l** depends on the substitution pattern, and only in the case of N-benzoyl azoethylene **7b** could a stable product **9s** be isolated. All successful (4 + 2)-cycloadditions proceeded with high regioselectivity and the conceivable formation of 1,2,3-thiadiazine derivatives **10** was experimentally not observed.

### 2.2. Mechanistic Investigations by DFT Calculations

**Scheme 8:**
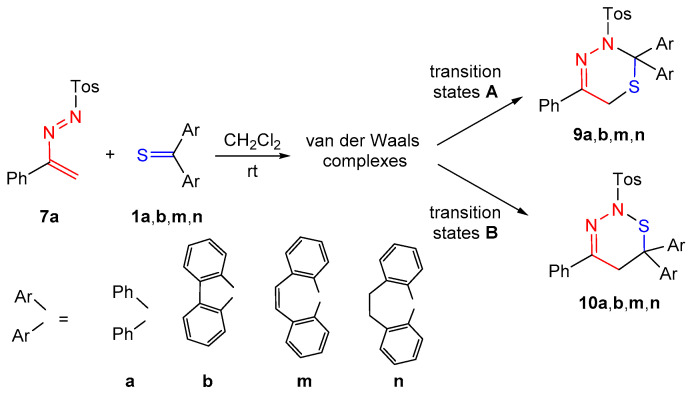
Cycloadditions of azoalkene (**7a**) with aryl-substituted thioketones (**1**) and pathways for both possible regioisomers (**9**) and (**10**).

The central question within this project concerns the experimentally observed explicit regioselectivity in favor of 1,3,4-thiadiazines derivatives **9** over the possible formation of 1,2,3-thiadiazines **10** (see Scheme 8) with respect to possible mechanisms involved, e.g., a concerted Diels-Alder reaction, a stepwise cycloaddition via zwitterions, or alternatives. In the following, the observed experimental outcome will be discussed on the basis of the Gibbs free energy surface as investigated by a comprehensive quantum chemical DFT study. Thus, mechanistic details, e.g., intermediates and reaction barriers via transition states, and the underlying thermodynamic and kinetic data will be in the center of investigation.

**Figure 3:**
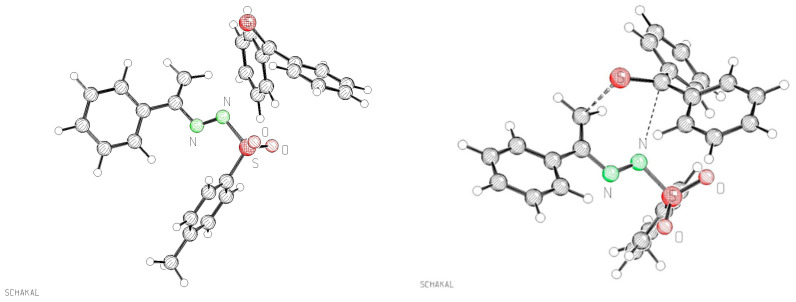
Van der Waals complex of azoalkene **7a** and thiobenzophenone (**1a**) (**left** side) and its transition state **A** (**right** side) leading to 1,3,4-thiadiazine derivative **9a**.

Starting with B3LYP/6-31G(d) [40,41] + GD3BJ [42,43] geometry optimizations for the gas phase, PBE1PBE/def2tzvp [44,45,46,47,48] + GD3BJ optimizations for the PCM solvent sphere of dichloromethane [49] were performed. In accordance with the reaction conditions (room temperature, no irradiation), only closed shell calculations were done, although diradical intermediates cannot principally be excluded. In the following, we discuss differences in Gibbs free energies (ΔG_298_) (kcal/mol) (see also Supplementary Materials for details); all relative energies refer to the sum of the starting materials. The structure and conformation of the observed 1,3,4-thiadiazines **9** for the calculations was derived from the X-ray structure of **9c** (Figure 2, left side). Furthermore, minima for van der Waals complexes formed from the two starting compounds were localized on the Gibbs free energy surface (see Supplementary Materials). They indicate substantial noncovalent interactions (van der Waals forces) between both reaction partners due to π-interactions of the aromatic moieties and London dispersion interactions of the chemical groups involved (for an example, see Figure 3). Their relative energy (3.0–4.3 kcal/mol) is low and only slightly dependent on the substitution pattern (Table 1, entries 1–6).

**Scheme 9:**
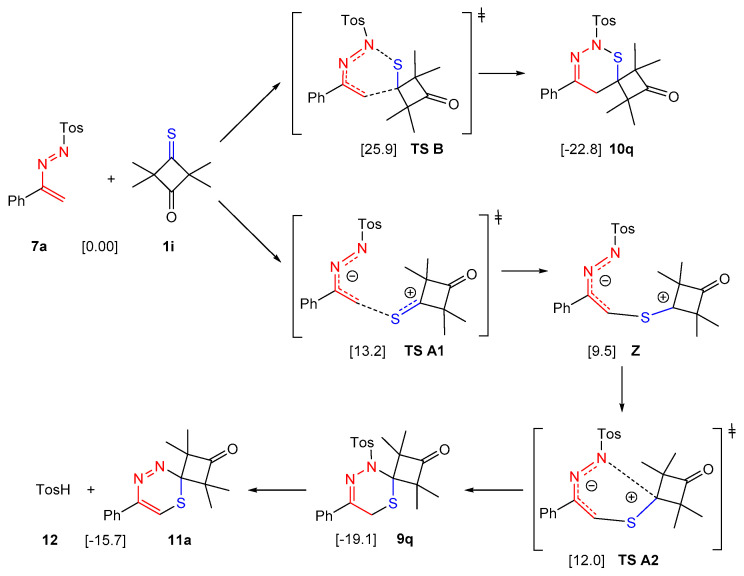
Hypothetical cycloaddition of azoalkene **7a** with sterically hindered thioketone **1i** to 1,2,3-thiadiazine derivative **10q** and step-wise reaction involving zwitterion **Z** leading to 1,3,4-thiadiazine derivative **9q** and elimination to 2*H*-1,3,4-thiadiazine **11a**. ((ΔG_298_) in kcal/mol).

Two principal classes of thioketones were in the center of interest for the reactions with azoalkene **7a**, namely **1a**, **1b**, **1m**, and **1n** (Scheme 8, Table 1, entries 1–4) as examples of aryl-substituted thioketones and 2,2,4,4-tetramethyl-3-thioxocyclobutan-1-one (**1i**) (Scheme 9, Table 1, entries 5–6) as a prototype of a sterically hindered and strained aliphatic thioketone. Table 1 summarizes the computational results obtained. It is interesting to see that the calculated Gibbs free reaction energies of all 1,3,4-thiadiazines (**9)** and 1,2,3-thiadiazines (**10**) obtained are very similar; thus, thermodynamic control of these reactions can be excluded. From the thermodynamic point of view, the formation of both isomeric products is likely, with small differences due to the substitution pattern of the respective thioketone.

However, the Gibbs free activation energies differed significantly in favor of the 1,3,4-thiadiazines **9**, indicating strong kinetic control with substantially lower activation barriers compared to the corresponding 1,2,3-thiadiazine regioisomers **10**. The barriers of (4 + 2)-cycloadditions of thioketones **1m** and **1n**, respectively leading to 1,3,4-thiadiazine **9m** and **9n**, were only slightly higher than those of **1a** and **1b**, but still not excessively high (compare entries 3 and 4 with entries 1 and 2). Hence, these calculations cannot easily explain the failed cycloadditions of **7a** with **1m** and **1n**.

**Figure 4:**
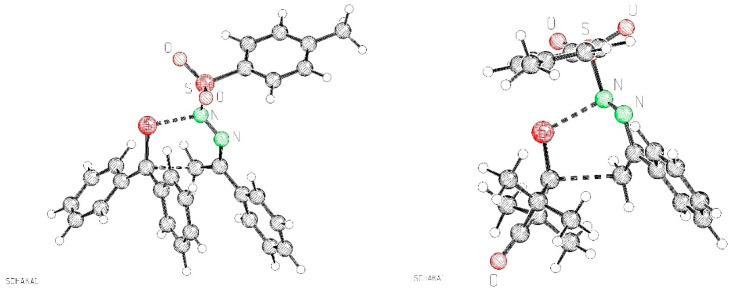
Transition states **B** of cycloadditions of azoalkene **7a** with thiobenzophenone (**1a**) (**left** side) or thiocyclobutanone derivative **1i** (**right** side) hypothetically leading to 1,2,3-thiadiazine derivative **10a** or **10iB,** respectively.

Concerning the reaction mechanisms of the cyclizations to **9** or **10**, substantial differences were found with regard to the atomic distances of the forming bonds in the respective transition states (Table 2). Thus, the transition states **A** for the formation of the 1,3,4-thiadiazines **9** were characterized by very unsymmetric structures with relatively small C..S-distances (2.27–2.58 Å) and quite large N..C-distances (3.02–3.57 Å) (differences from 0.62 to 1.30 Å). As an example, the transition state of the cycloaddition of azoalkene **7a** with thiobenzophenone (**1a**) is depicted in Figure 3 (right side), which illustrates the fairly unsymmetric formation of the new bonds. Computationally, these transition states are not easy to trace, since they are localized on a very flat energy surface (see below for an extreme example). In contrast, the hypothetical formation of the 1,2,3-thiadiazine isomers **10** is well in accord with a classical concerted *hetero*-Diels-Alder reaction with atomic distances of 2.15–2.55 Å and a maximal difference of 0.36 Å in transition states **B** (Figure 4).

**Figure 5:**
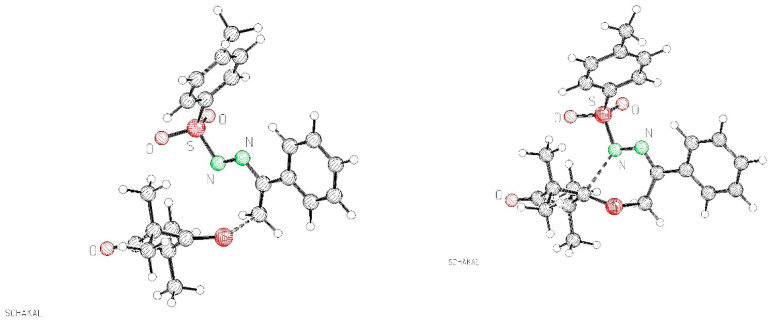
Transition states of the two-step cycloaddition of azoalkene **7a** with 3-thioxocyclobutanone derivative **1i**; left side: **TS A1** leading to zwitterion **Z** and **TS A2** for the cyclization of **Z** leading to 1,3,4-thiadiazine derivative **9q**.

A special example of the unsymmetric formation of the 1,3,4-thiadiazine **9** is the reaction of **7a** with the aliphatic, sterically hindered, ring-strained 2,2,4,4-tetramethyl-3-thioxocyclobutan-1-one (**1i**) involving the C=S bond, which was studied in more detail (see Scheme 9, Table 1 and Table 2, entry 5). The maximal difference of the distances between the reacting atoms is quite large (1.3 Å), indicating a two-step mechanism involving an open-chain zwitterionic intermediate instead of a concerted (4 + 2)-cycloaddition. Indeed, an intermediate **Z** and two transition states **TS A1** and **TS A2** could be localized on the energy surface (Figure 5). The energy of stabilized zwitterion **Z** lies only 9.5 kcal above that of the starting materials and its barrier for cyclization to **9q** is low. Apparently, it is the steric hindrance exhibited by the four methyl groups of **1i** that hampers the approach of the thiocarbonyl C-atom to the azoalkene N-atom and thus prevents the concerted (4 + 2)-cycloaddition in this case. These computational results imply further experimental studies in order to react zwitterionic intermediates such as **Z** with suitable trapping agents. The final elimination of the sulfinic acid **12** from **9q** affording **11a** was calculated to be slightly endergonic but supported by the experimental conditions (presence of base and of silica gel were not considered in the calculations).

As an alternative, the cycloadditions of **7a** to the C=O bond of **1i** were also considered. According to the computational results, such reaction modes are very unlikely due to quite high activation barriers and to low free reaction energies. Thus, not unexpectedly, the C=O bonds do not compete with the much more reactive C=S bonds for the formation of six-membered heterocyclic products (see Table 1 and Table 2, entries 6).

In summary, the computational investigations presented here interpret the experimental findings as the result of strong dominance of kinetic over thermodynamic control. The transition states leading to the observed 1,3,4-thiadiazine derivatives **9** obtained are quite asymmetric with relatively short C..S-distances but long N..C-distances. In extreme cases, when high steric hindrance comes into play as in **9q**, a two-step mechanism involving a zwitterionic intermediate **Z** dominates over the concerted (4 + 2)-cycloaddition reaction. In the other cases, competition of both pathways seems possible. Surprisingly, although the (4 + 2)-cycloaddition fits from the bond distances well to more “conventional” reaction pathways, this route to the 1,2,3-thiadiazines **10** is kinetically strongly disfavored. The proposed intermediacy of the zwitterionic intermediates discussed here should experimentally be verified by employing suitable trapping reagents.

## 3. Materials and Methods

### 3.1. Materials

The starting thioketones **1** were prepared by thionation of the corresponding ketones with Lawesson’s reagent (in the cases of **1a**, **1c**–**h**, and **1l**) [50], a mixed stream of H_2_S/HCl (for **1b**) [51], or by treatment with P_2_S_5_ in pyridine solution (for **1i**–**k**) [52]. The precursors of azoalkenes **7a** and **7b**, i.e., hydrazones **8a** [53] and **8b** [54], respectively, were synthesized from commercially available 2-chloroacetophenone (Chemat, Gdańsk, Poland) and the respective hydrazides (TolSO_2_NHNH_2_ or PhCONHNH_2_, respectively) in CH_2_Cl_2_ solution at rt (overnight stirring). After evaporation of the solvent, the residual oily materials were treated with a small portion of MeOH. The next day, the colorless crystals formed were filtered off and air-dried over a few hours.

### 3.2. Analytical Methods and Equipment

*General information*. All commercially available solvents and reagents were used as received. If not stated otherwise, reactions were performed in flame-dried flasks under an argon atmosphere and addition of the reactants by using a syringe; subsequent manipulations were conducted in the air. NMR spectra were taken with Bruker AVIII (^1^H-NMR (600 MHz); ^13^C-NMR (151 MHz); Bruker, Billerica, MA, USA). Chemical shifts are given relative to solvent residual peaks and integrals in accordance with assignments and coupling constants *J* in Hz. For detailed peak assignments, 2D spectra were measured (COSY, HMQC). The UV-Vis spectra were recorded with a JASCO V-630 spectrometer (JASCO, Easton, MD, USA) in CH_2_Cl_2_ solutions. The HRMS spectra were registered with a Varian 500-MS LC Ion Trap (Palo Alto, CA, USA) or with a Waters Synapt G2-Si mass spectrometer. The MS/ESI spectra were measured using a Varian 500 MS LS Ion Trap sp apparatus. IR measurements were performed with an Agilent Cary 630 FTIR spectrometer, in neat (Agilent, Santa Clara, CA, USA). Elemental analyses were obtained with a Vario EL III instrument. Melting points were determined in capillaries with an Aldrich Melt-Temp II apparatus and are uncorrected.

### 3.3. Quantum Chemical Calculations

Quantum chemical calculations (PBE1PBE/def2-TZVP+PCM (dichloromethane)+GD3BJ dispersion correction) [43,44,45,46,47,48,49,50] were performed on the basis of preceding B3LYP/6-31G(d) [41,42] +GD3BJ-geometry optimizations using the Gaussian 16, Revision B.01 [55], package of programs. To obtain the most reliable structural information, several conformers of each isomer were calculated, often after MM2-conformational analysis. The transition state localizations are based on reaction path calculations by stepwise, independent elongation of both relevant bonds beginning with the cycloadducts (“retro-Diels-Alder reaction”) and full optimization of all other parameters. Transition state searches or QST2 calculations on the basis of the calculated 3D-surfaces followed. In several cases, IRC-calculations were performed in order to characterize the transition states obtained. Minima for van der Waals complexes of the reacting starting material were also localized on the energy surface.

### 3.4. Synthesis

#### Reactions of Azoalkenes **7a**,**b** with Thioketones **1a**–**k** and Thiochalcone **1l**—General Procedures

The appropriate thioketone **1** (1 mmol) and 1.1 mmol of the corresponding precursor **8a** or **8b** were dissolved in 2 mL of freshly distilled dichloromethane and an excess of pre-dried potassium carbonate was added in small portions. The resulting suspension was stirred magnetically at room temperature under argon atmosphere until the color of the thioketone disappeared (overnight stirring is recommended). After completion of the reaction, the potassium carbonate was filtered off on a filter paper and the solvent was evaporated in a vacuum evaporator. The crude mixtures of products were purified by chromatography (SiO_2_) using a 1:4 mixture of petroleum ether and dichloromethane (PE/DCM) as an eluent. Analytically pure samples were obtained by crystallization of the isolated material from petroleum ether or hexane with a small admixture of dichloromethane.

*2,2,5*-*Triphenyl*-*3*-*(p*-*tolylsulfonyl)*-*6H*-*1,3,4*-*thiadiazine* (**9a**): Yield: 260 mg (54%). Colorless crystals (petroleum ether/CH_2_Cl_2_). M.p. 178–182 °C. ^1^H-NMR: δ 2.46 (s, CH_3_), 3.21 (s, CH_2_), 7.27, 7.73 (*AB* system, ^2^*J*_H,H_ = 8.1 Hz, 4CH_arom_), 7.35–7.44 (m, 9CH_arom_), 7.57–7.60 (m, 6CH_arom_) ppm. ^13^C-NMR: δ 21.7 (CH_3_), 24.4 (CH_2_), 77.7 (C_q_), 125.5, 128.0, 128.1, 128.5, 128.6, 129.0, 129.2, 129.4 (19CH_arom_), 137.3, 137.5, 139.4, 141.7, 143.7 (5C_arom_ and C=N) ppm. IR: ν 1595 (m), 1489 (m), 1355 (s), 1172 (s), 1090 (m), 1008 (s), 924 (m), 902 (m), 760 (s), 660 (s), 577 (m), 540 (vs) cm^−1^. C_28_H_24_N_2_O_2_S_2_ (484.63): calcd. C 69.39, H 4.99, N 5.78, S 13.23; found C 69.37, H 4.98, N 5.80, S 13.17.

*3-Benzoyl-2,2,5-triphenyl-6H-1,3,4-thiadiazine* (**9b**): Yield: 255 mg (59%). Colorless crystals (petroleum ether/CH_2_Cl_2_). M.p. 177–179 °C. ^1^H-NMR: δ 3.34 (s, CH_2_), 7.26–7.61 (m, 18CH_arom_), 7.86–7.88 (m, 2CH_arom_) ppm. ^13^C-NMR: δ 24.9 (CH_2_); 73.6 (C_q_), 125.2, 127.6, 128.2, 128.3, 128.4, 128.5, 129.0, 130.3, 131.1 (20CH_arom_), 135.6, 137.3, 139.2, 140.5 (4C_arom_ and C=N), 170.8 (C=O) ppm. IR: ν 1699 (C=O, s), 1605 (m), 1481 (m), 1311 (m), 1289 (s), 1155 (m), 1077 (m), 957 (m), 812 (m), 711 (s), 693 (vs) cm^−1^. MS/ESI: m/e 435 (100%, [M + 1]^+^). C_28_H_22_N_2_OS (434.56): calcd. C 77.39, H 5.10, N 6.45, S 7.38; found C 77.31, H 5.04, N 6.61, S 7.18.

*5*-*Phenyl*-*3*-*(p*-*tolylsulfonyl)spiro [6H*-*1,3,4*-*thiadiazine*-*2,9*′-*fluorene]* (**9c**): Yield: 345 mg (72%). Pale yellow crystals (petroleum ether/CH_2_Cl_2_). M.p. 179–182 °C. ^1^H-NMR: δ 2.33 (s, CH_3_), 3.95 (s, CH_2_), 7.00, 7.82 (*AB* system, ^2^*J*_H,H_ = 8.1 Hz, 4CH_arom_), 7.16–7.20 (m, 4CH_arom_), 7.28–7.30 (m, 2CH_arom_), 7.45–7.51 (m, 5CH_arom_), 7.88–7.89 (m, 2CH_arom_) ppm. ^13^C-NMR: δ 21.5 (CH_3_), 23.8 (CH_2_), 66.8 (C_q_), 120.8, 123.3, 125.5, 127.8, 128.7, 128.8, 129.3, 129.7 (17CH_arom_), 134.7, 137.0, 138.2, 141.9, 143.7, 145.2 (7C_arom_ and C=N) ppm. IR: ν 1587 (m), 1446 (m), 1353 (s), 1168 (s), 108 (m), 1002 (m), 775 (s), 743 (s), 667 (s), 577 (s), 548 (vs) cm^−1^. C_28_H_22_N_2_O_2_S_2_ (482.62): calcd. C 69.68, H 4.59, N 5.80, S 13.29; found C 69.42, H 4.56, N 5.93, S 13.23.

*3-Benzoyl-5*-*phenyl*-*spiro[6H*-*1,3,4*-*thiadiazine*-*2,9*′-*fluorene]* (**9d**): Yield: 245 mg (57%). Yellowish crystals (petroleum ether/CH_2_Cl_2_). M.p. 168 °C (dec.). ^1^H-NMR: δ 4.06 (s, CH_2_), 7.29–7.32 (m, 1CH_arom_), 7.37–7.52 (m, 11CH_arom_), 7.64–7.67 (m, 2CH_arom_), 7.76–7.79 (m, 2CH_arom_), 7.85–7.87 (m, 2CH_arom_) ppm. ^13^C-NMR: δ 23.3 (CH_2_); 65.6 (C_q_), 121.0, 121.6, 125.4, 127.5, 127.9, 128.7, 128.9, 129.4, 130.4, 131.0 (18CH_arom_), 134.8, 137.2, 138.2, 139.8, 146.3 (6C_arom_ and C=N), 168.8 (C=O) ppm. IR: ν 1714 (C=O, s), 1673 (m), 1599 (m), 1449 (m), 1285 (m), 1189 (m), 1095 (m), 917 (m), 807 (m), 730 (vs), 693 (s) cm^−1^. MS/ESI: m/e 433 (100%, [M + 1]^+^). C_28_H_20_N_2_OS (432.54): calcd. C 77.75, H 4.66, N 6.48, S 7.41; found C 77.48, H 4.68, N 6.74, S 7.16.

*5*-*Phenyl*-*3*-*(p*-*tolylsulfonyl)spiro[6H*-*1,3,4*-*thiadiazine*-*2,9*′-*xanthene]* (**9e**): Yield 340 mg (68%). Yellow–green crystals (petroleum ether/CH_2_Cl_2_). M.p. 145–147 °C. ^1^H-NMR: δ 2.42 (s, CH_3_), 3.66 (s, CH_2_), 7.03–7.05 (m, 2CH_arom_), 7.20–7.21 (m, 2CH_arom_), 7.24, 7.60 (*AB* system, ^2^*J*_H,H_ = 8.1 Hz, 4CHarom), 7.28–7.29 (m, 2CH_arom_), 7.31–7.39 (m, 2CH_arom_), 7.46–7.49 (m, 3CH_arom_), 7.79–7.81 (m, 2CH_arom_) ppm. ^13^C-NMR: δ 21.6 (CH_3_), 23.7 (CH_2_), 60.3 (C_q_), 117.0, 122.9, 125.6, 126.6, 128.7, 128.8, 129.3, 129.5, 129.8 (17CH_arom_), 134.9, 135.9, 136.9, 141.8, 144.2, 149.0 (7C_arom_ and C=N) ppm. IR: ν 1599 (m), 1476 (m), 1444 (s), 1302 (s), 1168 (s), 1086 (s), 1010 (m), 749 (vs), 579 (s), 546 (vs) cm^−1^. C_28_H_22_N_2_O_3_S_2_ (498.62): calcd. C 67.45, H 4.45, N 5.62, S 12.86; found C 67.28, H 4.46, N 5.66, S 12.84.

*3-Benzoyl-5*-*phenylspiro[6H*-*1,3,4*-*thiadiazine*-*2,9*′-*xanthene]* (**9f**): Yield: 280 mg (62%). Yellowish crystals (petroleum ether/CH_2_Cl_2_). M.p. 181 °C (dec). ^1^H-NMR: δ 3.67 (s, CH_2_), 7.06–7.08 (m, 2CH_arom_), 7.25–7.27 (m, 4CH_arom_), 7.32–7.36 (m, 2CH_arom_), 7.38–7.40 (m, 3CH_arom_), 7.53–7.54 (m, 1CH_arom_), 7.61–7.63 (m, 2CH_arom_), 7.85–7.87 (m, 2CH_arom_) ppm. ^13^C-NMR: δ = 23.1 (CH_2_), 58.7 (Cq), 117.3, 123.3, 124.2, 125.5, 127.6, 128.6, 123.3, 129.5, 130.2, 130.9 (18CH_arom_), 123.9, 135.1, 137.1, 141.0, 149.7 (6C_arom_ and C=N), 169.1 (C=O) ppm. IR: ν 1673 (s), 1599 (m), 1303 (m), 1474 (s), 1446 (s), 1311 (m), 1289 (s), 1159 (m), 1099 (m), 1017 (m), 887 (m), 797 (s), 749 (s), 712 (s), 689 (s) cm^−1^. MS/ESI: m/e 475 (100, [M]^+^). C_28_H_20_N_2_O_2_S∙1.5 H_2_O (475.56): calcd. C 70.72, H 4.87, N 5.89, S 6.74; found C 70.87, H 4.36, N 6.20, S 6.98.

*5*-*Phenyl*-*3*-*(p*-*tolylsulfonyl)spiro[6H*-*1,3,4*-*thiadiazine*-*2,9*′-*thioxanthene]* (**9g**): Yield: 375 mg (73%). Greenish crystals (petroleum ether/CH_2_Cl_2_). M.p. 161–163 °C. ^1^H-NMR: δ 2.46 (s, CH_3_), 3.36 (s, CH_2_), 7.17–7.19 (m, 2CH_arom_), 7.30–7.33 (m, 4CH_arom_), 7.40–7.42 (m, 4CH_arom_), 7.47–7.51 (m, 3CH_arom_), 7.80–7.82 (m, 4CH_arom_) ppm. ^13^C-NMR: δ 21.7 (CH_3_), 22.5 (CH_2_), 67.7 (C_q_), 125.6, 125.7, 126.0, 127.5, 128.5, 128.8, 128.9, 129.5, 129.8 (17CH_arom_), 128.4, 133.6, 136.1, 137.0, 142.4, 144.4 (7C_arom_ and C=N) ppm. IR: ν 1597 (m), 1444 (s), 1303 (m), 1168 (s), 1087 (s), 1008 (m), 771 (s), 742 (s), 652 (s), 557 (s), 546 (vs) cm^−1^. C_28_H_22_N_2_O_2_S_3_ (514.68): calcd. C 65.34, H 4.31, N 5.44, S 18.69; found C 65.25, H 4.36, N 5.57, S 18.69.

*3-Benzoyl*-*5*-*phenylspiro[6H*-*1,3,4*-*thiadiazine*-*2,9′*-*thioxanthene]* (**9h**): Yield: 320 mg (69%). Yellowish crystals (petroleum ether/CH_2_Cl_2_). M.p. 186 °C (dec). ^1^H-NMR: δ = 3.36 (s, CH_2_), 7.21–7.22 (m, 2CH_arom_), 7.27–7.32 (m, 5CH_arom_), 7.36–7.38 (m, 3CH_arom_), 7.46–7.48 (m, 2CH_arom_), 7.50–7.52 (m, 2CH_arom_), 7.57–7.59 (m, 2CH_arom_), 7.96–7.98 (m, 2CH_arom_) ppm. ^13^C-NMR: δ = 22.3 (CH_2_), 65.5 (C_q_), 125.1, 125.6, 126.3, 126.8, 127.7, 127.9, 128.6, 129.5, 130.3, 131.0 (18CH_arom_), 129.4, 133.5, 135.1, 137.0, 142.7 (6C_arom_ and C=N), 169.4 (C=O) ppm. IR: ν 1670 (C=O, s), 1602 (m), 1461 (m), 1386 (m), 1334 (m), 1285 (s), 1144 (m), 1073 (m), 913 (m), 793 (m), 745 (vs), 699 (s) cm^−1^. C_28_H_20_N_2_OS_2_∙1.5 H_2_O (491.60): calcd. C 68.41., H 4.72, N 5.70, S 13.04; found C 68.62, H 4.85, N 6.01, S 13.20.

*2*-*Ferrocenyl*-*2*-*methyl*-*5*-*phenyl*-*3*-*(p*-*tolylsulfonyl)*-*6H*-*1,3,4*-*thiadiazine* (**9i**)*:* Yield: 295 mg (56%). Beige crystals (petroleum ether/CH_2_Cl_2_). M.p. 124 °C (dec). ^1^H-NMR: δ 2.41, 2.50 (2s, 2CH_3_), 3.34, 3.67 (*AB* system, ^2^*J*_H,H_ = 16.6 Hz, CH_2_), 4.17–4.19 (m, 2CH_Fc_), 4.24–4.25 (m, 1CH_Fc_), 4.33 (s, 5CH_Fc_), 4.49–4.50 (m, 1CH_Fc_), 7.23, 7.70 (*AB* system, ^3^*J*_H,H_ = 8.2 Hz, 4CH_arom_), 7.37–7.39 (m, 3CH_arom_), 7.59–7.61 (m, 2CH_arom_) ppm. ^13^C-NMR: δ 21.6, 28.5 (2CH_3_), 24.3 (CH_2_), 66.7, 67.3, 68.6, 69.7, 69.8 (9*C*H_Fc_), 67.4 (C_q_), 90.6 (C_Fc_), 125.5, 128.4, 128.9, 129.2, 129.6 (9*C*H_arom_), 136.8, 137.2, 143.3, 144.2 (3C_arom_ and C=N) ppm. IR: ν 1597 (m), 1444 (m), 1357 (m), 1174 (s), 1120 (m), 1094 (m), 986 (m), 809 (s), 747 (m), 669 (s), 562 (s), 542 (vs) cm^−1^. MS-ESI: m/e 530 (100, [M]^+^), 531 (85, [M + 1]^+^), 553 (46, [M + Na]^+^). C_27_H_26_FeN_2_O_2_S_2_ (530.48): calcd. C 61.13, H 4.94, N 5.28, S 12.09; found C 60.94, H 4.97, N 5.43, S 11.99.

*3-Benzoyl-2*-*ferrocenyl*-*2*-*methyl*-*5-phenyl-6H*-*1,3,4*-*thiadiazine* (**9j**)*:* Yield: 185 mg (39%). Beige crystals (petroleum ether/CH_2_Cl_2_). M.p. 154 °C (dec). ^1^H-NMR: δ 2.45 (s, CH_3_), 3.45, 3.80 (*AB* system, ^2^*J*_H,H_ = 17.3 Hz, CH_2_), 4.19–4.20 (m, 1CH_Fc_), 4.23–4.25 (m, 1CH_Fc_), 4.35 (s, 5CH_Fc_), 4.37–4.39 (m, 2CH_Fc_), 7.27–7.29 (m, 4CH_arom_), 7.39–7.41 (m, 3CH_arom_), 7.44–7.47 (m, 1CH_arom_), 7.63–7.64 (m, 2CH_arom_) ppm. ^13^C-NMR: δ 24.0 (CH_2_), 26.1 (CH_3_), 62.6 (C_q_), 66.1 (1CH_Fc_), 66.5 (1CH_Fc_), 68.4 (1CH_Fc_), 68.7 (1CH_Fc_), 69.6 (5CH_Fc_), 91.7 (C_Fc_), 125.1, 127.4, 128.4, 128.8, 129.5, 130.2 (10CH_arom_), 136.9, 137.3, 139.1 (2C_arom_ and C=N), 170.9 (C=O) ppm. IR: ν 1689 (C=O, s), 1610 (m), 1481 (m), 1341 (m), 1337 (m), 1289 (s), 1174 (m), 1103 (m), 1002 (m), 902 (m), 752 (m), 748 (vs), 711 (s) cm^−1^. C_27_H_24_FeN_2_OS (480.41): calcd. C 67.50, H 5.04, N 5.83, S 6.67; found C 67.32, H 5.17, N 5.85, S 6.51.

*2*-*Ferrocenyl*-*2,5*-*diphenyl*-*3*-*(p*-*tolylsulfonyl)*-*6H*-*1,3,4*-*thiadiazine* (**9k**): Yield: 230 mg (39%). Beige crystals (petroleum ether/CH_2_Cl_2_). M.p. 158–160 °C. ^1^H-NMR: δ 2.44 (s, CH_3_), 3.08, 3.48 (*AB* system, ^2^*J*_HH_ = 16.9 Hz, CH_2_), 4.11 (s, 1CH_Fc_), 4.30 (d, *J* = 5.6 Hz, 2CH_Fc_), 4.39 (s, 6CH_Fc_), 7.21–8.20 (m, 14CH_arom_) ppm. ^13^C-NMR: δ 21.6 (CH_3_), 25.2 (CH_2_), 67.4, 68.0, 69.0, 70.2, 72.7 (9CH_Fc_), 74.5 (C_q_), 89.4 (C_Fc_), 125.5, 127.8, 128.1, 128.2, 128.4, 128.5, 128.8, 129.0 (14CH_arom_); 137.1, 137.6, 141.0, 141.5, 143.4 (4C_arom_ and C=N) ppm. IR: ν 1597 (m), 1446 (m), 1351 (m), 1304 (m), 1169 (s), 1088 (s), 1008 (m), 810 (m), 751 (m), 689 (s), 568 (s), 539 (vs) cm^−1^. MS-ESI: *m*/*z* 593 (100, [M + 1]^+^), 615 (27, [M + Na]^+^. C_32_H_28_FeN_2_O_2_S_2_ (592.55): calcd. C 64.86, H 4.76, N 4.73, S 10.82; found C 64.99, H 4.59, N 4.95, S 10.91.

*3-Benzoyl-2-ferrocenyl-2,5-diphenyl-6H-1,3,4*-*thiadiazine* (**9l**)*:* Yield: 175 mg (32%). Beige crystals (petroleum ether/CH_2_Cl_2_). M.p. 157 °C (dec). ^1^H-NMR: δ 3.17, 3.55 (*AB* system, ^2^*J*_HH_ = 17.1 Hz, CH_2_), 3.99–4.01 (m, 1CH_Fc_), 4.20–4.22 (m, 1CH_Fc_), 4.29–4.30 (m, 1CH_Fc_), 4.45 (s, 5CH_Fc_), 4.59–4.61 (m, 1CH_Fc_), 7.24–7.29 (m, 4CH_arom_), 7.35–7.44 (m, 7CH_arom_), 7.47–7.51 (m, 2CH_arom_), 7.70–7.74 (m, 2CH_arom_) ppm. ^13^C-NMR: δ 25.8 (CH_2_), 66.8 (C_q_), 67.0 (1CH_Fc_), 68.4 (1CH_Fc_), 69.6 (5CH_Fc_), 70.5 (1CH_Fc_), 71.3 (1CH_Fc_), 92.0 (C_Fc_), 125.2, 127.5, 127.9, 128.0, 128.2, 128.4, 128.9, 130.0, 130.7 (15CH_arom_), 136.2, 137.3, 139.1, 139.6 (3C_arom_, C=N), 170.5 (C=O) ppm. IR: ν 1677 (C=O, s), 1611 (m), 1490 (m), 1446 (m), 1394 (m), 1341 (m), 1282 (s), 1155 (m), 989 (m), 820 (m), 741 (s), 693 (vs) cm^−1^. MS/ESI: m/e 542 (100%, [M + 1]^+^). C_32_H_26_FeN_2_OS (542.48): calcd. C 70.85, H 4.83, N 5.16, S 5.91; found C 70.79, H 4.95, N 5.09, S 6.17.

*2*-*Ferrocenyl*-*5*-*phenyl*-*2*-*(2*-*thienyl)*-*3*-*(p*-*tolylsulfonyl)*-*6H*-*1,3,4*-*thiadiazine* (**9m**)*:* Yield: 290 mg (48%). Brown crystals (petroleum ether/CH_2_Cl_2_). M.p. 126–128 °C. ^1^H-NMR: δ 2.43 (s, CH_3_), 3.33, 3.58 (*AB* system, ^2^*J*_H,H_ = 16.9 Hz, CH_2_), 4.31–4.33 (m, 4CH_Fc_), 4.35–4.40 (m, 5CH_Fc_), 6.94 (pseudo t, *J* = 4.3 Hz, 1CH_arom_), 7.23–7.25 (m, 2CH_arom_), 7.38–7.42 (m, 5CH_arom_), 7.65–7.67 (m, 4CH_arom_) ppm. ^13^C-NMR: δ 21.5 (CH_3_), 25.3 (CH_2_), 67.4, 68.0, 68.7, 70.4, 73.1 (9CH_Fc_), 71.3 (C_q_), 89.2 (C_Fc_), 125.5, 125.7, 126.8, 128.4, 128.6, 128.8, 129.0, 129.1 (12CH_arom_), 137.0, 137.4, 141.5, 143.5, 145.3 (4C_arom_ and C=N) ppm. IR: ν 1599 (m), 1439 (m), 1353 (s), 1169 (s), 1090 (m), 1003 (m), 939 (m), 805 (s), 751 (s), 665 (s), 565 (s), 538 (vs) cm^−1^. C_30_H_26_FeN_2_O_2_S_3_ (598.58): calcd. C 60.30, H 4.38, N 4.68, S 16.07; found C 60.36, H 4.30, N 4.71, S 16.02.

*3-Benzoyl-2-ferrocenyl-5-phenyl-2-thienyl-6H-1,3,4-thiadiazine* (**9n**)*:* Yield: 205 mg (37%). Beige crystals (petroleum ether/CH_2_Cl_2_). M.p. 161 °C (dec). ^1^H-NMR: 3.48, 3.68 (*AB* system, ^2^*J*_H,H_ = 17.2 Hz, CH_2_), 4.19–4.20 (m, 1CH_Fc_), 4.19–4.20 (m, 1CH_Fc_), 4.22–4.23 (m, 1CH_Fc_), 4.29–4.30 (m, 1CH_Fc_), 4.46 (s, 5CH_Fc_), 4.57–4.60 (m, 1CH_Fc_), 6.90–6.92 (m, 1CH_arom_), 7.02–7.44 (m, 1CH_arom_), 7.25–7.31 (m, 4CH_arom_), 7.39–7.41 (m, 5CH_arom_), 7.46–7.47 (m, 1CH_arom_), 7.67–7.69 (m, 2CH_arom_) ppm. ^13^C-NMR: δ 26.1 (CH_2_), 67.1 (C_q_), 66.7 (1CH_Fc_), 67.3 (1CH_Fc_), 68.4 (C_Fc_), 70.1 (5CH_Fc_), 71.7 (1CH_Fc_), 71.3 (1CH_Fc_), 91.9 (C_Fc_), 125.2, 125.9, 126.4, 127.5, 128.4, 128.8, 128.9, 130.3, 130.9 (15CH_arom_), 136.0, 137.2, 139.3, 144.0 (3C_arom_ and C=N), 170.3 (C=O) ppm. IR: ν 1677 (C=O, s), 1609 (m), 1492 (m), 1444 (m), 1381 (m), 1282 (vs), 1244 (m), 1165 (m), 1108 (m), 988 (m), 820 (m), 726 (vs), 689 (vs) cm^−1^. C_30_H_24_FeN_2_OS_2_ (548.50): calcd. C 65.69, H 4.41, N 5.11, S 11.69; found C 65.70, H 4.51, N 5.21, S 11.42.

*2-Cyclopropyl*-*2*-*ferrocenyl-3-(p-tolylsulfonyl)-6H*-*1,3,4*-*thiadiazine* (**9o**)*:* Yield: 290 mg (48%). Yellowish crystals (petroleum ether/CH_2_Cl_2_). M.p. 105‒106 °C (decomp.). ^1^H-NMR: δ 0.70–0.82 (m, 4CH_Cp_), 0.83–0.91 (m, 1CH_Cp_), 1.06–1.13 (m, 1CH_Cp_), 2.33–2.41 (m, 1CH_CP_), 2.42 (s, CH_3_), 3.41, 3.90 (AB system, *J* = 16.7 Hz, CH_2_), 4.23–4.25 (m, 2CH_Fc_), 4.28–4.29 (m, 1CH_Fc_), 4.31 (s, 5CH_Fc_), 4.77–4.78 (m, 1CH_Fc_), 7.22, 7.68 (*AB* system, *J*_H,H_ = 8.2 Hz, 4CH_arom_), 7.36–7.42 (m, 3CH_arom_), 7.59–7.61 (m, 2CH_arom_). ^13^C-NMR: δ 1.8, 4.3 (2CH_2(Cp)_), 21.0 (CH_Cp_), 21.8 (CH_3_), 24.2 (CH_2_), 66.8 (C_q_), 66.9, 68.1, 68.2, 69.8 (4CH_Fc_), 70.7 (5CH_Fc_), 90.3 (C_Fc_), 125.5, 128.4, 128.5, 128.8, 129 (9CH_arom_), 137.2, 137.4, 139.1, 141.2 (3C_arom_ and C=N). IR: ν 1608 (m), 1440 (m), 1346 (s), 1170 (s), 1092 (s), 1006 (m), 943 (m), 816 (m), 753 (m), 690 (s), 667 (vs) cm^−1^. C_29_H_28_FeN_2_O_2_S_2_ (556.52): calcd. C 62.59, H 5.07, N 5.03, S 11.52; found C 62.22, H 5.23, N 5.20, S 11.37.

*3-Benzoyl-2-cyclopropyl*-*2*-*ferrocenyl-5-phenyl-6H*-*1,3,4*-*thiadiazine* (**9p**)*:* Yield: 125 mg (23%). Orange thick oil. ^1^H-NMR: δ 0.6–0.9 (m, 4CH_Cp_), 2.32–2.34 (m, 1CH_Cp_), 3.59, 4.09 (*AB* system, ^2^*J*_H,H_ = 17.1 Hz, CH_2_), 4.24–4.26 (m, 2CH_Fc_), 4.35 (s, 5CH_Fc_), 4.40–4.41 (m, 1CH_Fc_), 4.75–4.77 (m, 1CH_Fc_), 7.27–7.30 (m, 3CH_arom_), 7.36–7.39 (m, 4CH_arom_), 7.42–7.43 (m, 1CH_arom_), 7.55–7.57 (m, 2CH_arom_). ^13^C-NMR: δ 1.7, 3.9 (2*C*H_2(Cp)_), 21.2 (C*H*_Cp_), 24.6 (CH_2_), 66.6, 66.7, 68.4, 69.6, 69.7 (9*C*H_Fc_), 67.3 (C_q_), 91.7 (C_Fc_), 125.1, 127.3, 128.4, 128.8, 129.4, 130.1 (10CH_arom_), 137.2, 137.4, 139.1 (2C_arom_ and C=N), 170.9 (C=O). IR: ν 1677 (C=O, s), 1613 (m), 1446 (m), 1405 (m), 1341 (m), 1285 (vs), 1241 (m), 1166 (m), 1107 (m), 1073 (m), 1028 (m), 950 (m), 820 (m), 758 (s), 693 (vs) cm^−1^. C_29_H_26_FeN_2_OS (506.45): calcd. C 68.78, H 5.17, N 5.53, S 6.33; found C 68.58, H 5.13, N 5.80, S 6.34.

*1,1,3,3*-*Tetramethyl*-*7*-*phenyl*-*5*-*thia*-*8,9*-*diazaspiro[3.5]nona*-*6,8*-*dien*-*2*-*one* (**11a**): Yield: 160 mg (56%). Orange crystals (petroleum ether/CH_2_Cl_2_). M.p. 93–95 °C. ^1^H-NMR: δ 1.34, 1.39 (2s, 4CH_3_), 6.54 (s, CH-S), 7.41–7.42 (m, 1CH_arom_), 7.46–7.49 (m, 2CH_arom_), 7.77–7.79 (m, 2CH_arom_) ppm. ^13^C-NMR: δ 21.5, 21.6 (4CH_3_), 64.7, 70.0 (3C_q_), 107.4 (CH-S), 125.0, 128.8, 129.1 (5CH_arom_), 134.7, 151.8 (C_arom_ and C=N), 217.6 (C=O) ppm. IR: ν 2965 (m), 1785 (vs), 1446 (m), 1422 (m), 1362 (m), 1203 (m), 1041 (m), 1030 (m), 1002 (m), 747 (vs), 691 (vs), 656 (s) cm^−1^. UV-Vis: λ_1_: 239 (ε_1_ = 1.3 × 10^4^); λ_2_: 283 (ε_2_ = 3.7 × 10^3^), λ_3_: 358 (ε_3_ = 7.4 × 10^2^)_)_; λ_4_: 430 (ε_4_ = 1.6 × 10^2^) nm. MS-ESI: *m/z* 287 (100, [M + 1]^+^), 309 (18, [M + Na]^+^). C_16_H_18_N_2_OS (286.39): calcd. C 67.10, H 6.34, N 9.78, S 11.19; found C 67.06, H 6.33, N 9.75, S 11.15.

*1,1,3,3-Tetramethyl-7-phenyl-5-thia-8,9-diazaspiro[3.5]nona-6,8-diene-2-thione* (**11b**): Yield: 210 mg (69%). Orange crystals (petroleum ether/CH_2_Cl_2_). M.p. 71–74 ^○^C. ^1^H-NMR: δ 1.40, 1.45 (2s, 4CH_3_), 6.54 (s, CH-S), 7.40–7.42 (m, 1CH_arom_), 7.47–7.50 (m, 2CH_arom_), 7.8–7.81 (m, 2CH_arom_) ppm. ^13^C-NMR: δ 25.6, 25.7 (4CH_3_), 67.3, 73.3 (3C_q_), 107.3 (CH-S), 124.9, 128.7, 129.0 (5CH_arom_), 134.7, 151.7 (C_arom_ and C=N), 277.4 (C=O) ppm. IR: ν 2961 (m), 2931 (m), 1459 (m), 1429 (m), 1422 (m), 1358 (m), 1300 (s), 1157 (m), 1025 (m), 749 (vs), 691 (vs) cm^−1^. UV-Vis: λ_1_ = 268 (ε_1_ = 1.36 × 10^4^); λ_2_ = 364 (ε_2_ = 1.91 × 10^3^); λ_3_ = 426 (ε_3_ = 5.8 × 10^2^) nm_._ C_16_H_18_N_2_S_2_ (302.45): calcd. C 63.54, H 6.00, N 9.26, S 21.20; found C 63.53, H 5.92, N 9.20, S 21.17.

*3-Benzoyl-2,5-diphenyl-2-styryl-6H-1,3,4-thiadiazine* (**9s**): Yield: 175 mg (38%). Thick yellowish oil. ^1^H-NMR: δ 3.50, 3.62 (*AB* system, ^2^*J*_H,H_ = 17.0 Hz, CH_2_), 6.67, 6.97 (2d, ^3^*J*_H,H_ = 16.0 Hz, 2CH=), 7.28–7.43 (m, 16CH_arom_), 7.69–7.70 (m, 2CH_arom_), 7.89–7.91 (m, 2CH_arom_) ppm. ^13^C-NMR: δ 24.0 (CH_2_), 69.1 (C_q_), 125.3, 126.4, 127.9, 127.5, 127.6, 128.2, 128.3, 128.5, 128.6, 128.7, 129.2, 130.2, 131.0, 131.7 (20*C*H_arom_ and 2*C*H=), 135.5, 135.9, 137.3, 140.6 (3C_arom_ and C=N), 170.2 (C=O) ppm. IR: ν 1669 (C=O, s), 1590 (m), 1492 (m), 1285 (s), 1226 (m), 1148 (m), 1073 (m), 961 (m), 726 (s), 689 (vs) cm^−1^. ESI-MS for C_30_H_25_N_2_OS [M + 1]^+^: cald. mass: 461.1688, found mass: 461.1687. ESI-MS for C_30_H_24_N_2_OSNa [M + Na]^+^: cald. mass: 483.1507, found mass: 483.1509.

## 4. Conclusions

A large number of new reviews [26,27,56,57] and original publications [31,58,59,60,61,62], which appeared in the last four years, demonstrates the growing interest in the chemistry of azoalkenes and their applications in *hetero*-Diels-Alder reactions. The presented study showed important mechanistic aspects and demonstrated that scarcely known 3,6-dihydro-2*H*-1,3,4-thiadiazines can efficiently be prepared by regioselective *hetero*-Diels-Alder reactions of thioketones with in situ-generated azoalkenes, bearing an electron-withdrawing substituent at the terminal N-atom. In comparison to aromatic and ferrocenyl-substituted thioketones, sterically hindered cycloaliphatic representatives differ in their reactivity and no stable cycloadducts were obtained with adamantanethione (**1k**). Interestingly, the primary products derived from cyclobutanethiones **1i**,**j** suffered an elimination of toluene sulfinic acid to provide the rare 2*H*-1,3,4-thiadiazines. Differing stability of the target (4 + 2)-cycloadducts was also observed in experiments with thiochalcone **1l** and azoalkenes **7a** and **7b**, respectively.

A comprehensive computational study demonstrated that the preferred formation of 1,3,4-thiadiazines **9** from azoalkene **7a** and thioketones **1** is best explained by strong kinetic control of the respective cycloaddition over the formation of 1,2,3-thiadiazines **10**. The transition states involved for 1,3,4-thiadizine formation are quite unsymmetric with short C..S and long N..C-distances. In the case of high sterical hindrance at the thioketone carbon, even a two-step pathway via a zwitterionic intermediate could be traced. In contrast, the formation of the unobserved 1,2,3-thiadiazines **10** would involve a concerted *hetero*-Diels-Alder reaction. However, although thermodynamics are not unfavorable for these regioisomers, substantial kinetic barriers prevented formation of **10** under the experimental conditions. The proposed intermediacy of zwitterions suggests further experiments in order to trap these transient species by suitable reagents.

It has to be underlined that 1,3,4-thiadiazines and fused systems containing this motif attract attention as biologically active compounds [63]. However, in spite of the growing interest in the application of thia-Diels-Alder reactions for the synthesis of six-membered S-heterocycles [3,16,21,22,64,65], efficient (4 + 2)-cycloadditions with easily accessible thioketones as well as thiochalcones with in situ-generated, reactive azoalkenes are to date practically unexplored for the preparation of the 1,3,4-thiadiazine skeleton. Therefore, the described experimental and theoretical results open new horizons for the development of syntheses of this type of N,S-heterocycles, including an asymmetric approach in the case of prochiral ferrocenyl/aryl and ferrocenyl/alkyl thioketones.

In general, one can conclude that—in spite of the fact that the first study by Diels and Alder was published almost 100 years ago [66,67]—the exploration of (4 + 2)-cycloaddition reactions [68] as a universal tool for construction of six-membered carbo- and heterocyclic systems still inspires new generations of chemists for the development of new methods in organic synthesis and offers a plethora of theoretical problems, which are of crucial importance for better understanding the mechanisms of organic reactions.

## Figures and Tables

**Table 1:** DFT calculations of the cycloadditions of azoalkene **7a** with different thioketones **1** (Gibbs free energies (ΔG_298_) in kcal/mol).

Entry	Starting Materials	Van der Waals Complexes	Transition State A	1,3,4-Thiadiazine 9	Transition State B	1,2,3-Thiadiazine 10
1	**7a + 1a** [0.0]	4.3	8.2	−20.8	19.3	−19.3
2	**7a + 1b** [0.0]	3.0	10.0	−24.0	17.6	−22.7
3	**7a + 1m** [0.0]	3.3	13.1	−15.6	33.6	−18.7
4	**7a + 1n** [0.0]	3.9	11.9	−19.4	33.5	−15.3
5	**7a + 1i** [0.0]	4.3	13.2 [a] 12.0	−19.1	25.9	−22.8
6 [b]	**7a + 1i** [0.0]	4.3	32.1	−4.1 [c]	38.8	13.8 [d]

[a] See discussion and Scheme 9; [b] Addition to the CO double bond of **1i**; [c] 1,3,4-Oxadiazine; [d] 1,2,3-Oxadiazine.

**Table 2:** DFT-calculated distances of the reacting atoms in the transition states **A** or **B** for the cycloadditions of azoalkene **7a** with different thioketones **1** leading to **9** or **10**.

Entry	Starting Materials	Transition State A to 1,3,4-Thiadiazine 9 TS-Distances (Å)		Transition State B to 1,2,3-Thiadiazine 10 TS-Distances (Å)	
		C..S	N..C	C..C	S..N
1	**1a + 7a** [0.0]	2.578	3.199	2.493	2.151
2	**1b + 7a** [0.0]	2.393	3.097	2.370	2.226
3	**1m + 7a** [0.0]	2.319	3.029	2.471	2.342
4	**1n + 7a** [0.0]	2.373	3.044	2.546	2.190
5	**1i + 7a**	2.276	3.573	2.427	2.366
		1.833	2.529		
6	**1i + 7a** [a]	1.695 [b]	3.214 [b]	2.770 [c]	1.939 [c]

[a] Addition to the CO double bond of **1i**; [b] TS for 1,3,4-oxadiazine; [c] TS for 1,2,3-oxadiazine.

## Data Availability

Reported data are available at Authors via e-mail contact.

## Supplementary Materials

The Supplementary Materials are available online and they contain the scanned ^1^H and ^13^C NMR spectra for the described and isolated compounds. The X-ray crystallography data for compounds **9c** and **9g** are deposited as CSD Communication under deposition number 2072033 and 2072034.
