# Supplementary material for: Hetero-Diels-Alder Reactions of In Situ-Generated Azoalkenes with Thioketones; Experimental and Theoretical Studies
Source: Molecules. 2021 Apr 27;26(9):2544. doi: 10.3390/molecules26092544 (PMC8123831; doi:10.3390/molecules26092544)
Supplement: Supplementary file 1 [file molecules-26-02544-s001.zip › molecules-1188426-supplementary.pdf]

Supplementary Information

Hetero-Diels-Alder Reactions of in situ-Generated Azoalkenes with Thioketones; Experimental and Theoretical Studies

Grzegorz Mlostoń [1,*], Katarzyna Urbaniak [1], Malwina Sobiecka [1], Heinz Heimgartner [2], Ernst-Ulrich Würthwein [3,*], Reinhold Zimmer [4], Dieter Lentz [4] and Hans-Ulrich Reissig [4,*]

[1] Department of Organic and Applied Chemistry, Faculty of Chemistry, University of Lodz, 12 Tamka Street, 91-403 Lodz, Poland; katarzyna.urbaniak@chemia.uni.lodz.pl; melkies@wp.pl
[2] Department of Chemistry, University of Zurich, Winterthurerstrasse 190, CH-8057 Zurich, Switzerland; heinz.heimgartner@chem.uzh.ch
[3] Organisch-Chemisches Institut and Center for Multiscale Theory and Computation (CMTC), Westfälische Wilhelms-Universität Münster, Corrensstrasse 40, 48149 Münster, Germany
[4] Institut für Chemie und Biochemie, Freie Universität Berlin, Takustrasse 3, 14195 Berlin, Germany: rzimmer@chemie.fu-berlin.de; dieter.lentz@fu-berlin.de
* Correspondence: grzegorz.mloston@chemia.uni.lodz.pl; Tel.:+48-42-635-57-61; wurthwe@uni-muenster.de; hreissig@chemie.fu-berlin.de

E-mail: grzegorz.mloston@chemia.uni.lodz.pl

Table of Contents

1. Experimental: Generals page SI-2
1.1. The ^{1}H NMR and ^{13}C NMR spectra of all new compounds **9a-p**, **9s** and **11a-b** page SI-2
1.2. The UV-Vis spectra for products **11a-b** page SI-22
2. The X-Ray structure determinations page SI-24
3. DFT calculations page SI-25
3.1. Gaussian Archive Entries page SI-27
4. References page SI-64

1. Experimental

Generals: For general information and preparation procedures, see the main manuscript, pp. 12-13.

1.1. The ^{1}H NMR and ^{13}C NMR spectra of all new compounds 9a-p, 9s and 11a-b:

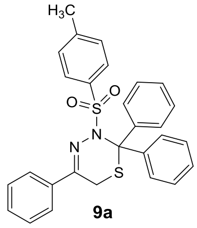

9a

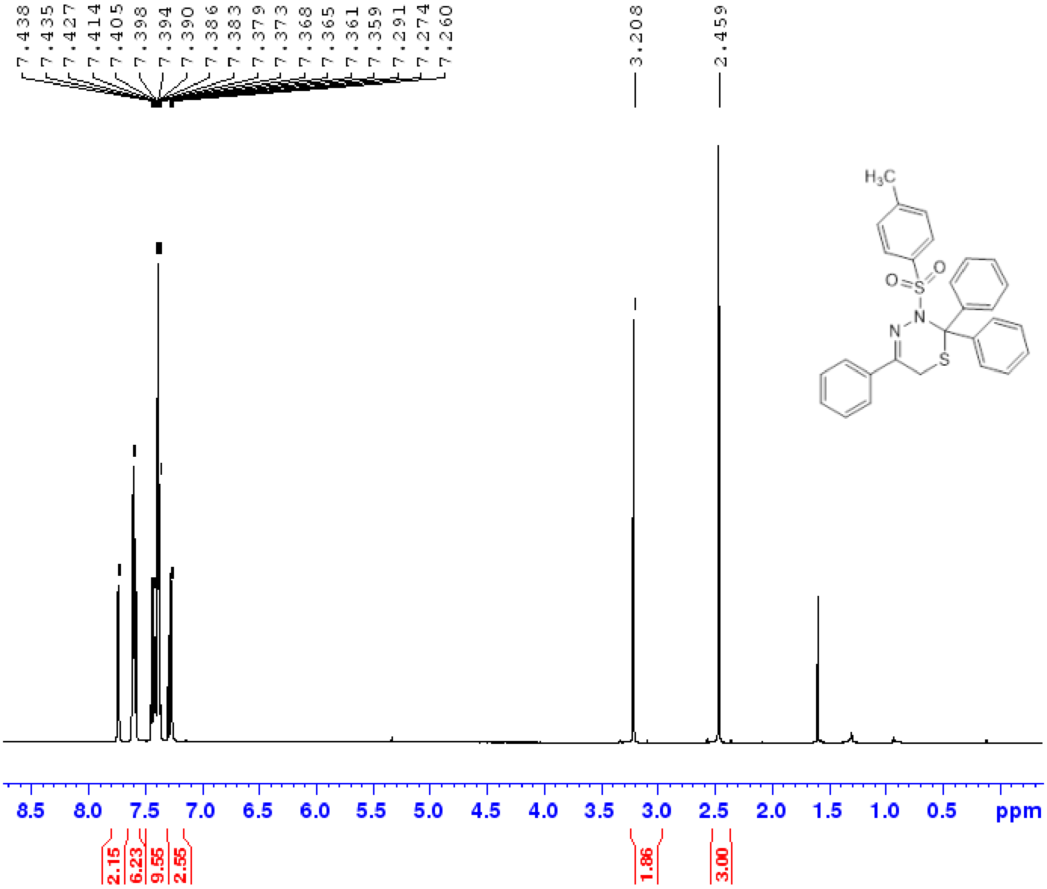

Fig. 1a. ^{1}H-NMR for compound **9a**.

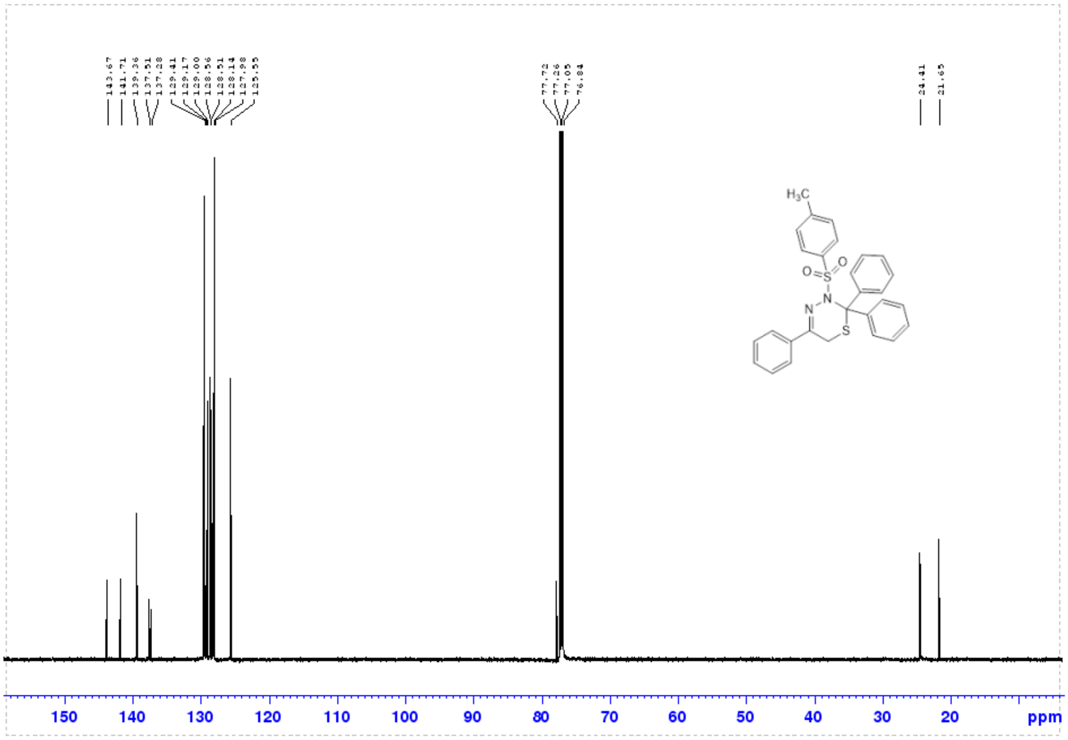

Fig. 1b. ^{13}C-NMR for compound **9a**

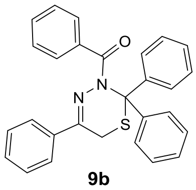

9b

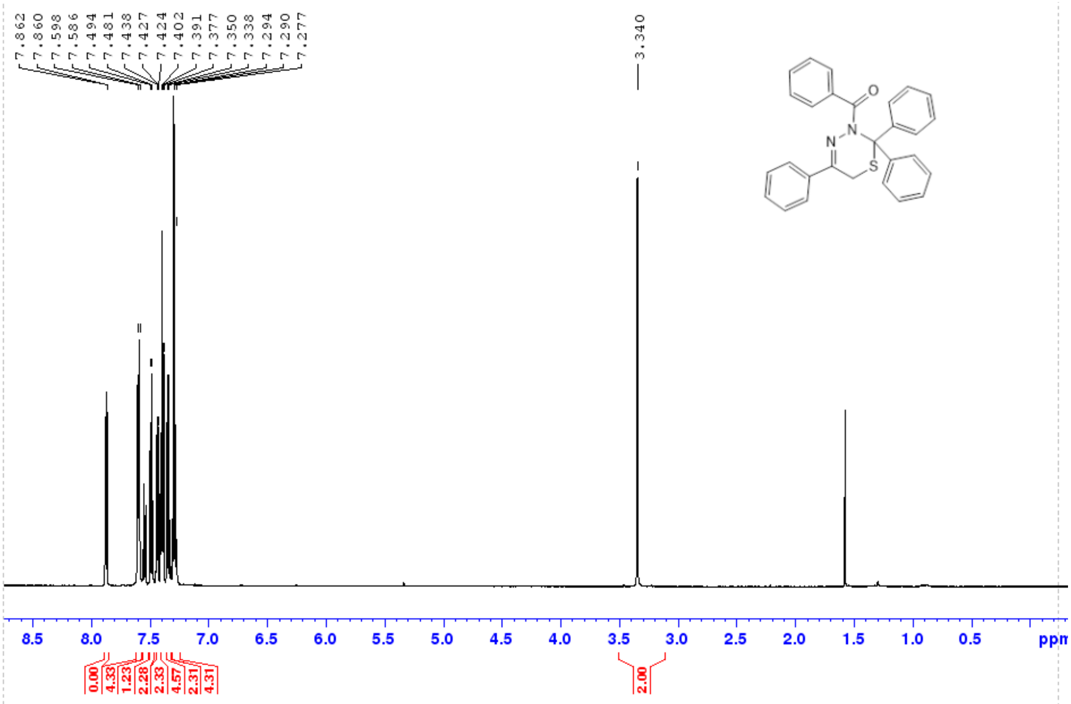

Fig. 2a. ^{1}H-NMR for compound **9b**.

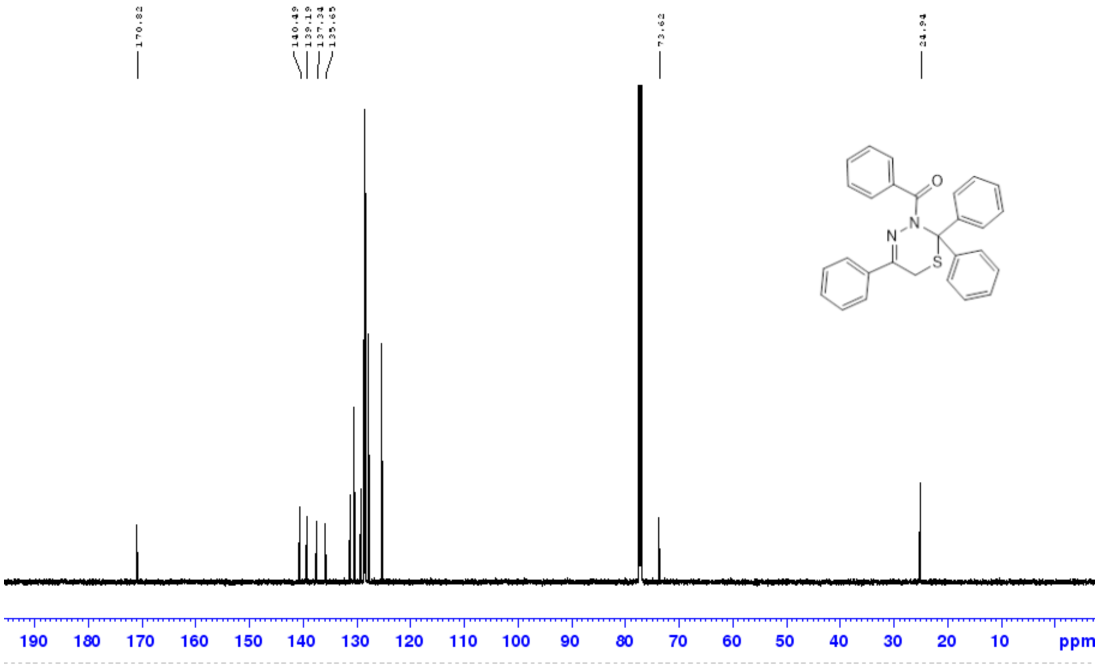

Fig. 2b. ^{13}C NMR for compound **9b**.

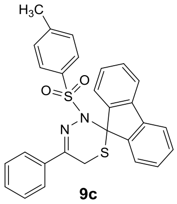

9c

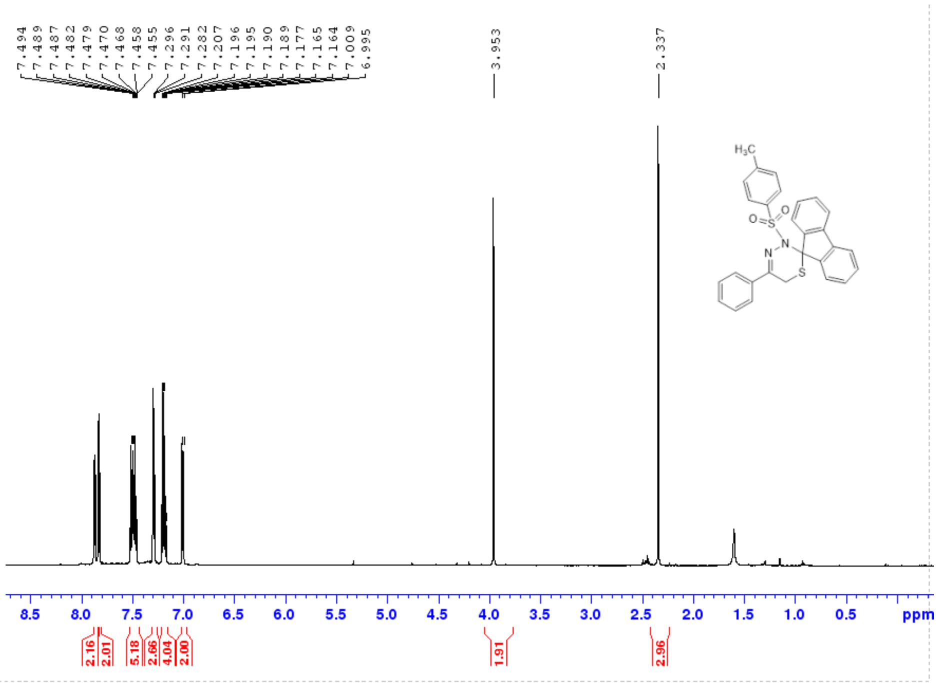

Fig. 3a. ^{1}H NMR for compound **9c**.

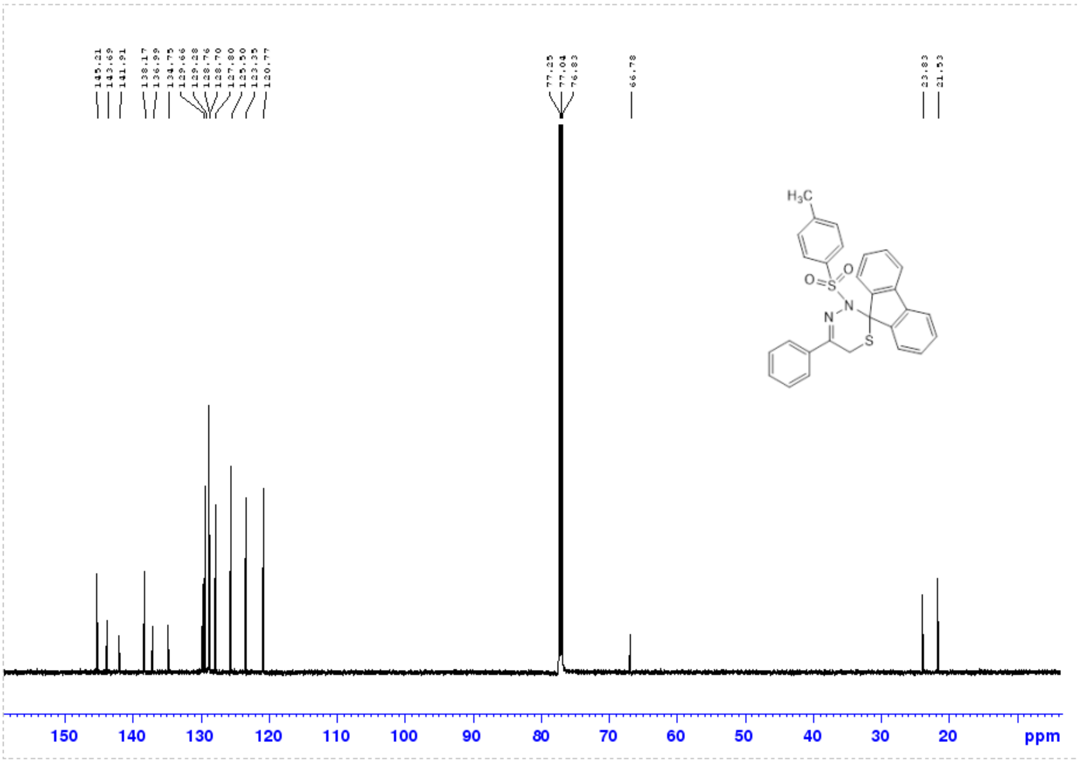

Fig. 3b. ^{13}C NMR for compound **9c**.

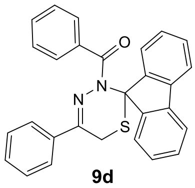

9d

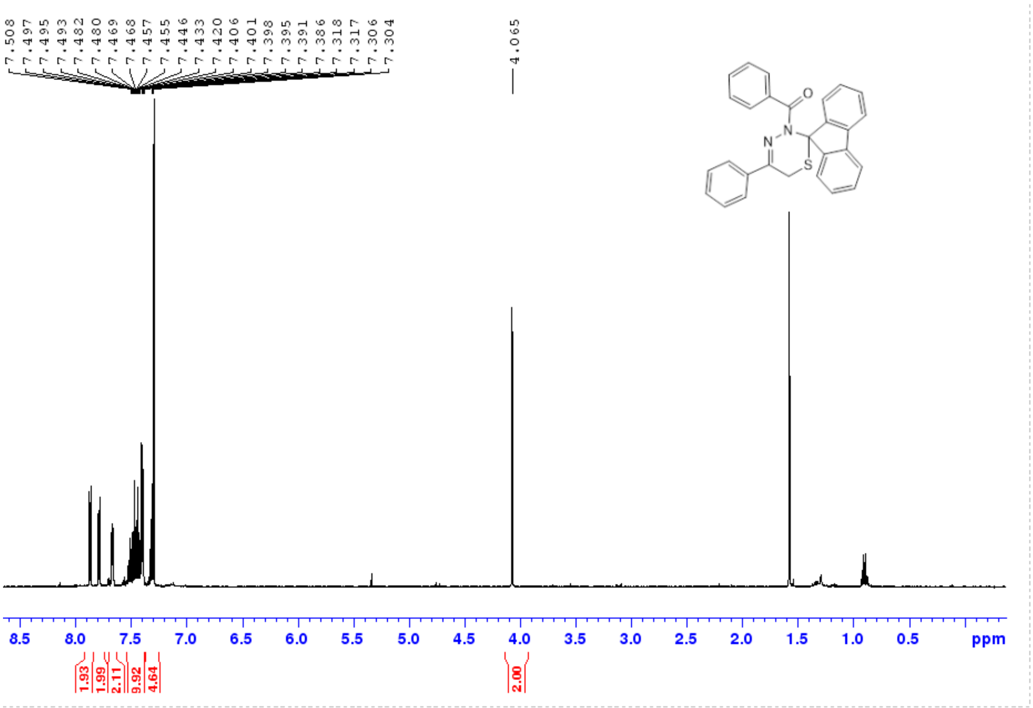

Fig. 4a. ^{1}H NMR for compound **9d**.

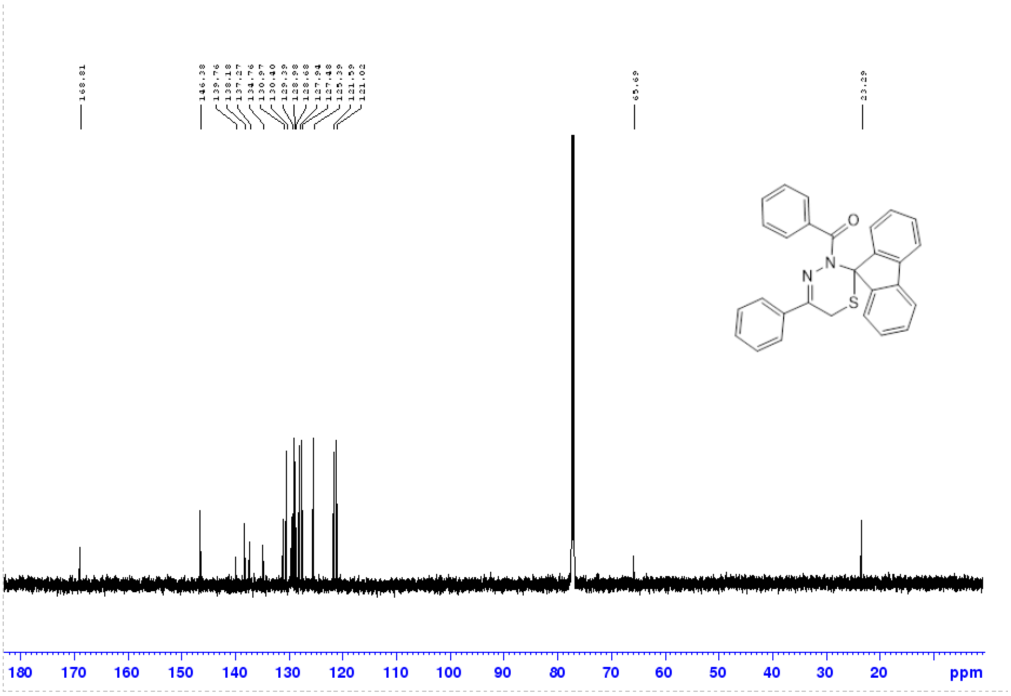

Fig. 4b. ^{13}C NMR for compound **9d**.

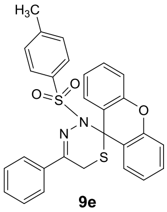

9e

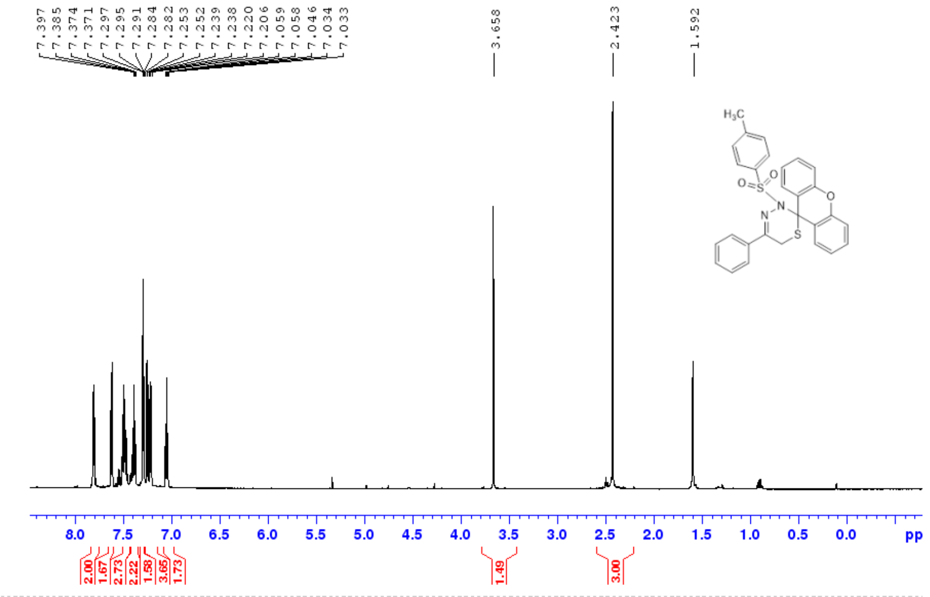

Fig. 5a. ^{1}H NMR for compound **9e**.

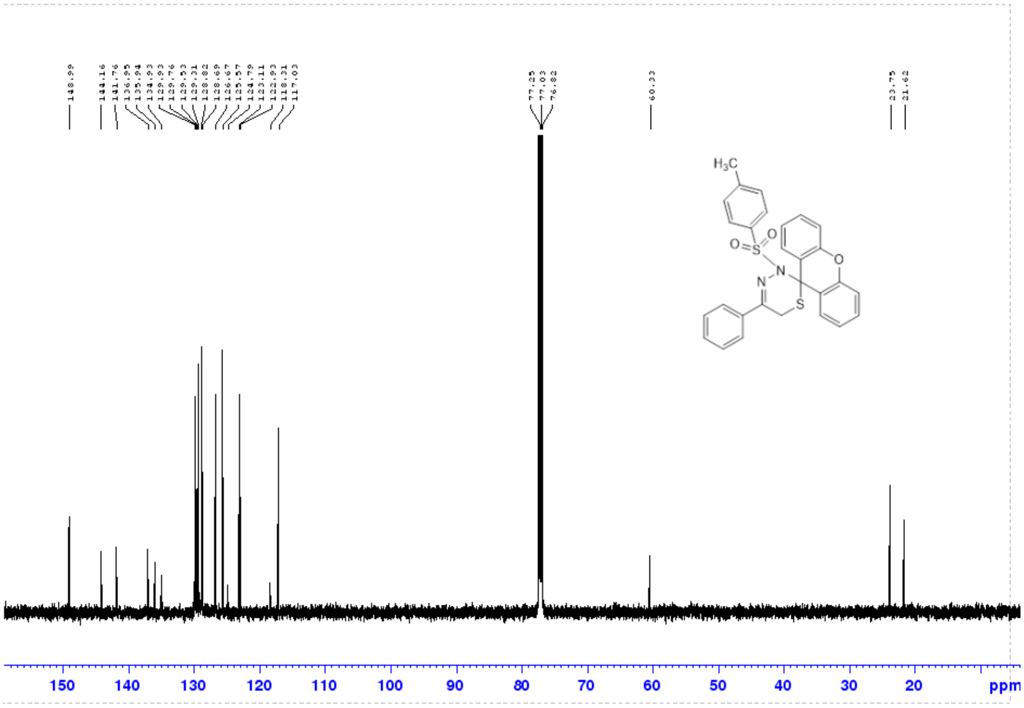

Fig. 5b. ^{13}C NMR for compound **9e**.

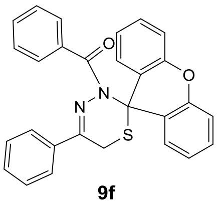

9f

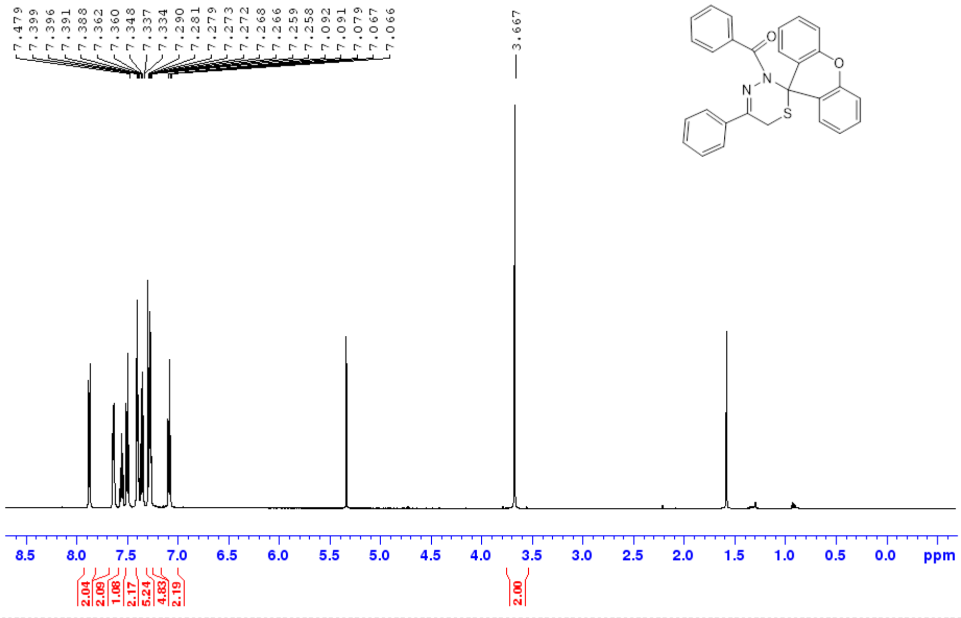

Fig. 6a. ^{1}H NMR for compound **9f**.

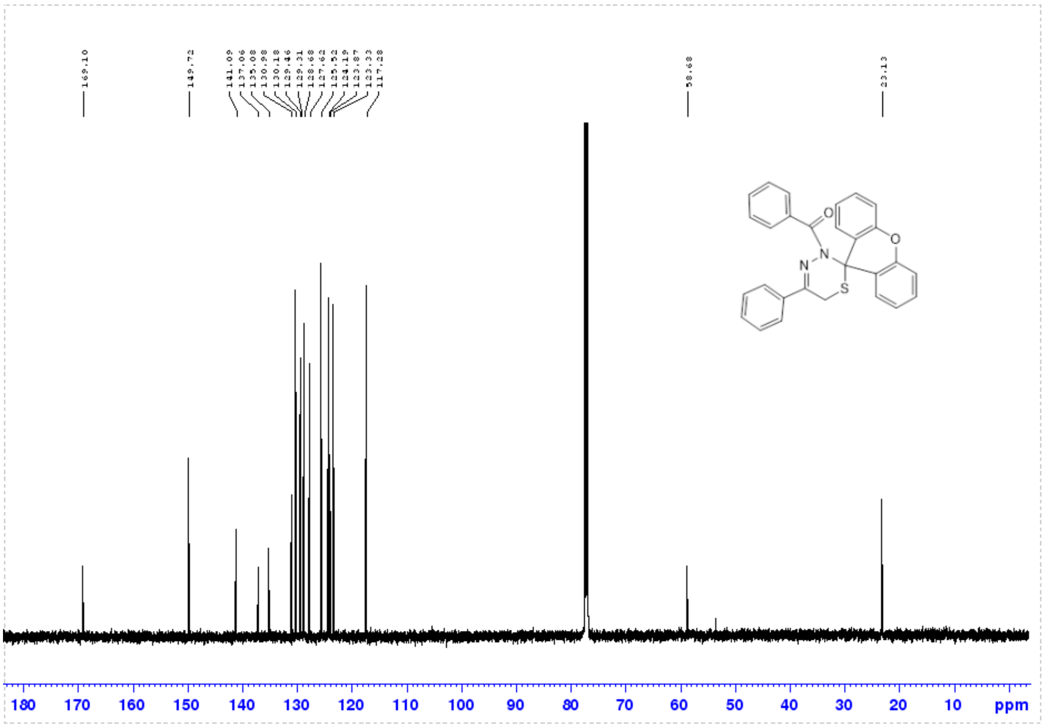

Fig. 6b. ^{13}C NMR for compound **9f**.

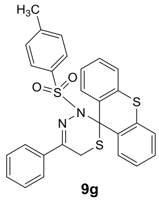

9g

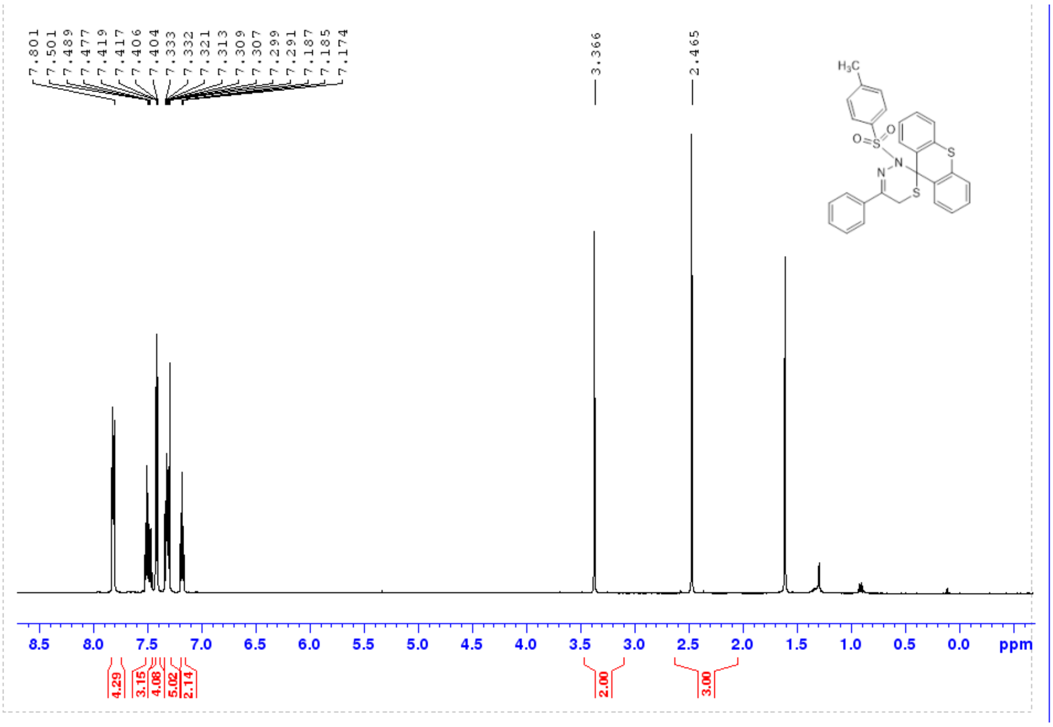

Fig. 7a. ^{1}H NMR for compound **9g**.

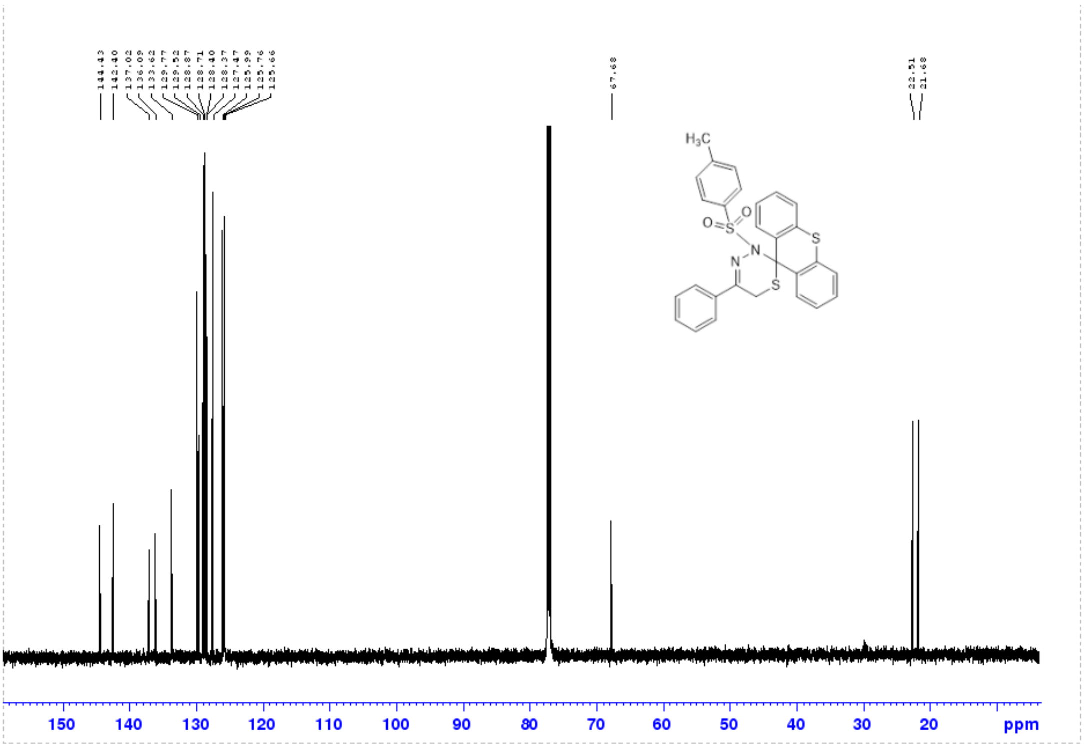

Fig. 7b. ^{13}C NMR for compound **9g**.

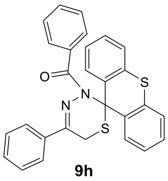

9h

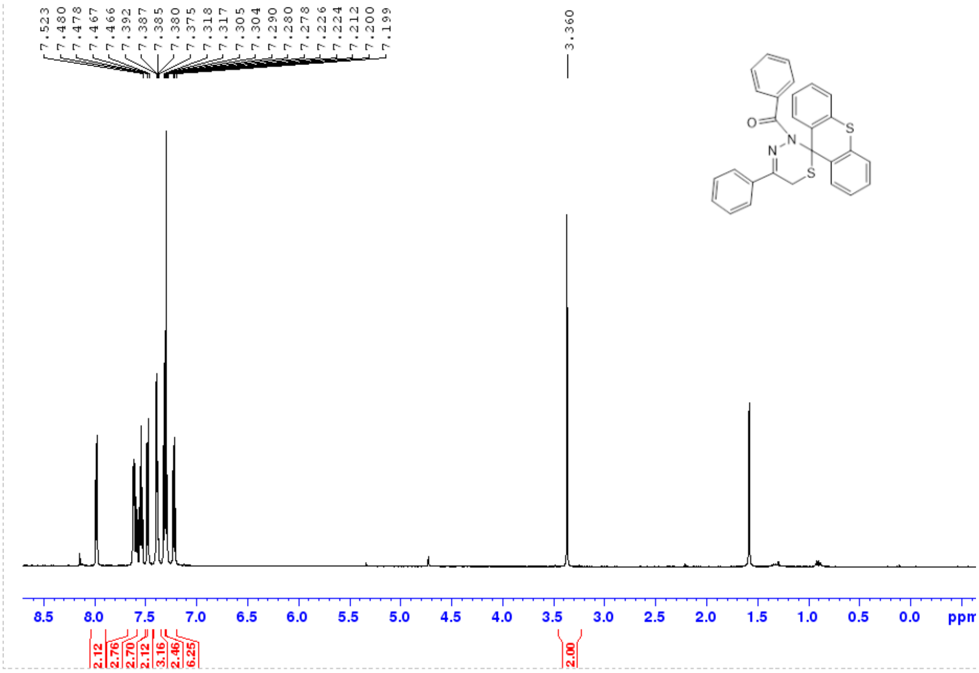

Fig. 8a. ^{1}H NMR for compound **9h**.

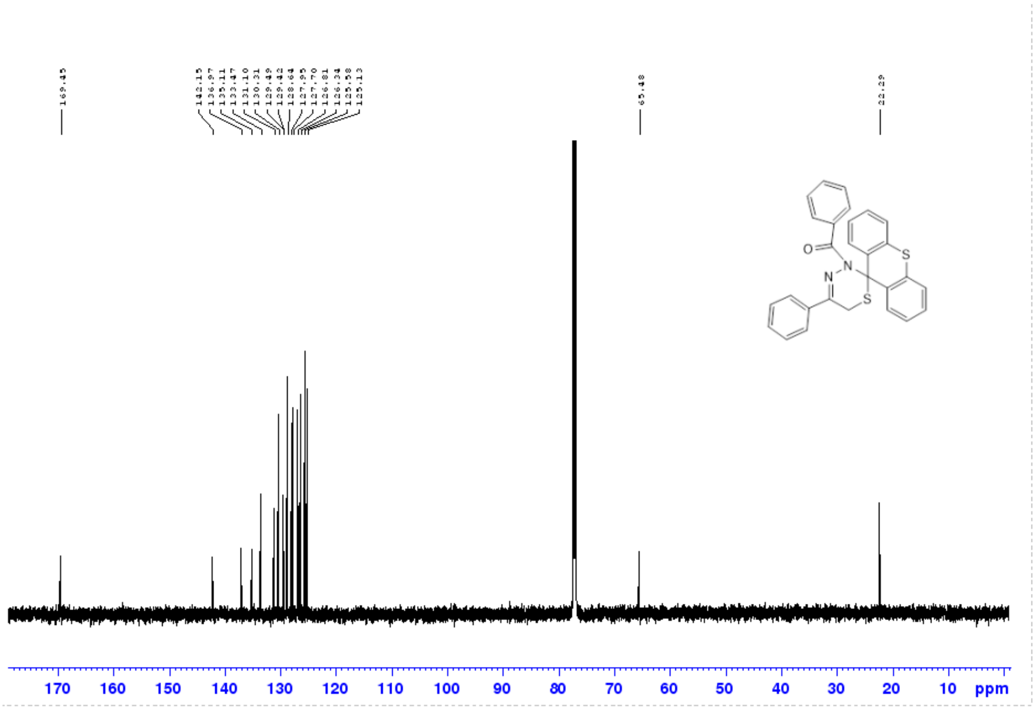

Fig. 8b. ^{13}C NMR for compound **9h**.

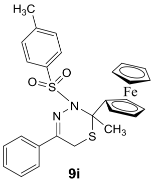

9i

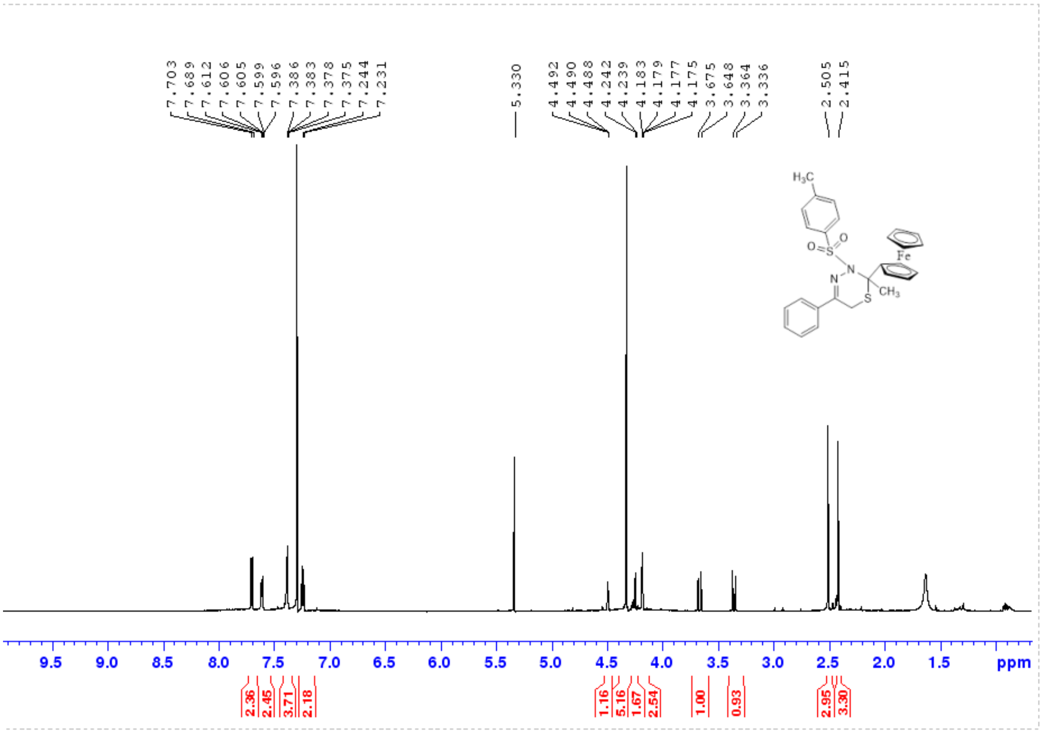

Fig. 9a. ^{1}H NMR for compound **9i**.

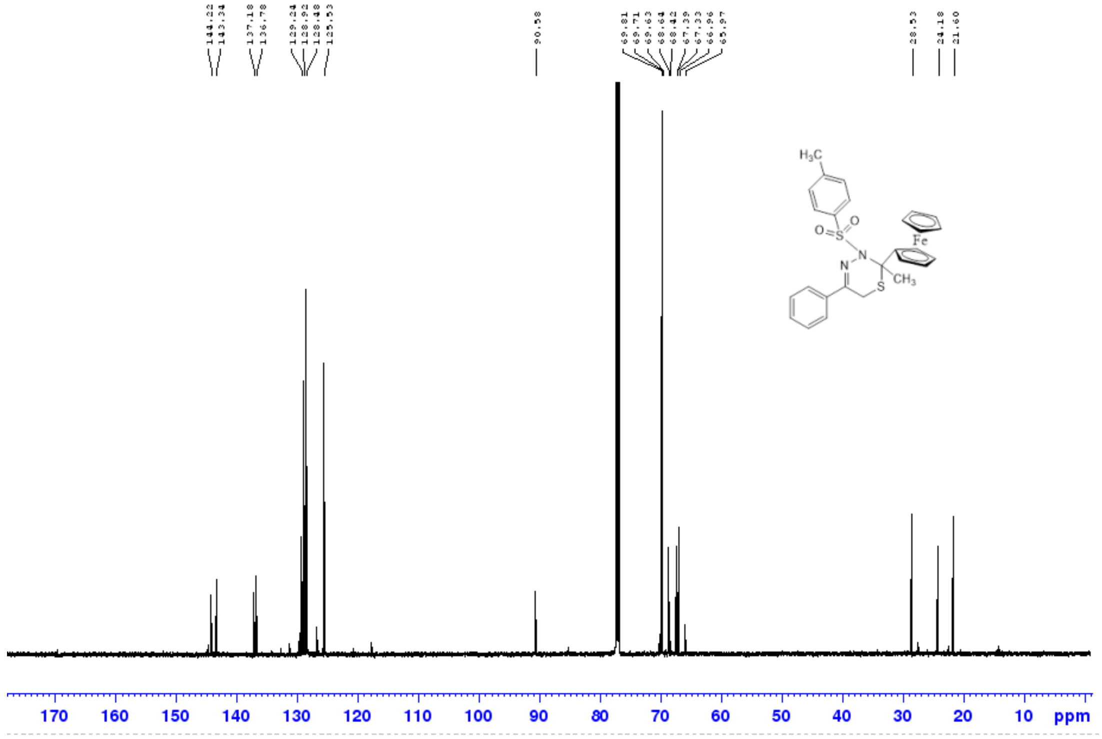

Fig. 9b. ^{13}C NMR for compound **9i**

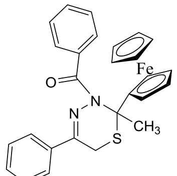

9j

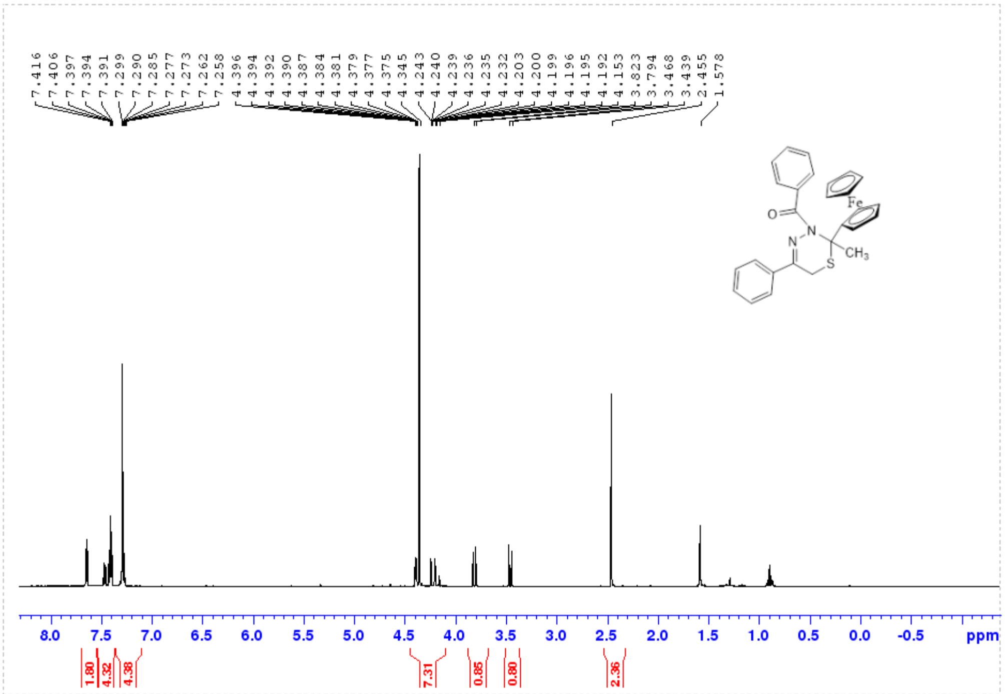

Fig. 10a. ^{1}H NMR for compound **9j**.

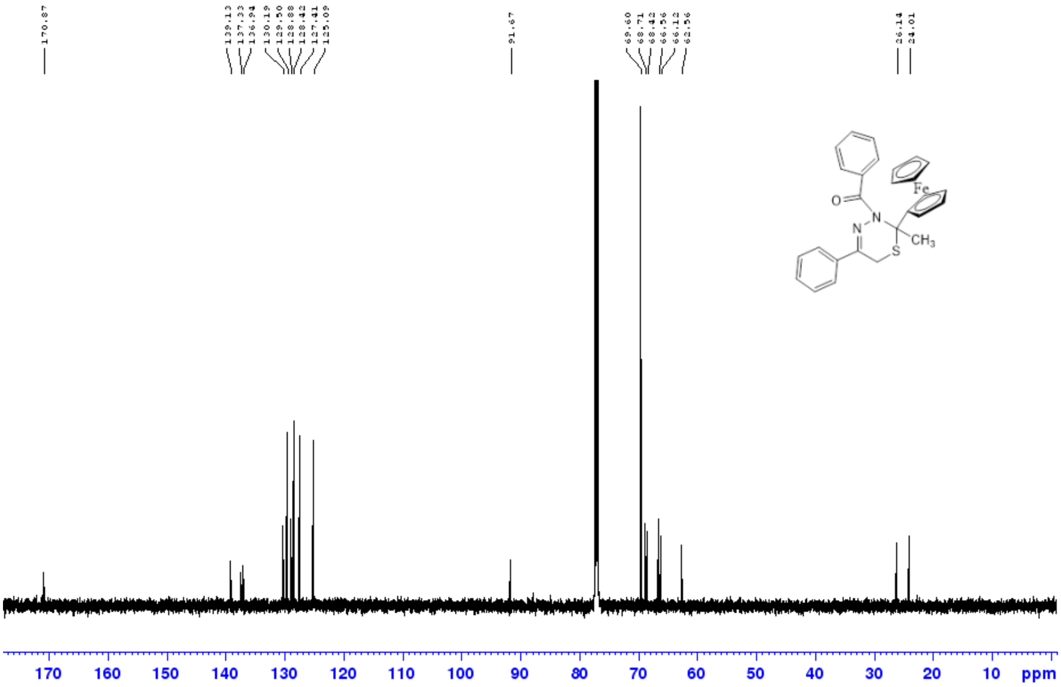

Fig. 10b. ^{13}C NMR for compound **9j**.

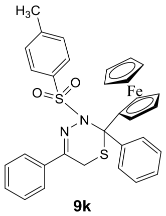

9k

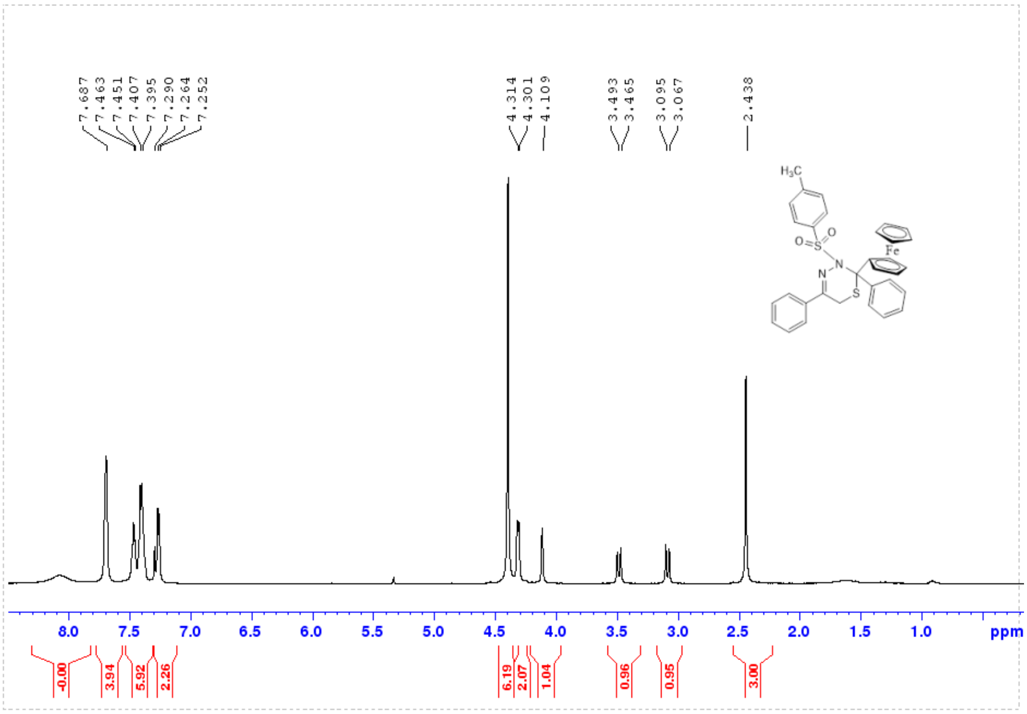

Fig. 11a. ^{1}H NMR for compound **9k**.

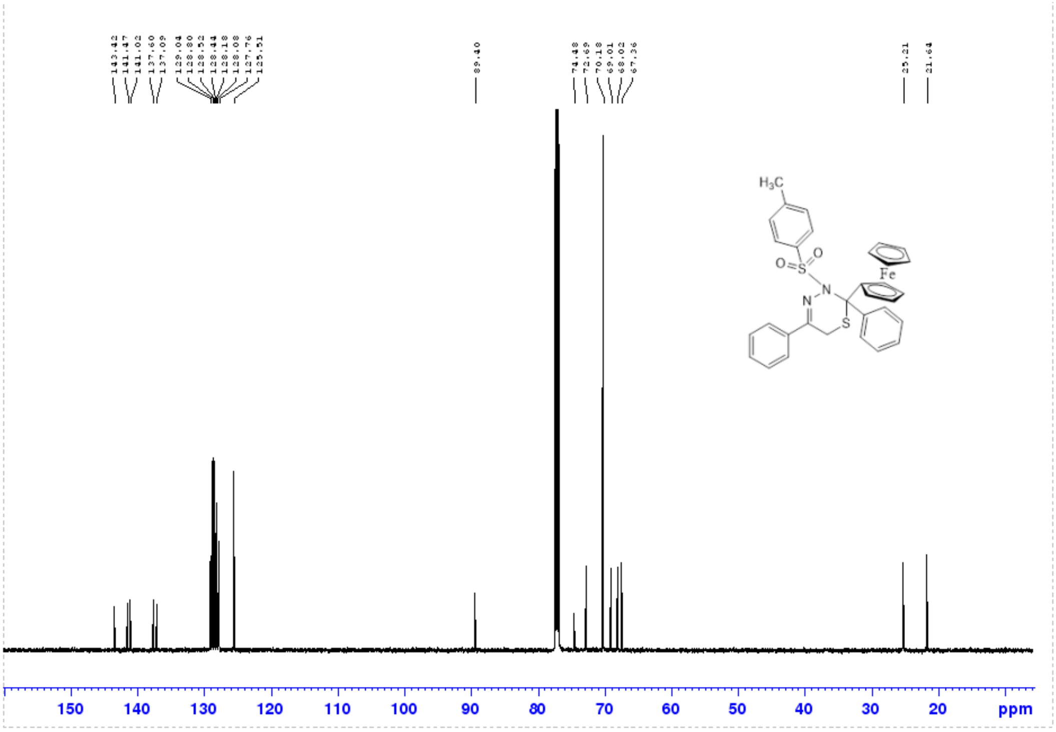

Fig. 11b. ^{13}C NMR for compound **9k**.

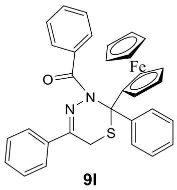

9l

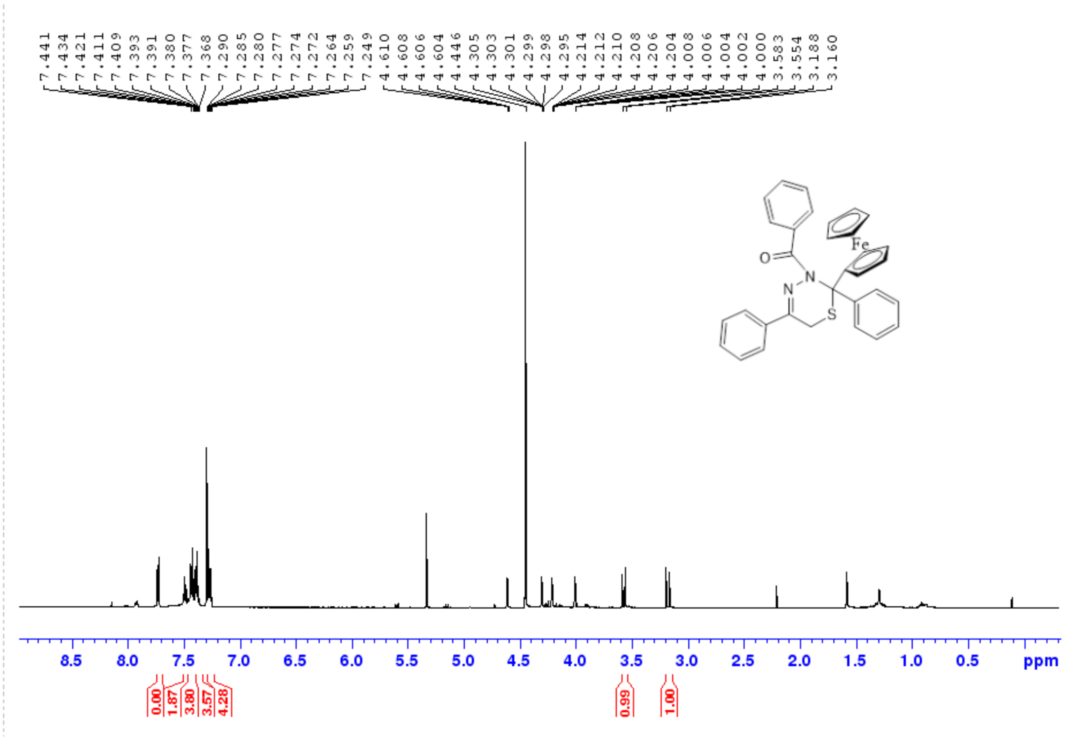

Fig. 12a. ^{1}H NMR for compound **9l**.

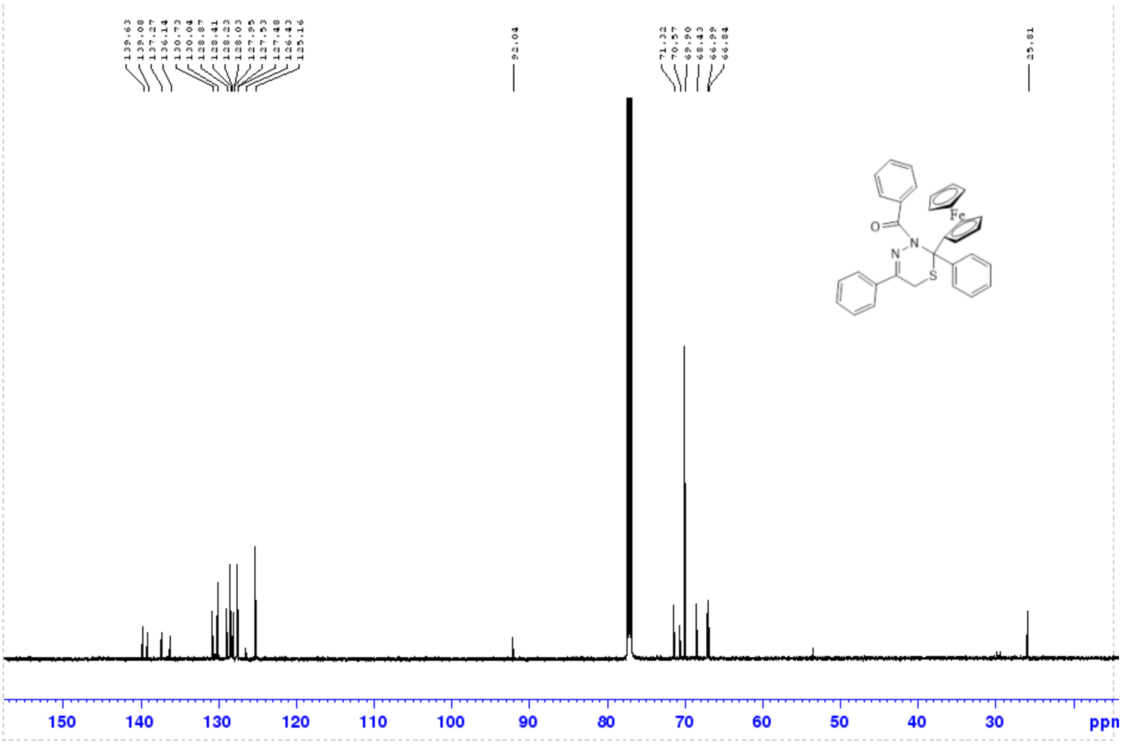

Fig. 12b. ^{13}C NMR for compound **9l**.

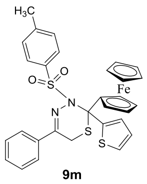

9m

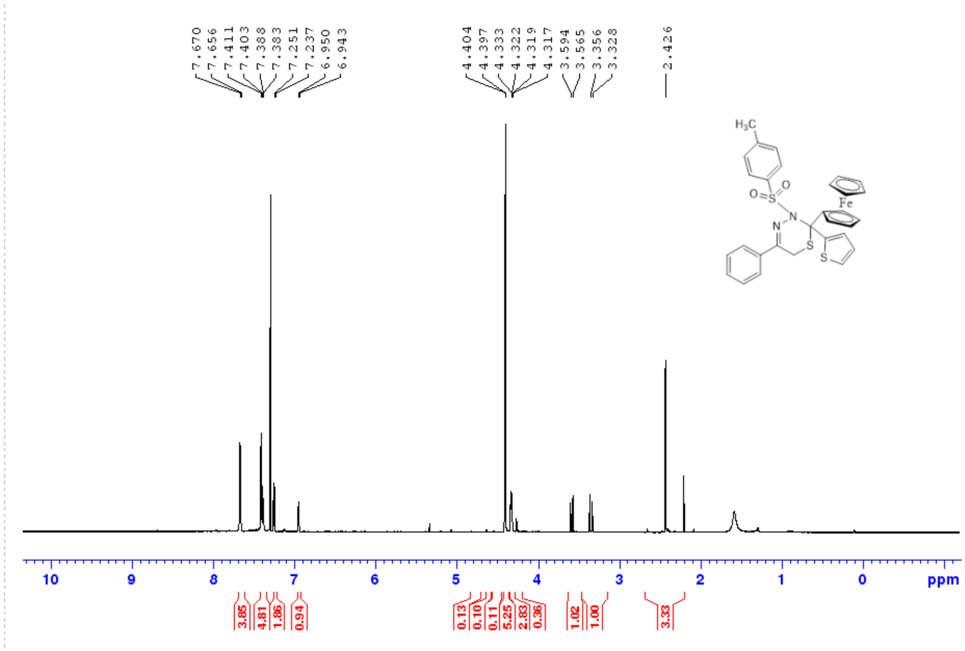

Fig. 13a. ^{1}H NMR for compound **9m**.

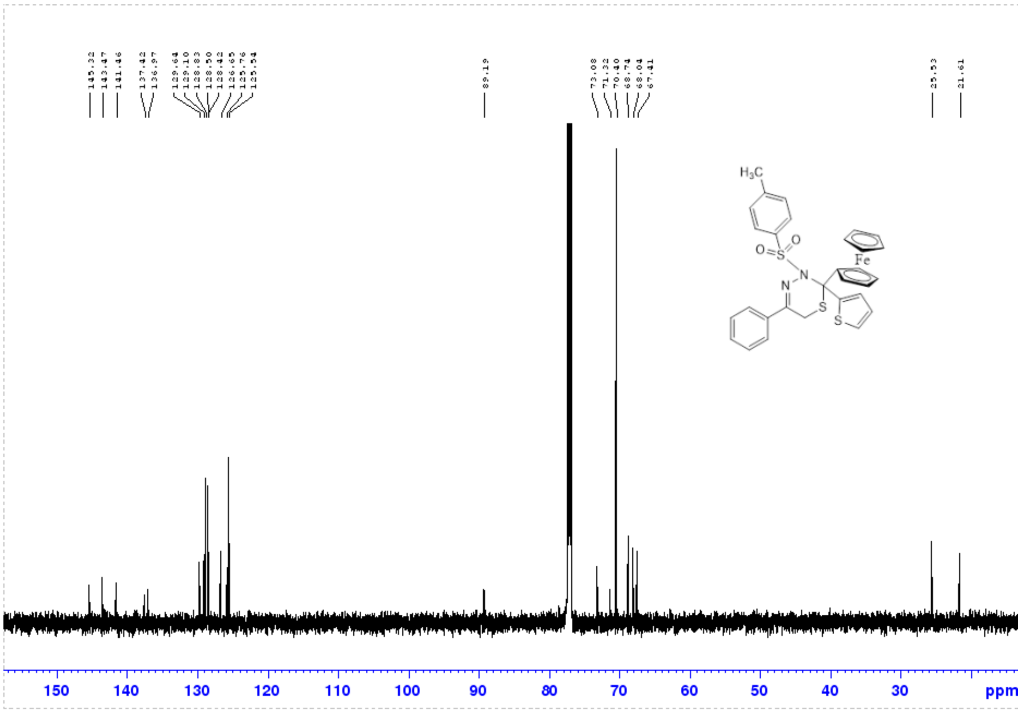

Fig. 13b. ^{13}C NMR for compound **9m**.

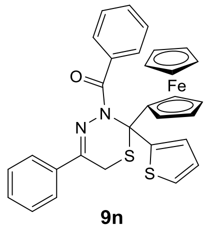

9n

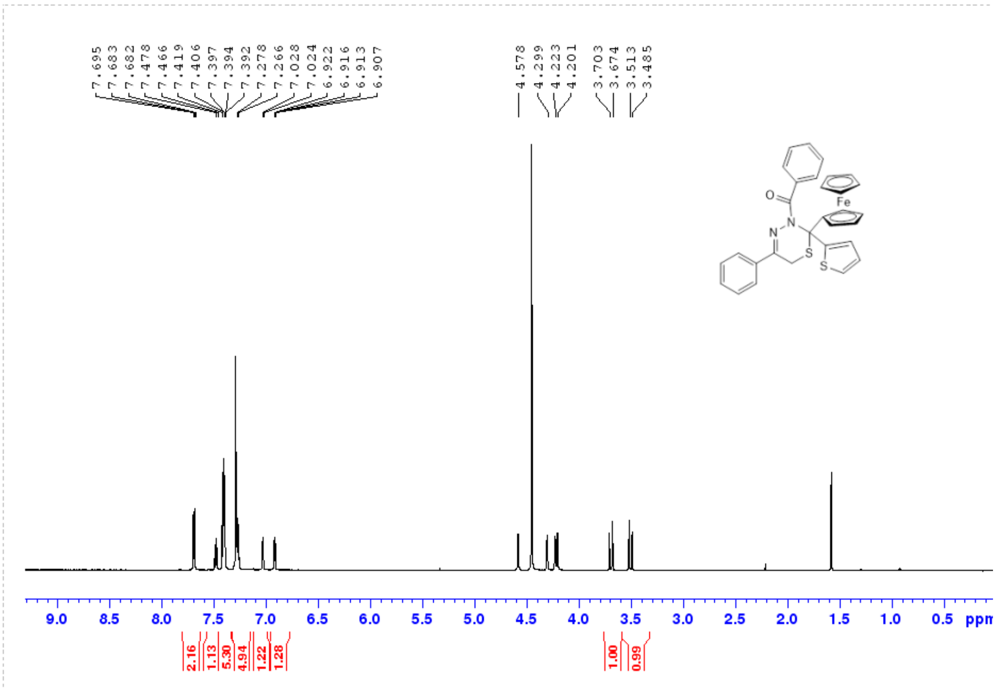

Fig. 14a. ^{1}H NMR for compound **9n**.

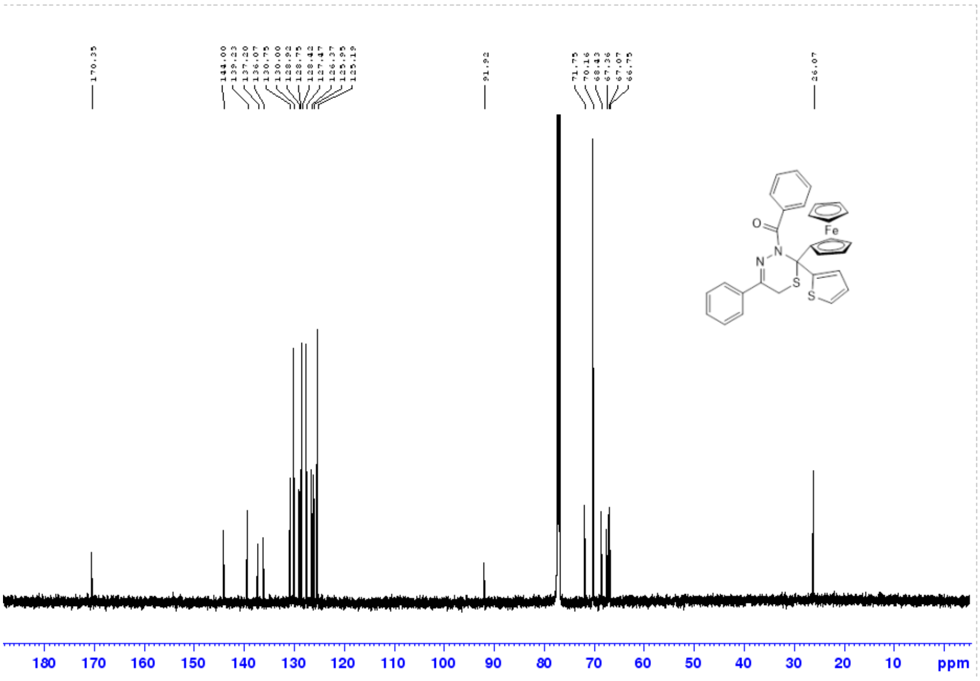

Fig. 14b. ^{13}C NMR for compound **9n**.

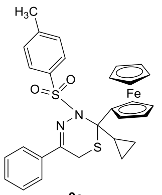

9o

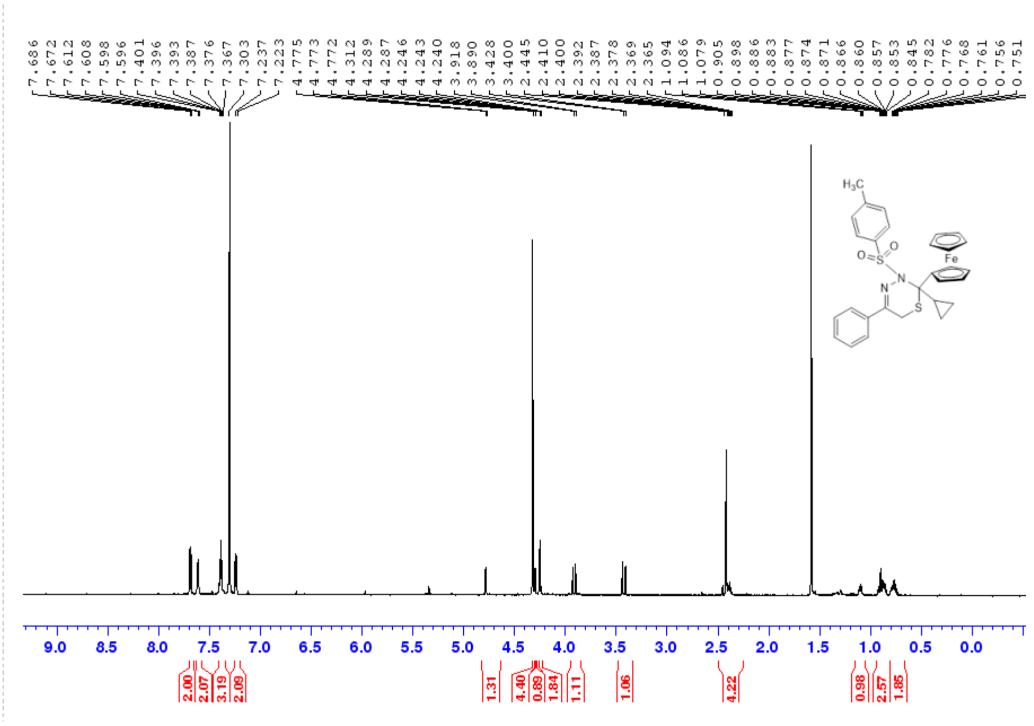

Fig. 15a. ¹H NMR for compound **9o**.

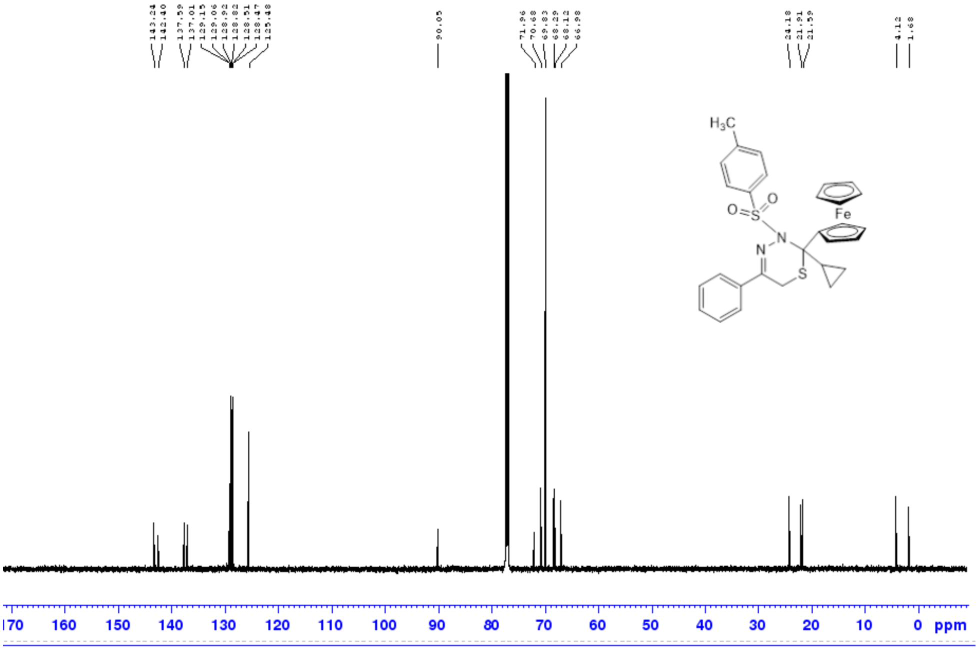

Fig. 15b. ¹³C NMR for compound **9o**.

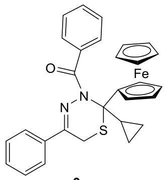

9p

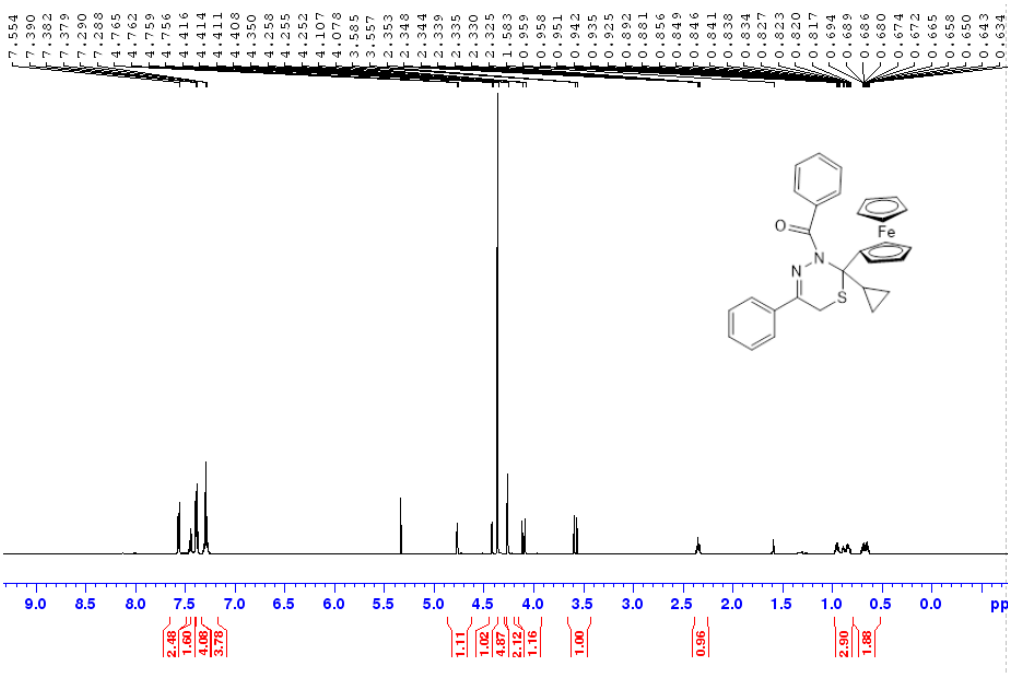

Fig. 16a. ^{1}H-NMR for compound **9p**.

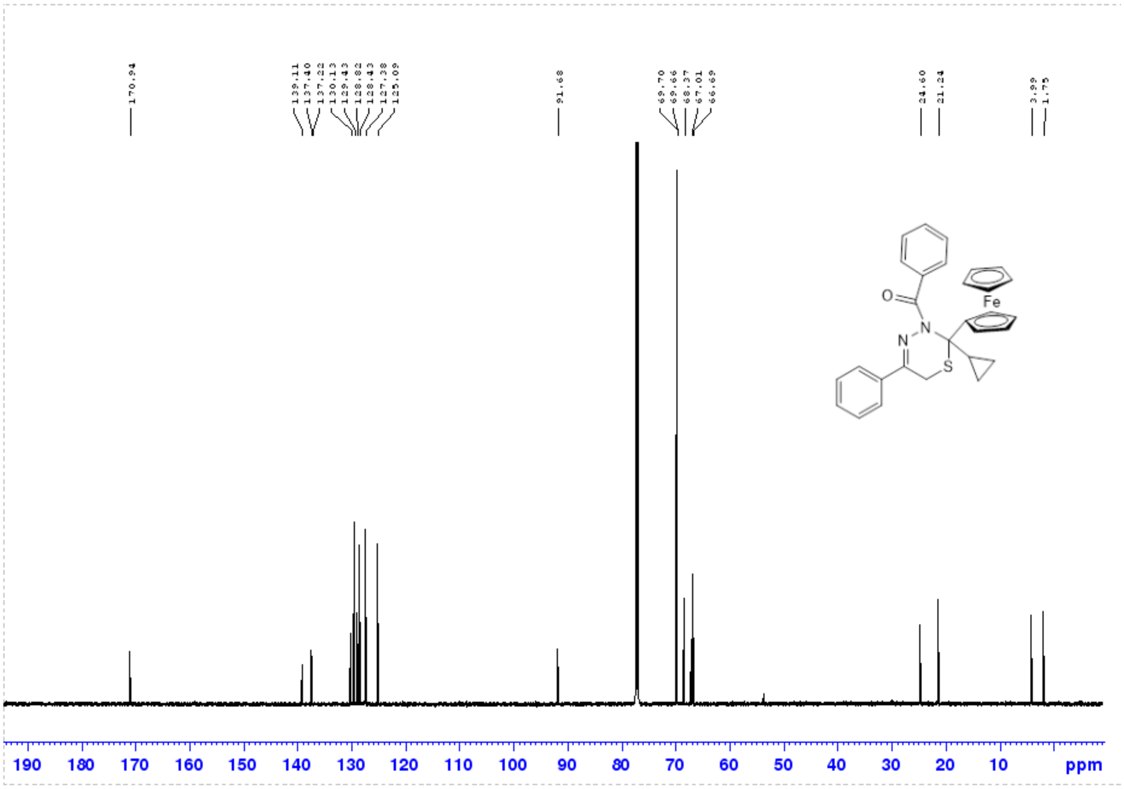

Fig. 16b. ^{13}C-NMR for compound **9p**.

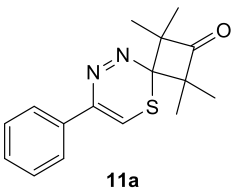

11a

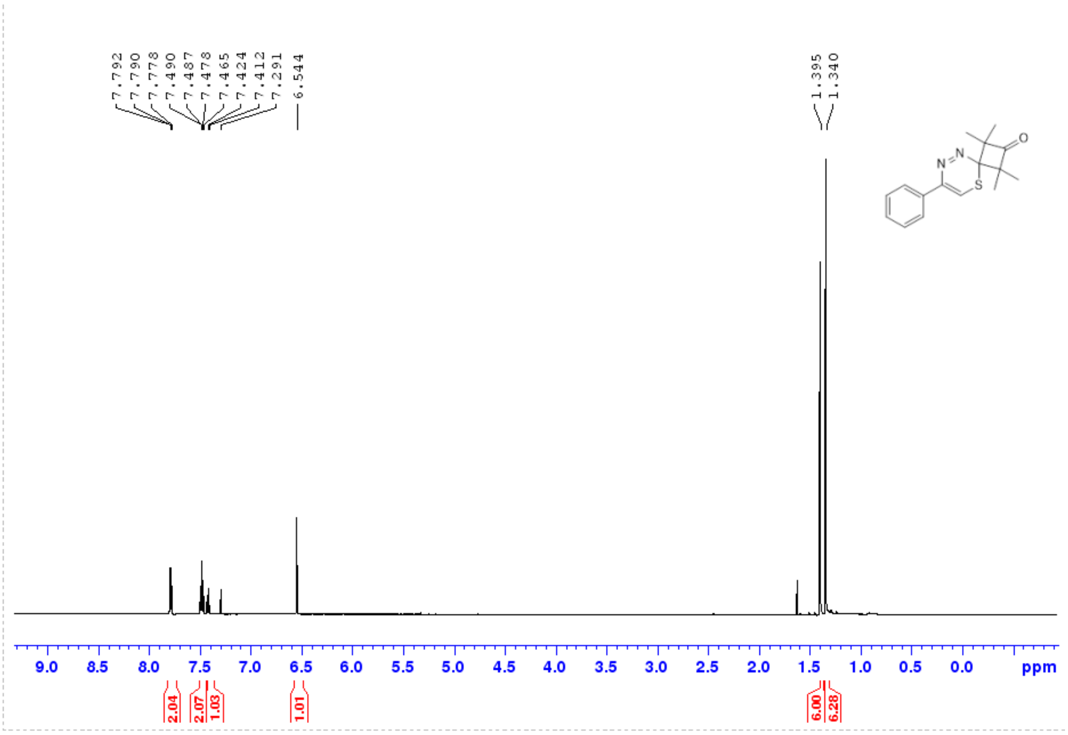

Fig. 17a. ^{1}H-NMR for compound **11a**.

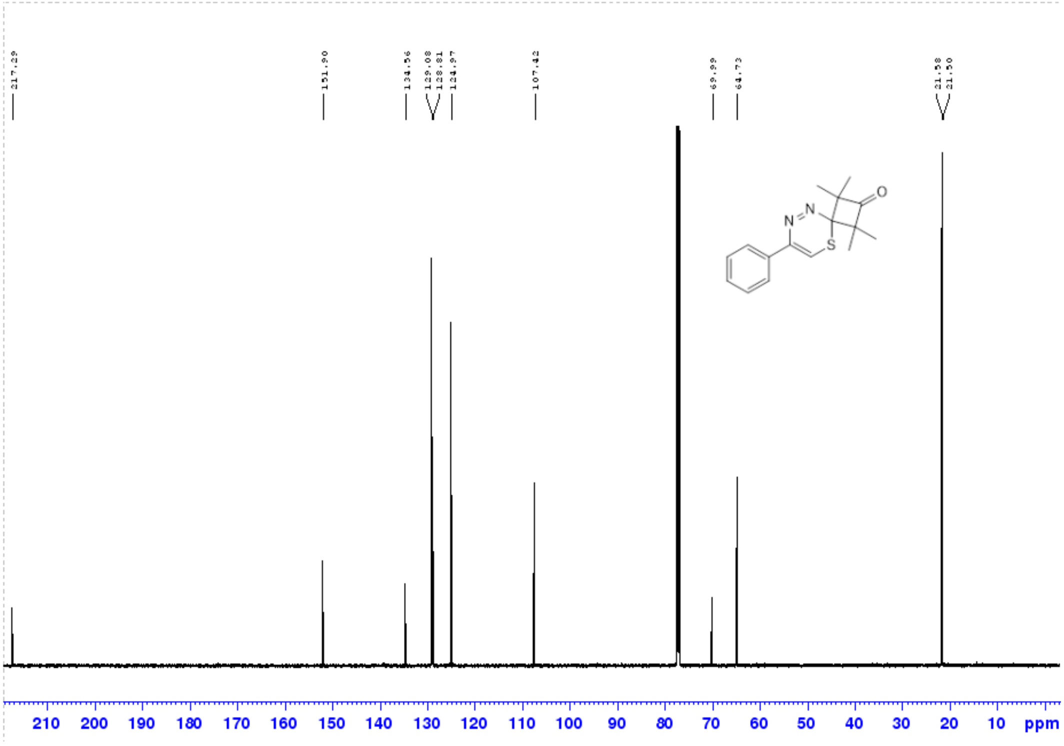

Fig. 17b. ^{13}C-NMR for compound **11a**.

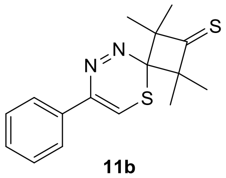

11b

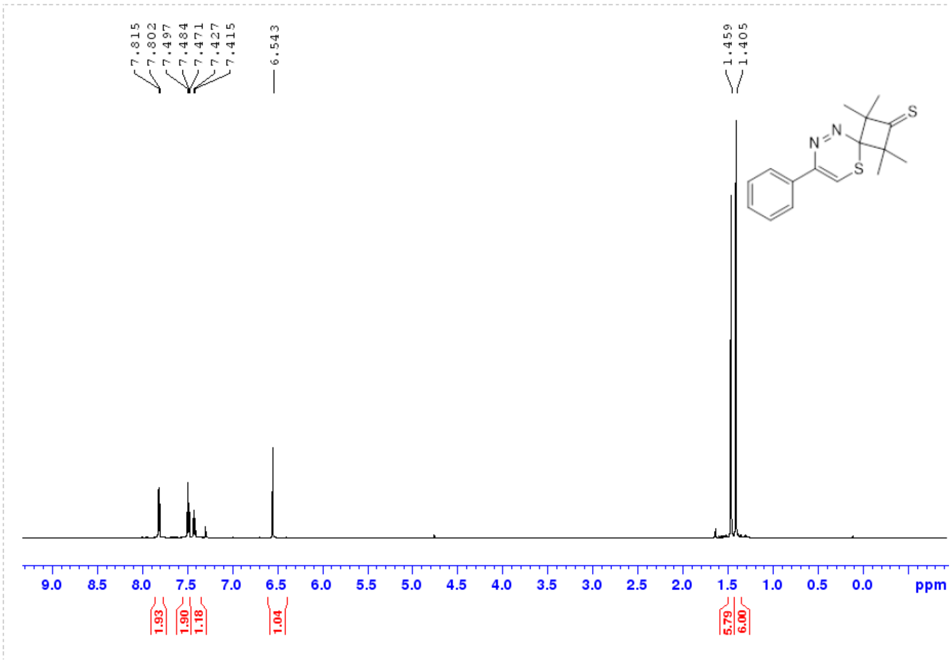

Fig. 18a. ^{1}H-NMR for compound **11b**.

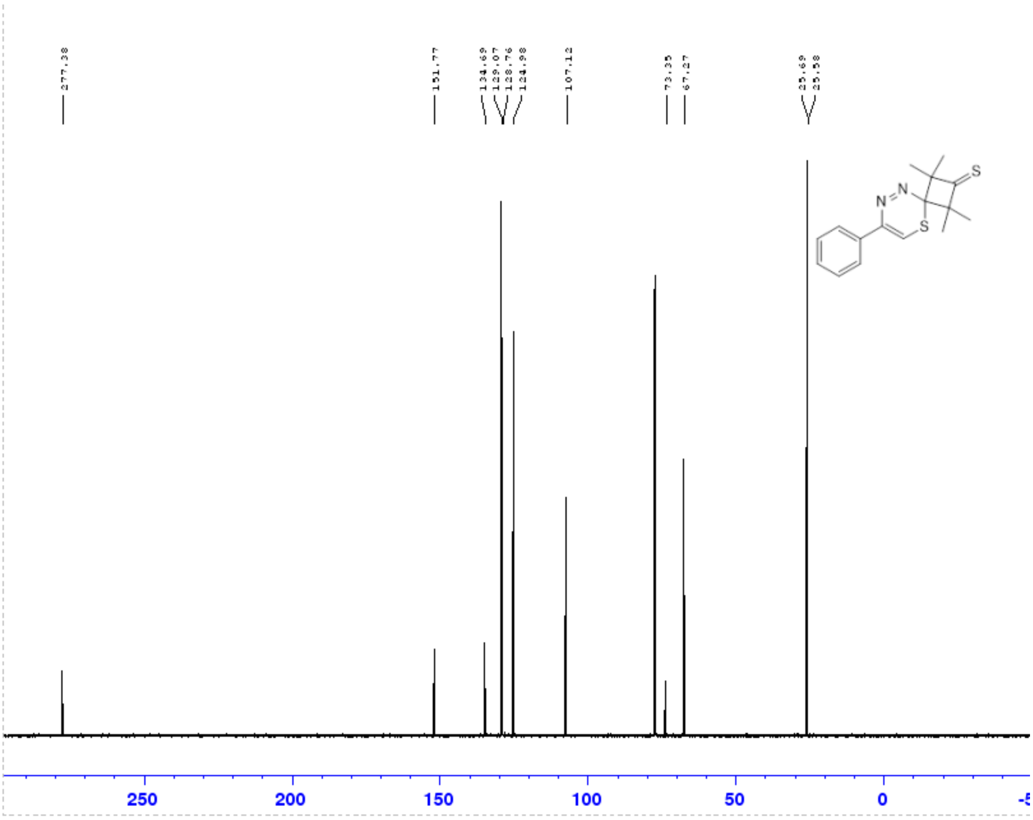

Fig. 18b. ^{13}C-NMR for compound **11b**.

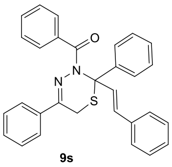

9s

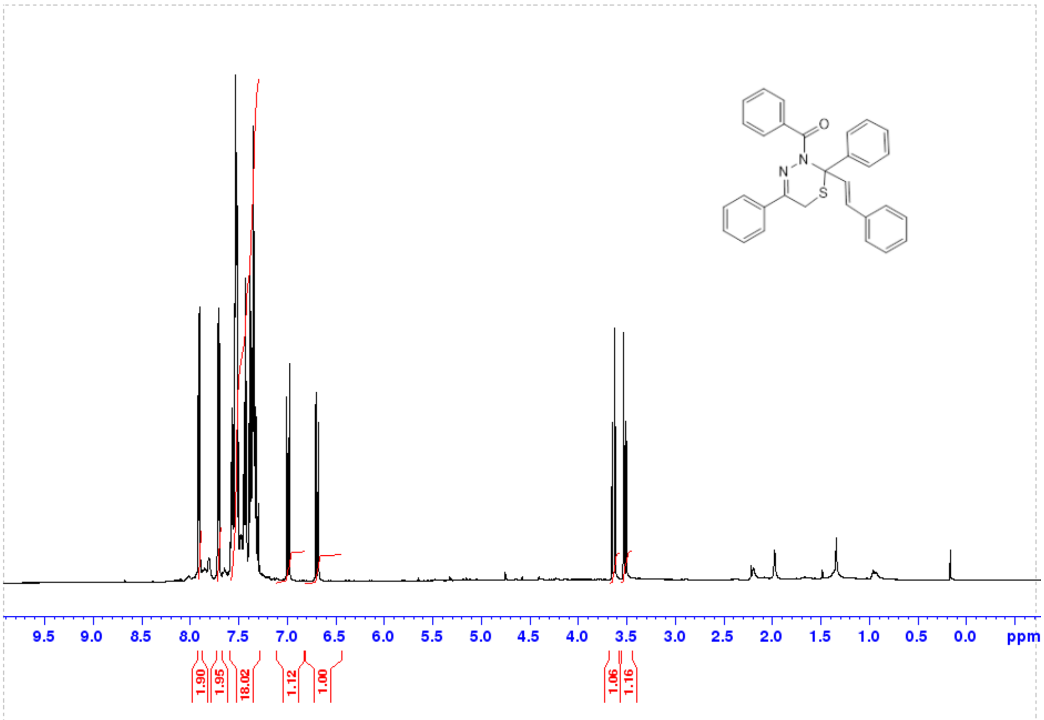

Fig. 19a. ^{1}H-NMR for compound **9s**.

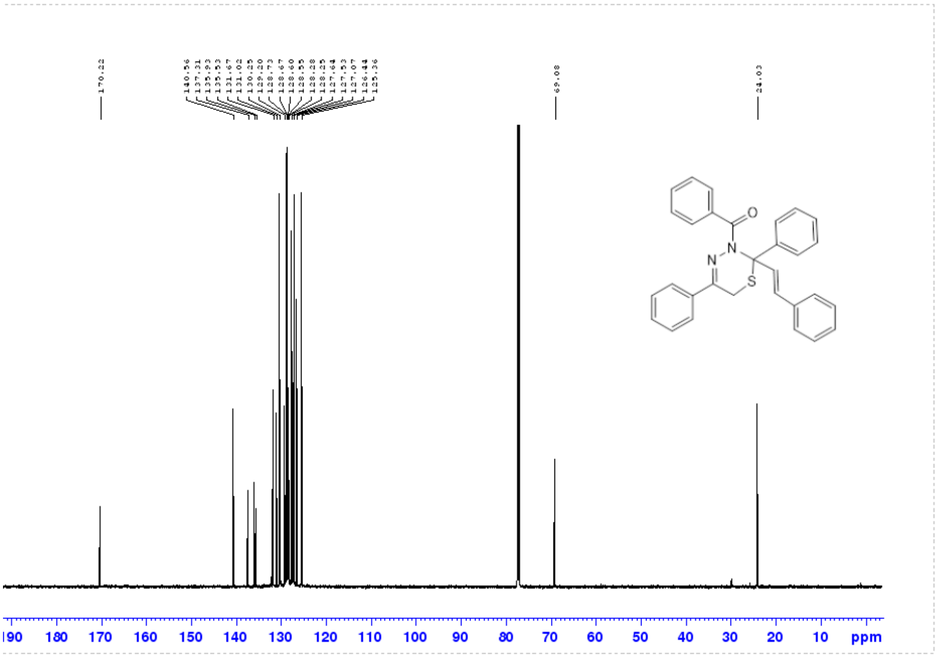

Fig. 19b. ^{13}C-NMR for compound **9s**.

1.2. The UV-Vis spectra for products 11a-11b.

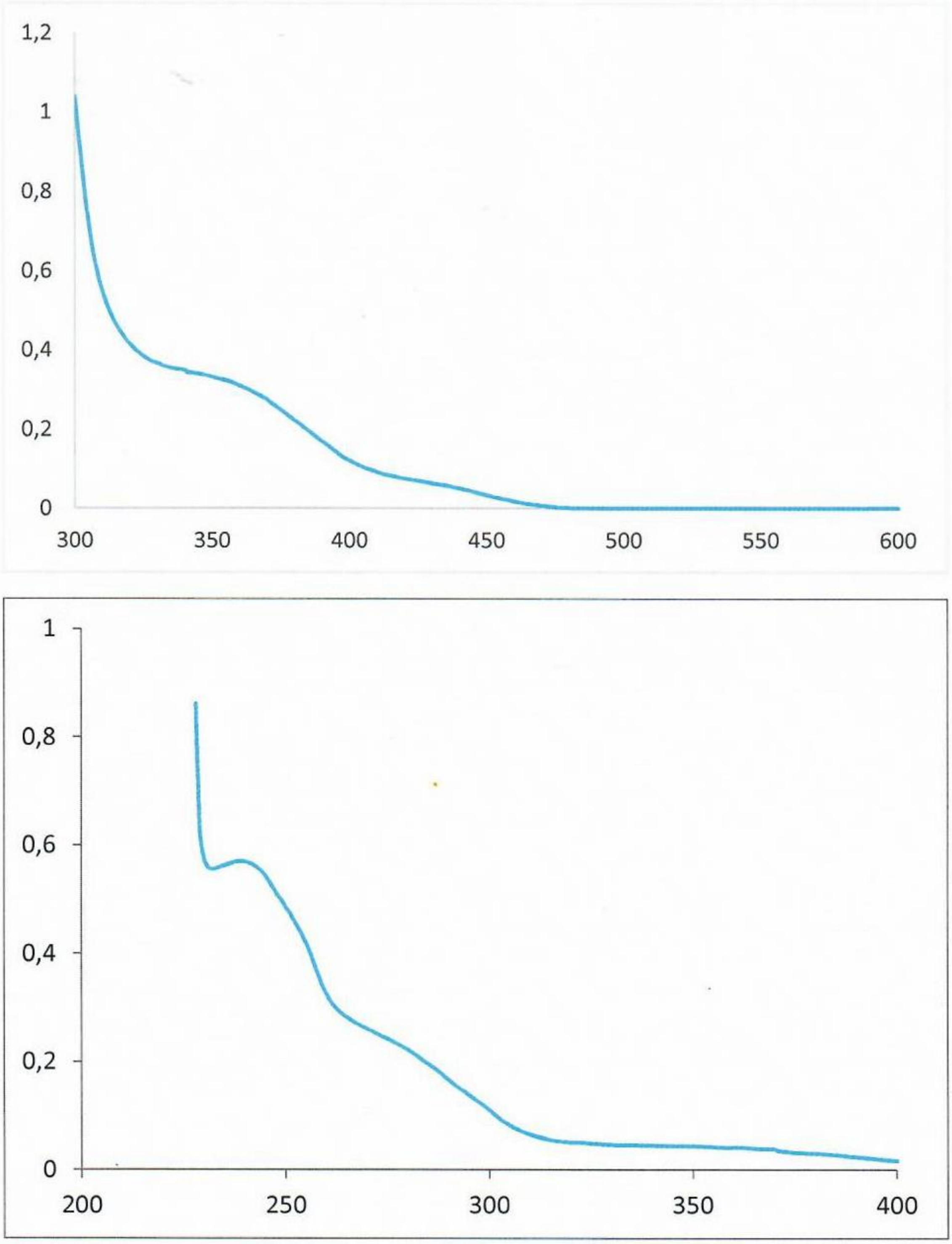

Fig. 20a. UV-Vis registered for **11a (**Concentration: c = 4,400 x 10^{-5} mol/dm^3; λ_1: 239 nm, ε_1 = 1.3 x 10^4; λ_2: 283 nm, ε_2 = 3.7 x 10^3; λ_3: 358 nm, ε_3 =7.4 x 10^2; λ_4: 430 nm, ε_4 = 1.6 x 10^2).

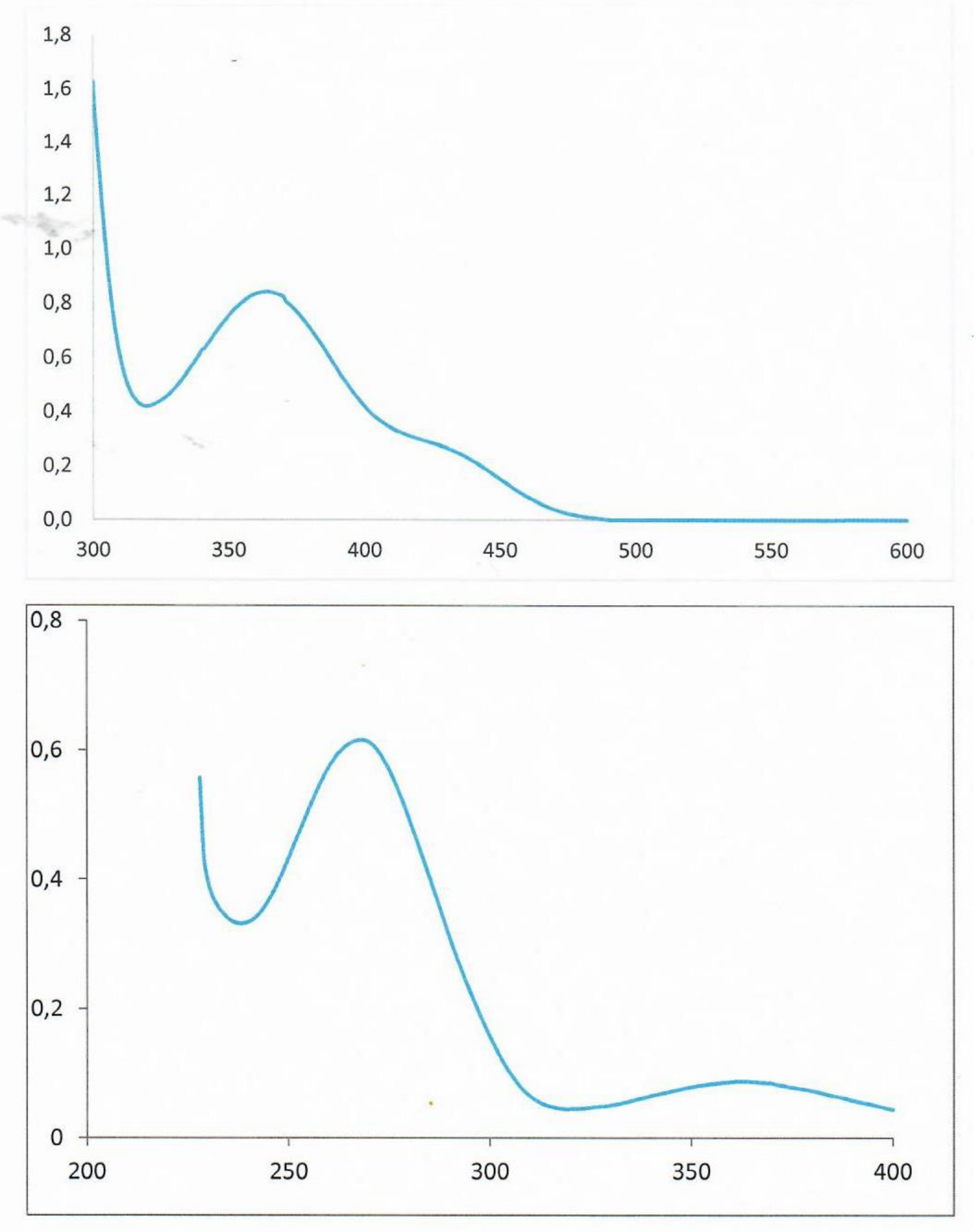

Fig. 20b. UV-Vis registered for **11b** (Concentration: 4,398 x 10^{-4} mol/dm^3; Absorptions: λ_1 268 nm, ε_1 = 1.36 x 10^4; λ_2 364 nm, ε_2 = 1.91 x 10^3; λ_3: 426 nm, ε_3 = 5.8 x 10^2).

2. The X-Ray structure determinations

Table S1. Crystallographic data and structure refinement details for compounds **9c** and **9g**.

Compound	**9c**	**9g**
CCDC number	2072033	2072034
Formula	$C_{28}H_{22}N_2O_2S_2$	$C_{28}H_{22}N_2O_2S_3$
M_r	482.60	514.65
Crystal size (mm)	0.39 x 0.16 x 0.04	0.15 x 0.1 x 0.05
Crystal system	monoclinic	triclinic
Space group	$P2_1/c$	P-1
Temperature (K)	100	100
a (Å)	10.5875(4)	10.2742(3)
b (Å)	15.0374(6)	10.3516(3)
c (Å)	14.4764(5)	11.6550(3)
α (°)	90	81.401(1)
β (°)	91.419(2)	77.990(1)
γ (°)	90	82.594(1)
V (Å^3)	2304.06(15)	1192.66(6)
Z	4	2
D_x (g cm^{-3})	1.391	1.433
λ (Å)	0.71073	0.71073
μ (mm^{-1})	0.26	0.341
Transmissions	0.905–0.990	0.892–0.954
F(000)	1008	536
$2\theta_{max}$	61.0	70.3
Refl. measured	64825	34181
Refl. indep.	7053	11499
R_{int}	0.059	0.0295
Parameters	308	317
wR (F^2, all refl.)	0.1203	0.1071
R(F, >4σ(F))	0.0395	0.0354
S	1.054	1.505
Max. Δp (e Å^{-3})	0.380, –0.425	0.57, –0.43

3. DFT calculations

All calculations were performed using the GAUSSIAN 16, B.01 package of programs [1]. The structures were fully optimized first using the B3LYP/6-31G(d)[2,3]+GD3BJ [4,5] method, then the PBE1PBE/def2tzvp[6–10]+GD3BJ [4,5] functional including the PCM-solvent sphere for dichloromethane [11]. Zero point vibrational energies and free enthalpy contributions were determined analytically.

Table S2: Total energies (E_{tot}) and Gibbs free energies (G_{298}) [a.u.], of thiones (**1**)-azoalkene **7a**-cycloadducts **9** and **10**, respectively, van der Waals complexes and transition states and the relative energies with respect to the corresponding educts (last column) [kcal/mol].

Species	E_{tot} [a.u.]	E_{rel} [kcal/mol]	G_{298} [a.u.]	E_{rel} [kcal/mol]
**1a**	-899,06563		-898,91347	
**7a**	-1.237,34909		-1.237,13653	
**Sum of 1a+7a**	-2.136,41472	0.00	-2.136,05000	0.00
**1a+7a->9a**	-2.136,47789	-39,64	-2.136,08308	-20,76
**vdW from 1a + 7a**	-2.136,42596	-7,05	-2.136,04310	4,33
**TS for 9a**	-2.136,42240	-4,82	-2.136,03690	8,22
**1a+7a->10a**	-2.136,47314	-36,66	-2.136,08074	-19,29
**TS for 10a**	-2.136,40840	3,97	-2.136,01930	19,26
**1b**	-897,88171		-897,74937	
**Sum of 1b + 7a**	-2.135,23079	0.00	-2.134,88589	0.00
**1b+7a->9c**	-2.135,30019	-43,55	-2.134,92418	-24,02
**vdW from 1b + 7a**	-2.135,24502	-8,93	-2.134,88108	3,02
**TS for 9c**	-2.135,23749	-4,20	-2.134,86995	10,00
**1b+7a->10c**	-2.135,29415	-39,76	-2.134,92199	-22,65
**TS for 10c**	-2.135,22766	1,97	-2.134,85793	17,55
**1m**	-975,21672		-975,05214	
**Sum of 1m+7a**	-2.212,56580	0.00	-2.212,18866	0.00
**1m+7a->9m**	-2.212,62248	-35,56	-2.212,21346	-15,56
**vdW from 1m+7a**	-2.212,57931	-15.09	-2.212,18337	3,32
**TS for 9m**	-2.212,56876	-1,85	-2.212,16780	13,09
**1m+7a->10m**	-2.212,62629	-37,96	-2.212,21852	-18,74
**TS for 10m**	-2.212,53590	18,77	-2.212,13515	33,58

Table S2 continued.

Species	E_{tot} [a.u.]	E_{rel} [kcal/mol]	G_{298} [a.u.]	E_{rel} [kcal/mol]
**1n**	-976,43164		-976,24474	
**Sum of 1n+7a**	-2.213,78073	0.00	-2.213,38127	0.00
**1n+7a->9n**	-2.213,84387	-39,62	-2.213,41218	-19,40
**vdW from 1n + 7a**	-2.213,79526	-9,12	-2.213,37501	3,93
**TS for 9n**	-2.213,78692	-3,88	-2.213,36224	11,94
**1n+7a->10n**	-2.213,83438	-33,66	-2.213,40562	-15,28
**TS for 10n**	-2.213,75097	18,68	-2.213,32794	33,46
**1i**	-785,04269		-784,89811	
**Sum of 1i+7a**	-2.022,39178	0.00	-2.022,03464	0.00
**1i+7a->9q**	-2.022,45640	-40,55	-2.022,06501	-19,06
**vdW from 1i + 7a**	-2.022,40293	-7,00	-2.022,02787	4,25
**TS1-stepwise**	-2.022,39264	-0,54	-2.022,01364	13,17
**Z (Intermediate)**	-2.022,40302	-7,05	-2.022,01945	9,53
**TS2-stepwise**	-2.022,40177	-6,27	-2.022,01547	12,03
**1i+7a->10q**	-2.022,46135	-43,65	-2.022,07099	-22,81
**TS for 10q**	-2.022,37772	8,82	-2.021,99340	25,88
**11a**	-1.202,66061		-1.202,39925	
***p*-tolylsulfinic acid 12**	-819,76099		-819,66041	
**TS for 1i+7a (NNC)**[a)]	-2.022,36888	14,37	-2.021,98357	32,05
**1i+7a (NNC)** [a)]	-2.022,43486	-27,03	-2.022,04115	-4,09
**TS for 1i+7a (NNO)** [a)]	-2.022,35652	22,13	-2.021,97281	38,80
**1i+7a (NNO)** [a)]	-2.022,40164	-6,19	-2.022,01265	13,80

[a)] Addition to C=O bond of **1i**.

3.1. Gaussian Archive Entries

(Total energies (a.u.), number of imaginary frequencies (for transition states: imaginary frequencies), coordinates)

1a

HF=-899.0656303 a.u. (0)

1\1\GINC-R08N09\FOpt\RPBE1PBE\def2TZVP\C13H10S1\WURTHWE\ 13-Feb-2021\0\\# pbe1pbe/def2tzvp emp=gd3bj Opt nosym Pop=NBO Freq scrf=(solvent=dichloromethane)\\ 1a Thiobenzophenone\\0,1\C,0.,-0.0000000004,0.042711794\S,0.0000000001,0.,1.6837354658\C,1.2557467119, 0.0884042861,-0.7175425213\C,-1.2557467119,-0.0884042865,-0.7175425212\ C,1.4057634606,-0.6117728542,-1.9191088769\C,2.6078212372,-0.5791812938,-2.6060403986\C,3.6661419372,0.1755513656,-2.1205121721\C,3.52345991, 0.887239145,-0.9349427787\C,2.3337451933,0.8341792594,-0.2331937183\ H,0.584192158,-1.2067951338,-2.2982731448\H,2.7176554922,-1.1442445435,-3.5240433945\H,4.6022675673,0.2113620108,-2.6655457571\H,4.3448251108, 1.4852857373,-0.5582433018\H,2.2144083461,1.380041514,0.6947214337\C,-1.4057634604,0.6117728538,-1.9191088769\C,-2.607821237,0.5791812939,-2.6060403985\C,-3.6661419373,-0.1755513652,-2.120512172\C,-3.5234599102,-0.8872391447,-0.9349427786\C,-2.3337451935,-0.8341792595,-0.2331937182\H,-0.5841921576,1.2067951332,-2.2982731448\H,-2.7176554919,1.1442445436,-3.5240433944\H,-4.6022675674,-0.2113620102,-2.665545757\H,-4.3448251112,-1.4852857368,-0.5582433017\H,-2.2144083464,-1.3800415141,0.6947214338\\ Version=ES64L-G16RevB.01\HF=-899.0656303\RMSD=3.877e-09\RMSF= 9.391e-06\Dipole=0.,0.,-1.805389\Quadrupole=6.8796793,-5.3016639,-1.5780154,3.8736704,0.,0.\PG =C02 [C2(C1S1), X(C12H10)]\\@

7a

HF=-1237.349088 a.u. (0)

1\1\GINC-R08N05\FOpt\RPBE1PBE\def2TZVP\C15H14N2O2S1\ WURTHWE\ 05-Mar-202 0\0\\# pbe1pbe/ def2tzvp Opt=readfc geom=check guess=read scrf=(solvent=dichloromethane) freq pop=nbo emp=gd3bj nosym\\7a Tosylazoalkene\\0,1\C,-0.1476006043,-0.0571155243,-0.140160722\C,0.0058651256,0.0348141764,1.2433905715\C,1.2903450417, 0.1704767518,1.7736813537\C,2.3946036608,0.1899228894,0.9360954454\ C,2.2344194585,0.0831316289,-0.4376440746\C,0.9581989886,-0.0379698151,-0.9726044737\C,-1.1713079966,-0.0027030352,2.1259390395\C,-2.3290009924,-0.6173703708,1.8738755755\N,-0.9326588434,0.5781080728,3.39817785\N,-1.8763591229,1.1534628411,3.9238461006\S,-1.5175406729,1.7665045356,

5.5144761968\O,-1.7455286349,3.1769147077,5.397137409\C,0.1727601006,
1.4461813991,5.8026006615\C,0.550605182,0.2450962506,6.3872807765\
C,1.8968915908,-0.0281548254,6.5462142653\C,2.8699991064,0.8766356417,
6.123137358\C,2.4608462098,2.0730848471,5.5340873599\C,1.11987051,
2.3646386166,5.3682783225\C,4.3225941321,0.5824656765,6.319103989\O,-
2.3364850737,0.9725299396,6.3840635662\H,1.4199093838,0.2523311994,2.84533
08306\H,3.3864790545,0.2891634535,1.3617671833\H,3.0988217918,
0.102532506,-1.0909830763\H,0.8227729827,-0.1071200872,-2.0456231076\H,-
1.1407886424,-0.124755364,-0.5684664586\H,-2.4796720777,-1.1763623556,
0.9603094979\H,-3.1246891805,-0.6086416184,2.6074294262\H,2.2002322584,-
0.9633375719,7.0032409125\H,-0.2030087941,-0.4609971385,6.7119945392\
H,0.805168379,3.2923235363,4.907458842\H,3.2063160545,2.7848796295,5.19770
7859\H,4.9287363952,1.0389702333,5.5354477513\H,4.5124988041,-
0.4914138612,6.3305353555\H,4.6655132597,0.9875965776,7.2760925324\\
Version=ES64L-G16RevB.01\HF=-1237.349088\RMSD=3.701e-09\RMSF=
2.169e-06\Dipole=2.7504301,-1.3458846,-1.2804855\Quadrupole=11.2036248,-
3.5918655,-7.6117594,11.4512086,32.9203444,-14.0202401\PG=C01
[X(C15H14N2O2S1)]\\@

9a (from 1a and 7a)

HF=-2136.4778897 a.u (0)

1\1\GINC-R09N15\FOpt\RPBE1PBE\def2TZVP\C28H24N2O2S2\
WURTHWE\13-Feb-202 0\0\\# pbe1pbe/def2tzvp Opt=readfc geom=check
guess=read Pop=NBO Freq emp=gd3bj nosym scrf=(solvent=dichloromethane)
\\9a NNC-Product\\0,1\C,-1.9393748698,-0.66373739,-0.3385230461\ C,-
3.265097497,-0.5850208377,0.0467489798\C,-3.8491899784,-1.6221973124,
0.7651069396\C,-3.0955282801,-2.7400645105,1.0878672316\C,-1.7671840576,-
2.8233669853,0.6963925644\C,-1.1699284446,-1.7829625394,-0.0145458799\
C,0.2523818672,-1.8587626745,-0.3999628631\C,0.9044077691,-3.2020134134,-
0.501315892\N,0.8323833255,-0.746806339,-0.6489826444\N,2.1306794622,-
0.6215334171,-0.9800928\S,2.5068029001,1.017009308,-1.1491073841\
O,3.9342355659,1.1137694641,-1.1173613247\O,1.7758310119,1.5166891823,-
2.2750328959\C,0.8752415972,3.0008958568,2.5875873701\C,0.057535288,
2.8096109176,1.472005933\C,0.5396678358,2.2032589817,0.3291925427\
C,1.860399422,1.7737559433,0.2956405607\C,2.7001660145,1.968222836,
1.3826255942\C,2.2005908055,2.5803342037,2.5205990199\C,0.3297146293,
3.6410043217,3.8240806891\C,3.0953779483,-1.730303626,-1.0594371439\
S,2.1727901067,-3.16083592,-1.7660946121\C,4.4835591164,-2.9368336404,
2.8434389339\C,3.6488738716,-1.8408717244,2.7133044089\C,3.2070742468,-
1.4429736998,1.4569713584\C,3.5958458637,-2.13122503,0.3170001125\
C,4.416482884,-3.2509689683,0.459619566\C,4.8640276186,-3.6436363244,
1.7080187046\C,5.5279854526,-1.4398862423,-1.7779639617\C,6.4769820942,-

1.1907547755,-2.7584834433\C,6.0868716164,-0.9441396386,-4.0656477469\C,4.7366267676,-0.9442373658,-4.3874288611\C,3.7894076308,-1.1978975954,-3.4101828723\C,4.1769846632,-1.4497726107,-2.0954628271\H,-1.4831682629,0.136000706,-0.9071610259\H,-3.8509199515,0.2870334392,-0.2205195177\H,-4.8886644175,-1.5599647693,1.0652989278\H,-3.5408778629,-3.5540195267,1.6480098118\H,-1.1918749806,-3.7011700137,0.966221099\H,1.3284727314,-3.5274638288,0.4535640719\H,0.1783648933,-3.9505699097,-0.8196601291\H,-0.9735220723,3.1445363857,1.5021026965\H,-0.0922290347,2.0656481204,-0.537167752\H,3.7339645452,1.651564753,1.3320821231\H,2.8556693323,2.7384228965,3.3699557982\H,1.1221217528,3.8748842323,4.535044366\H,-0.2058663297,4.5623130106,3.5850195501\H,-0.3824068502,2.9741124932,4.3184622288\H,4.8300665034,-3.2486204394,3.8217650479\H,3.3297767815,-1.287907387,3.589189775\H,2.5413596456,-0.5948510073,1.3798489714\H,4.6995217913,-3.8212712592,-0.4167974332\H,5.508594375,-4.5103536015,1.7970046074\H,5.8477159892,-1.6024976718,-0.7577503866\H,7.5277349446,-1.1814359691,-2.492905619\H,6.8300132289,-0.7472179419,-4.8295514797\H,4.417386853,-0.7445276647,-5.403620195\H,2.7361141473,-1.1852854812,-3.6644208476\\Version=ES64L-G16RevB.01\HF=-2136.4778897\RMSD=3.516e-09\RMSF=1.275e-06\Dipole=-0.9525195,-0.808447,2.7391283\Quadrupole=0.1676077,0.8836617,-1.0512694,-8.5167297,7.4089125,4.6575909\PG=C01 [X(C28H24N2O2S2)]\\@

1a + 7a vdW-complex

HF=-2136.425961 a.u.(0)

1\1\GINC-R10N37\FOpt\RPBE1PBE\def2TZVP\C28H24N2O2S2\WURTHWE\13-Feb-202 0\0\\# pbe1pbe/def2tzvp opt=readfc geom=check guess=read scrf=(solvent =dichloromethane) freq pop=nbo emp=gd3bj nosym\\1a + 7a vdW-complex\\0,1\C,0.231977665,2.8374631356,-3.6161122983\C,0.3713738998,2.7905928972,-2.2260658986\C,1.5139236086,2.1955615487,-1.6797198677\C,2.476884226,1.6406438273,-2.5070635672\C,2.3310879494,1.7004275543,-3.8853931996\C,1.2092731201,2.3086340653,-4.4380254797\C,-0.6998138999,3.2760372551,-1.3431792511\S,-2.2768187417,3.1227705423,-1.7727623291\C,-0.311054726,3.8730585795,-0.0597117433\C,0.8521206258,4.6437465527,0.0386720317\C,1.1983903927,5.2414967632,1.2381543542\C,0.4056181348,5.0545390746,2.3619627072\C,-0.744760389,4.2797441474,2.2784031373\C,-1.1067881531,3.7038658532,1.0756114689\C,-1.0803815665,-0.5390347932,-2.2323240295\C,-1.1524104295,-1.401737153,-1.2164265796\C,-1.786577985,-2.730045572,-1.2502418325\C,-1.3299701704,-3.7608807224,-0.426743208\C,-1.9227587005,-5.0127477758,-0.4787023613\C,-2.9812527693,-5.2519717732,-1.3429404867\C,-3.4497977036,-4.2266047408,-2.1543976253\C,-2.8612424813,-2.9744877158,-2.105333252\N,-0.4692485738,-1.1458492899,-0.0012071201\N,-0.4522451699,0.0125342243,0.3951013091\

S,0.4396410385,0.212510696,1.880992534\O,-0.555564005,0.628304143,
2.8251283633\C,1.0532546889,-1.365104168,2.3058678819\ C,0.2606986647,-
2.2213826695,3.0589085504\C,0.7230742755,-3.4949325017,3.3330664825\
C,1.9628518652,-3.9274967407,2.8623460743\C,2.7350906564,-3.0474859648,
2.1047959564\C,2.2887187982,-1.7700448087,1.8194549913\C,2.4634974104,-
5.2986925934,3.1851416816\O,1.5215057571,1.0897741162,1.5364664978\H,-
0.5037722693,-3.5784864543,0.2485901797\H,-1.5537177809,-5.8058300186,
0.1614627398\H,-3.4461402115,-6.2302622775,-1.3789518182\H,-4.2861591434,-
4.4000892787,-2.8214701344\H,-3.250896548,-2.172813227,-2.7217905753\H,-
0.5642458713,0.4042986173,-2.1207516043\H,-1.4906766859,-0.784125672,-
3.2026991776\H,0.1097649322,-4.1694837196,3.9199182298\H,-0.7038924282,-
1.8895293112,3.4213279086\H,2.888179733,-1.0901406368,1.2274379065\
H,3.6994683874,-3.3712117032,1.7296978773\H,2.9942775772,-5.2928918355,
4.1420974184\H,1.6414548859,-6.0104588078,3.2725997348\H,3.159634389,-
5.656471047,2.4257216756\H,3.0905415729,1.2750163777,-4.5311651303\
H,3.343899246,1.1582427871,-2.0711847523\H,1.6211161627,2.1269892429,-
0.6047586536\H,-0.6525084805,3.3006909583,-4.0359738188\H,1.0978037596,
2.3668028837,-5.5143237634\H,1.4713190523,4.792810281,-0.8372784447\
H,2.0919315396,5.8516208314,1.2971048211\H,0.6866118997,5.50837211,
3.3052953201\H,-1.3554479362,4.1165490413,3.1582675908\H,-1.995699095,
3.0900958658,1.0019425784\\Version=ES64L-G16RevB.01\HF=-2136.425961\
RMSD=8.518e-09\RMSF=1.117e-06\Dipole=1.84845,-3.038735,-0.2895545\
Quadrupole=-6.2283789,6.2379413,-0.0095625,6.759272,0.34714,-
7.5968848\PG=C01[X(C28H24N2O2S2)]\\@

TS for 9a

HF=-2136.4224007 a.u. (1, -86,8511cm^{-1})

1\1\GINC-R08N29\FTS\RPBE1PBE\def2TZVP\C28H24N2O2S2\
WURTHWE\14-Feb-2020\0\\# pbe1pbe/def2tzvp Opt=(ts,noeigentest,
readfc) geom=check guess=read freq pop=nbo emp=gd3bj nosym
scrf=(solvent=dichloromethane)\\TS for 9a \\0,1\C,0.4014402519,-
0.3765159971,0.153198678\C,0.0820383761,-0.1323584563,1.4928766126\
C,1.1074231569,-0.1502842009,2.4450745452\C,2.412226576,-0.4111369304,
2.0631990684\C,2.7178083952,-0.6418990895,0.7289880033\C,1.7082802645,-
0.6206620807,-0.225991715\C,-1.308429988,0.0812730062,1.9054350678\S,-
2.5432151983,-0.6554759652,1.1043000927\C,-1.588125835,0.9628682728,
3.0444652765\C,-0.8046432207,2.0987699036,3.2684795507\C,-1.0927938168,
2.9547219852,4.3186393551\C,-2.1500038705,2.677016075,5.1726660518\C,-
2.9304201049,1.5452106535,4.9635822376\C,-2.6604570029,0.7036436897,
3.9027964747\C,-1.8690591672,-3.1381365419,1.2978242742\C,-2.6000780819,-
3.4522197224,2.4003877347\C,-3.9560627879,-4.0197211218,2.3675242982\C,-
4.8076894435,-3.8689205257,3.464229865\C,-6.0966969517,-4.3758823226,

3.4340630426\C,-6.5615332082,-5.0421043853,2.3090026374\C,-5.7220464309,-5.2013573146,1.2144050377\C,-4.4323033879,-4.6983500268,1.2438712894\N,-2.1074907514,-3.210003876,3.6795645723\N,-1.0821602085,-2.5120270336,3.7648517491\S,-0.5590776705,-2.3541343092,5.4035917784\O,-1.593153229,-2.7212856408,6.3293923991\C,0.7126896343,-3.5544033067,5.4328162435\C,0.4326035781,-4.8380777498,5.8854465334\C,1.4337617564,-5.7907467086,5.8611425071\C,2.7096079863,-5.4846025728,5.3855588326\C,2.9601350403,-4.1913697455,4.9300231839\C,1.9716180365,-3.2236607703,4.9493211245\C,3.7891288666,-6.5192416394,5.3880147719\O,0.0220608013,-1.0420225644,5.4636284338\H,-4.4497739742,-3.345938642,4.3421900385\H,-6.7435963117,-4.2445197135,4.2939032467\H,-7.5699974071,-5.4382654156,2.2855968607\H,-6.070560147,-5.7295322477,0.3343584342\H,-3.784986101,-4.8544519291,0.389069382\H,-0.835471892,-2.8350984577,1.4009917362\H,-2.2240385006,-3.3696096318,0.304136752\H,1.2237891408,-6.7934071433,6.2168388197\H,-0.5567068376,-5.077298259,6.2544618364\H,2.1693068529,-2.2194285376,4.5976062883\H,3.9453991064,-3.9396052852,4.5537875674\H,4.587952727,-6.2626955669,4.691660785\H,4.2319199443,-6.6047231589,6.3849980118\H,3.3952066392,-7.5020188239,5.1234668952\H,3.7416480984,-0.8375521304,0.4319252961\H,3.195983165,-0.4364129964,2.8115033904\H,0.8644952541,-0.0054736002,3.4895478011\H,-0.3909374987,-0.3555044728,-0.5852018474\H,1.9434411166,-0.7910949892,-1.2699326451\H,0.0179139446,2.3223626832,2.6005228976\H,-0.4874474127,3.8404209424,4.4711545989\H,-2.3653018857,3.3389142532,6.0034161347\H,-3.7482940248,1.3170523657,5.6367248883\H,-3.2597060306,-0.1833590661,3.7373191449\\Version=ES64L-G16RevB.01\HF=-2136.4224007\RMSD=8.341e-09\RMSF=1.903e-06\Dipole=2.2151311,-1.1203046,-1.4114871\Quadrupole=7.0313603,16.9801704,-24.0115307,-10.4322825,15.4841978,-0.2366612\PG=C01[X(C28H24N2O2S2)]\\@

10a (from 1a and 7a)

HF=-2136.4731387 a.u. (0)

1\1\GINC-R09N28\FOpt\RPBE1PBE\def2TZVP\C28H24N2O2S2\WURTHWE\12-Feb-2020\0\\# pbe1pbe/def2tzvp Opt=readfc geom=check guess=read Pop=NBO Freq emp=gd3bj nosym scrf=(solvent=dichloromethane)\\10a NNS-Product\\0,1\C,-1.6028265051,-0.8445503227,0.1150750005\C,-2.9599604395,-1.0366581732,0.2934129849\C,-3.4224043498,-1.9347962833,1.2491234715\C,-2.5126400937,-2.6398785973,2.0206525093\C,-1.1496288293,-2.4541257312,1.8380945865\C,-0.6760925573,-1.5514456476,0.8863904584\C,0.772216558,-1.3337366084,0.7003181595\C,1.7250558682,-2.3455920297,1.2761410517\N,1.0991906228,-0.2803996757,0.0527882428\N,2.3875819026,0.0166067494,-0.2047809521\S,2.5868475613,1.5394147923,-0.880750471\O,3.9950222092,1.6474864588,-1.1160697678\O,1.6526859716,1.6405192781,-1.9567705939\C,1.3510149866,4.2936681222,2.4897328008\C,0.405685761,3.800

719061,1.5902463367\C,0.7761084551,2.979492919,0.5414973554\C,2.1159063065,2.6440066458,0.3892106727\C,3.0777154625,3.1144282084,1.2734194715\C,2.687092034,3.938147981,2.3145067934\C,0.940670949,5.2052012265,3.6021058202\S,3.6904840596,-0.6312325005,0.734299278\C,3.1007692911,-2.3507393451,0.616619958\C,2.9225262734,-3.9482751365,-3.3986117503\C,3.5585404565,-2.738607499,-3.1711802014\C,3.6142331765,-2.2040287685,-1.8913213937\C,3.0317734809,-2.8679910213,-0.8187917316\C,2.4036763677,-4.0902760832,-1.0556586487\C,2.3482425965,-4.625655402,-2.3319428433\C,4.1402571848,-3.1389918131,1.4021122251\C,3.8283353374,-3.8569405289,2.551028156\C,4.8103497063,-4.5752907135,3.2208623039\C,6.1161579704,-4.5768483582,2.7578374158\C,6.4364825806,-3.8618401662,1.6111523113\C,5.4555259128,-3.1554415426,0.9368812062\H,-1.2394016491,-0.1489262957,-0.6307393063\H,-3.6645497572,-0.4866477298,-0.319797156\H,-4.4868470081,-2.084712965,1.387 0769956\H,-2.8614804669,-3.3405670276,2.7702773811\H,-0.4581428893,-3.0111430358,2.4581984222\H,1.8362110699,-2.1493234394,2.3450253734\H,1.2662829775,-3.3316036144,1.1861509345\H,-0.6385587311,4.0651344388,1.7150975153\H,0.0407662309,2.5990310763,-0.1551129841\H,4.1175551782,2.8441573847,1.1395439558\H,3.4341231406,4.3126344165,3.0053636721\H,1.6611760807,5.1831357485,4.4203915424\H,0.8772301,6.2375355114,3.2447386528\H,-0.041895283,4.9356290157,3.992619841\H,2.8765137788,-4.3636148326,-4.3985346411\H,4.0156453099,-2.1997081777,-3.9930240209\H,4.117883621,-1.25921397,-1.7303742636\H,1.9671177227,-4.6486925756,-0.2359991225\H,1.8559838685,-5.5780570517,-2.4913222963\H,2.8168260296,-3.875802194,2.934918128\H,4.5475816546,-5.1344648749,4.1112959989\H,6.8812939869,-5.1350078291,3.2845165339\H,7.4532339064,-3.8594514619,1.2361312796\H,5.7075088604,-2.6154100113,0.0311713481\\Version=ES64L-G16RevB.01\HF=-2136.4731387\RMSD=1.876e-09\RMSF=7.665e-07\Dipole=-0.8453885,-0.9723161,2.7634561\Quadrupole=-6.9501085,10.4827683,-3.5326597,-8.3860117,9.6868797,5.2668306\PG=C01[X(C28H24N2O2S2)]\\@

TS for 10a

HF=-2136.408398 a.u. (1, -268,3840 cm^{-1})

1\1\GINC-R09N23\FTS\RPBE1PBE\def2TZVP\C28H24N2O2S2\WURTHWE\13-Feb-2020 \0\\# pbe1pbe/def2tzvp Opt=(ts,noeigentest,readfc) geom=check guess=read freq pop=nbo emp=gd3bj nosym scrf=(solvent=dichloromethane)\\ TS for 10a \\0,1\C,-0.104165093,0.0161225207,-0.0750968794\C,0.0350983503,0.0177177445,1.3200748696\C,1.3232425917,0.0022317148,1.8564376642\C,2.4349704784,-0.0565648849,1.0285501362\C,2.2803791191,-0.0860966311,-0.3467754411\C,1.0028922503,-0.0392410249,-0.8962743302\C,-1.1447789652,0.0984138394,2.1950567041\S,-0.9795510396,0.4665289573,3.819345598\C,-2.4224393048,-0.5403963337,

1.7901325836\C,-2.4037039406,-1.6709238989,0.9698450126\C,-3.5794745777,-2.3292433932,0.6411513493\C,-4.7959662721,-1.8708762705,1.1201140314\C,-4.8257519818,-0.7549089082,1.9489700237\C,-3.6541935805,-0.1049750703,2.2869528312\N,-0.2752970051,2.4982203724,3.7551651295\S,0.8834989752,2.4834308543,5.0512151265\O,2.1137028478,1.8701532732,4.6413788863\N,0.2717109908,2.9130820403,2.6775820531\C,-0.5129725931,2.9207025496,1.5656832128\C,0.1712175075,3.3233076324,0.3259833977\C,1.564284926,3.3322679538,0.2578392084\C,2.208976796,3.6890671187,-0.9143327949\C,1.4747048554,4.0369531662,-2.0396871311\C,0.0874697973,4.0391647986,-1.979660402\C,-0.5583837557,3.69135914,-0.8050648794\C,-1.7889010919,2.4313778618,1.5989060705\C,1.1374615975,4.2000394601,5.26571042\C,2.2055790953,4.8185970726,4.6301446511\C,2.3813867945,6.180550078,4.7900095194\C,1.5051843394,6.9336900524,5.5716775197\C,0.4385913122,6.2860231124,6.1934596594\C,0.246407452,4.9240898631,6.0460946195\C,1.7219481359,8.4012128817,5.7593627603\O,0.1515213238,1.9626999758,6.1695431992\H,2.1324177845,3.0301258455,1.1271944811\H,3.2922122604,3.6812954108,-0.9541910231\H,1.9803578099,4.3088528657,-2.9590627728\H,-0.4944402876,4.3220816656,-2.8491810366\H,-1.640738727,3.7215160501,-0.7646602584\H,-2.3145738737,2.3720903677,2.5383971641\H,-2.3588483078,2.2897177639,0.6913773673\H,3.2131829025,6.6716015807,4.2973222167\H,2.884209341,4.2347749808,4.0223043621\H,-0.5803821675,4.422634675,6.5329630184\H,-0.2518411879,6.8585987185,6.8025697965\H,0.7860976061,8.9162674473,5.9792168244\H,2.4016594148,8.5803289487,6.5981234745\H,2.1714838361,8.8519304215,4.873434619\H,3.1489358187,-0.1268862202,-0.9936297121\H,3.4257605447,-0.0741389355,1.4671786668\H,1.449007066,0.0230204531,2.9315472452\H,-1.0920603076,0.0878956206,-0.5133051837\H,0.8739433979,-0.0278632162,-1.9721245958\H,-3.6882486736,0.7518827091,2.9478761137\H,-5.7700473745,-0.3895566439,2.335542257\H,-5.7151851336,-2.3813640191,0.8580651671\H,-3.5378930995,-3.2098967673,0.0107793024\H,-1.4593987928,-2.0544130242,0.6062236992\\Version=ES64L-G16RevB.01\HF=-2136.408398\RMSD=3.742e-09\RMSF=9.475e-07\Dipole=-0.5932683,1.7392088,-2.3245434\Quadrupole=4.8773489,22.8564334,-27.7337823,6.9889022,-4.3496174,17.4276774\PG=C01 [X(C28H24N2O2S2)]\\@

1b

HF=-897.8817061 a.u. (0)

1\1\GINC-R08N08\FOpt\RPBE1PBE\def2TZVP\C13H8S1\WURTHWE\12-Feb-2020\0\\ # pbe1pbe/def2tzvp Opt=readfc geom=check guess=read Pop=NBO Freq emp=gd3bj nosym scrf=(solvent=dichloromethane)\\ Compound 1b: Thiofluorenone\\0,1\S,0.1246976562,0.0000000006,0.0178002009\C,-0.0131962887,-0.0000000004,1.6402010187\C,1.0777841126,-0.0000000001,2.6256638253\C,-1.2550507172,0.0000000002,2.427050491\

C,0.5300290741,0.0000000002,3.9208007862\C,1.3559727108,0.0000000021,
5.0266308363\C,2.7362253914,0.0000000015,4.8234718862\C,3.2767734922,
0.0000000001,3.5427505382\C,2.4457487001,-0.0000000008,2.427111056\
H,0.9509072867,-0.0000000011,6.0318979533\H,3.3992170317,0.0000000007,
5.6809962783\H,4.352480398,-0.0000000019,3.4145953824\H,2.8515486487,-
0.0000000004,1.4217696546\C,-0.9341845743,-0.0000000003,3.7961524253\C,-
1.9352584101,0.0000000009,4.746396586\C,-3.2612510691,-0.0000000004,
4.3126216722\C,-3.5774341649,-0.0000000004,2.9589332058\C,-2.5696852599,
0.0000000014,1.9999452337\H,-1.7062958095,-0.000000001,5.8057871028\H,-
4.0598270922,-0.000000001,5.0455663518\H,-4.6159469341,0.0000000005,
2.6506113429\H,-2.7994112263,-0.0000000004,0.9404108439\\Version=ES64L-
G16RevB.01\HF=-897.8817061\RMSD=7.138e-09\RMSF=3.640e-05\Dipole=-
0.1532857,0.,1.8070194\Quadrupole=3.0668293,-14.1035865,11.0367572,0.,-
0.244343,0.\PG=CS [SG(C13H8S1)]\\@

9c (from 1b and 7a)

HF=-2135.3001907 a.u (0)

1\1\GINC-R07N14\FOpt\RPBE1PBE\def2TZVP\C28H22N2O2S2\
WURTHWE\12-Feb-2020\0\\# pbe1pbe/def2tzvp Opt=readfc geom=check
guess=read Pop=NBO Freq emp=gd3bj nosym scrf=(solvent=dichloromethane)
\\Compound 9c\\0,1\C,-0.2377940873,-0.1215409627,-0.0220186334\C,-
0.3243952515,0.0156433215,1.3641924468\C,0.798517508,-0.1870400308,
2.1527629338\C,1.9941562239,-0.5269348576,1.5332595738\C,2.067602739,-
0.6702795481,0.15086197\C,0.9427185375,-0.4748936418,-0.641699038\C,-
1.6039048318,0.0742124001,-0.6412973976\N,-1.6693118969,1.009198295,-
1.7708803494\N,-2.7278173541,1.0276635896,-2.6160641151\C,-3.5875692291,
0.0916463027,-2.742468621\C,-3.6014019073,-1.1976146707,-1.9827033308\S,-
1.9965382261,-1.5826382324,-1.2952878039\C,-4.6708552887,0.3379152845,-
3.7169881191\C,-4.5709334846,1.3811365366,-4.6416153752\C,-5.5952875451,
1.6284914986,-5.5363409728\C,-6.7419949824,0.8418840234,-5.5262348808\C,-
6.8501012222,-0.1969163152,-4.6155224321\C,-5.8209664307,-0.4508312057,-
3.7200927831\S,-1.0855104718,2.5993812231,-1.6173005004\C,-2.3551858053,
3.4848899842,-0.8008146602\C,-3.5122400407,3.8284213376,-1.4883248215\C,-
4.5332756462,4.4641751123,-0.8044004127\C,-4.4135943968,4.7693157532,
0.5505935451\C,-3.2311710911,4.4343059425,1.2080656744\C,-2.2048460994,
3.7878196242,0.5458751683\C,-5.5378496136,5.4180255438,1.2933075001\C,-
2.4649221328,0.4421974522,0.5495572688\C,-3.7935973745,0.8248413672,
0.5989387603\C,-4.354896507,1.1275373882,1.8345610863\C,-3.5980344618,
1.0488367462,2.9971045924\C,-2.2598513749,0.679481731,2.9481623935\C,-
1.693970037,0.382720074,1.7179713256\O,0.0524687052,2.5234934614,-
0.7547918712\O,-0.9486008122,3.091653578,-2.9519980025\H,-3.6732233304,
1.9863529516,-4.6516156963\H,-5.4985267128,2.4370052183,-6.2518119756\H,-

7.5434633526,1.0369257806,-6.2291411831\H,-7.7390595437,-0.816571701,-4.5985038407\H,-5.9310631882,-1.2647397894,-3.0139032154\H,-4.3656906311,-1.1846244957,-1.1990086723\H,-3.8438049844,-2.0209734678,-2.6571977634\H,-5.4408637868,4.7322794249,-1.333840434\H,-3.6092269995,3.5950507023,-2.5403148865\H,-1.2956840975,3.510313788,1.0626516153\H,-3.117696906,4.6736102852,2.2595057529\H,-5.1661332174,6.1325614192,2.0296398452\H,-6.2182507738,5.9351544637,0.6160744752\H,-6.1179542605,4.6648992058,1.8357824254\H,-4.0532917417,1.2911582789,3.9503129541\H,-5.390736454,1.441141318,1.8863744046\H,-4.388496175,0.9330808386,-0.2983931911\H,-1.6689434551,0.6359977662,3.8558933176\H,0.7487983297,-0.0840152828,3.2306496163\H,2.8822520279,-0.6849747931,2.1340877996\H,3.0108491429,-0.9353418518,-0.311797676\H,0.9953085393,-0.5797463562,-1.7190358778\\Version =ES64L-G16RevB.01\HF=-2135.3001907\RMSD=5.733e-09\RMSF=1.055e-06\Dipole=-2.9401751,-0.2722377,1.6474728\ Quadrupole=24.8314556,-15.7934435,-9.0380121,-14.5513673,6.1943607,13.2720414\PG=C01 [X(C28H22N2O2S2)]\\@

1b + 7a-vdW-complex

HF=-2135.2450211 a.u (0)

1\1\GINC-R08N07\FOpt\RPBE1PBE\def2TZVP\C28H22N2O2S2\WURTHWE\12-Feb-2020\0\\# pbe1pbe/def2tzvp Opt=readfc geom=check guess=read Pop=NBO Freq emp=gd3bj nosym scrf=(solvent=dichloromethane)\\1b + 7a-vdW-complex \\0,1\C,-1.6881929114,-1.4618646275,0.9727525651\C,-0.5982740521,-1.3144950797,1.9461219319\C,0.2296811578,-2.4470365056,1.8488893908\C,-0.2866247531,-3.3312342376,0.7977445769\C,-1.4942524783,-2.6699397838,0.2811181575\C,-0.2940611094,-0.3028214316,2.8339293413\C,0.8426132147,-0.4429433601,3.6308206313\C,1.6532367207,-1.5684496808,3.5432282747\C,1.3519813527,-2.5848841734,2.6439976871\S,0.3601692262,-4.737875772,0.2878972198\C,0.8970505524,-1.0455515666,-1.6329071448\C,1.5610162657,-0.3832392451,-0.6813266724\N,1.1489466457,0.9360725941,-0.3421753289\N,-0.0621172536,1.1131053235,-0.3144408895\C,-2.7578179241,-0.6461635937,0.6660971595\C,-3.6299296109,-1.0507395772,-0.3435455369\C,-3.4356213479,-2.2438935728,-1.0311392709\C,-2.3592023473,-3.0682120683,-0.7210447717\S,-0.4897301501,2.7689127939,0.0531955519\O,0.6695358976,3.600624105,0.1945215954\C,2.7816819711,-0.8233553651,0.0111474248\C,3.4780702332,0.0500869875,0.8466967721\C,4.6111241519,-0.3743748397,1.5236055701\C,5.064852664,-1.6761464599,1.3812822602\C,4.3736960296,-2.5547466517,0.555835264\C,3.2433499991,-2.13426739,-0.1202377262\C,-1.3383156905,3.1552059206,-1.4204650928\C,-0.6699842798,3.8219714472,-2.4401551147\C,-1.3438829595,4.0908090313,-3.6166127761\C,-2.6700485502,3.6951408066,-3.7942896158\C,-3.3152147354,3.0272906994,-2.7536380755\C,-2.6597550633,2.7519849238,-1.5680756187\C,-3.3771204814,3.9637982737,-

5.0835821252\O,-1.4226763031,2.6335701091,1.1369911193\H,3.1277103611,
1.0669298712,0.9657081551\H,5.1379229577,0.3182859504,2.1696939081\H,
5.9481267968,-2.0089669372,1.9138376921\H,4.7115197984,-3.5787180145,
0.4463739685\H,2.7030652289,-2.8438309618,-0.7341180143\H,0.0160865828,-
0.6096370861,-2.0848829868\H,1.2351457767,-2.0035993187,-2.001621325\H,-
0.8341576144,4.6213013458,-4.412999727\H,0.3581360476,4.1317413291,-
2.3021302816\H,-3.1644933452,2.2415269806,-0.7581161425\H,-4.3489821157,
2.7235779759,-2.8739801605\H,-4.4577522583,4.0048337151,-4.9437944674\H,-
3.0439840581,4.9016115537,-5.5302859492\H,-3.1670071053,3.1666041306,-
5.8032638726\H,-4.4747612401,-0.4216878968,-0.6004818599\H,-4.1285944301,-
2.5316120216,-1.8126179685\H,-2.1925336133,-4.0008001074,-1.2481955748\H,-
2.9086215679,0.294441538,1.1820875948\H,-0.9069050846,0.5881338247,
2.9006045115\H,1.1011670702,0.3455097931,4.328284552\H,2.5334429727,-
1.6460011246,4.1694944098\H,1.9834367589,-3.4602605733,2.5505758044
\\Version=ES64L-G16RevB.01\HF=-2135.2450211\RMSD=9.794e-
09\RMSF=1.503e-06\Dipole=-0.9998872,0.7428053,-2.0989043\
Quadrupole=9.9314628,-21.2366687,11.305206,-10.4764446,19.3715698,-
11.8520089\PG=C01[X(C28H22N2O2S2)]\\@

TS for 9c

HF = -2135.237493 a.u. (1, -186,7306cm^{-1})

1\1\GINC-R09N40\FTS\RPBE1PBE\def2TZVP\C28H22N2O2S2\
WURTHWE\13-Feb-2020\0\\# pbe1pbe/def2tzvp Opt=(ts,noeigentest,readfc)
geom=check guess=read Pop=NBO Freq emp=gd3bj nosym scrf=(solvent=
dichloromethane)\\ TS for 9c \\0,1\C,0.0807414012,-0.3208192441,
0.1532155652\C,0.7593555003,-0.1716399365,1.4442743735\C,2.0401427836,-
0.742834591,1.332100182\C,2.1970578459,-1.2903544179,-0.0159539435\
C,0.9533560703,-0.9936079201,-0.7246813148\C,0.3584974686,0.4134561772,
2.6279826125\C,1.2540996069,0.4329266208,3.6957038473\C,2.5237725845,-
0.1231712115,3.5805861129\C,2.9280642567,-0.7207304885,2.3926067234\
S,3.4949929036,-2.1133490966,-0.5807968673\C,4.258597415,-0.8958108083,-
2.4934785443\C,5.1231947104,-0.0425405775,-1.8565473047\N,4.6587279632,
1.1634833274,-1.3529846523\N,3.4220323522,1.2718712866,-1.2536520832\C,-
1.1713833415,0.0722521515,-0.2773605081\C,-1.5407124977,-0.2040432709,-
1.5938291311\C,-0.6782586158,-0.867055145,-2.4592279066\C,0.5792548678,-
1.2749087358,-2.0265595088\S,2.9418169572,2.8263332822,-0.6917590846\
O,4.0516129294,3.7295981111,-0.5739617574\C,6.554012799,-0.2950496487,-
1.6580981561\C,7.405915756,0.7323445149,-1.2419295684\C,8.7592225789,
0.4980705972,-1.0637602583\C,9.2895088481,-0.7650363159,-1.2859780387\
C,8.4499171269,-1.7958316454,-1.6875835056\C,7.0977900705,-1.5647329004,-
1.8714283238\C,1.9424123719,3.2891040184,-2.0500682011\C,2.4285676642,
4.183882283,-2.9929974227\C,1.6350250434,4.5111013678,-4.0786857145\

C,0.3689716958,3.9503289144,-4.2404695937\C,-0.0891499092,3.0453927983,-3.2820279395\C,0.6854529953,2.7122196973,-2.1875681447\C,-0.4916482296,4.3271135011,-5.4038638177\O,2.1097067078,2.5646300163,0.4486553412\H,6.9950080141,1.7170941128,-1.0628449571\H,9.4039289755,1.3095593029,-0.7462036594\H,10.347938104,-0.9476362021,-1.1420198045\H,8.8497640356,-2.7894657894,-1.853641068\H,6.4607488718,-2.3893760144,-2.1678718964\H,3.2716771793,-0.5350535976,-2.7501240707\H,4.6236486314,-1.7580390461,-3.0328252608\H,2.0058248036,5.2132500499,-4.8170971278\H,3.4134796092,4.6161885878,-2.8699568277\H,0.3253663319,2.0095717572,-1.4468923059\H,-1.0680068917,2.5930346158,-3.3977017976\H,-1.1556485156,5.1539326446,-5.1334431509\H,0.1087187257,4.6525927293,-6.2541887885\H,-1.1213416736,3.4930472783,-5.7172432399\H,-2.5181430691,0.1030986678,-1.9475835362\H,-0.9907016496,-1.0708681437,-3.4762314332\H,1.248153773,-1.8121283647,-2.6892701133\H,-1.8560614591,0.5890504871,0.3850451941\H,-0.623617893,0.8604310352,2.7298093519\H,0.9577554833,0.8940155214,4.6307436578\H,3.2025111447,-0.0882016977,4.4242410612\H,3.9164207877,-1.1543036459,2.2906503066\\Version=ES64L-G16RevB.01\HF=-2135.237493\RMSD=7.391e-09\RMSF=8.760e-07\Dipole=-1.7361092,-0.7548112,-2.1586515\ Quadrupole=-4.3087511,-12.3779693,16.6867204,-18.198321,-1.9559807,-8.0178744\PG=C01 [X(C28H22N2O2S2)]\\@

10c (from 1b and 7a)

HF=-2135.2941496 a.u. (0)

1\1\GINC-R08N08\FOpt\RPBE1PBE\def2TZVP\C28H22N2O2S2\WURTHWE\12-Feb-2020\0\\# pbe1pbe/def2tzvp Opt=readfc geom=check guess=read Pop=NBO Freq emp=gd3bj nosym scrf=(solvent=dichloromethane) \\Product 10c (NNS)\\0,1\C,0.2849395569,-0.4221579311,-0.1184568522\C,0.1958140135,-0.4958616191,1.2762535958\C,1.0933006334,0.1699627578,2.0886058104\C,2.1008260706,0.9189229363,1.4883581023\C,2.1948215021,0.9943246757,0.1028591742\C,1.290061439,0.3259973668,-0.712754956\C,-0.9876251096,-1.342560209,1.6771185213\C,-0.659717219,-2.454335196,2.6625061772\C,-1.8462903317,-3.0488209047,3.3691841685\N,-3.0195469565,-2.5398764127,3.4332165233\N,-3.3226973685,-1.3726316181,2.8290386422\S,-2.1169235758,-0.1752328331,2.5068533037\C,-1.6727749283,-4.3516087451,4.0436034184\C,-2.7856754556,-5.0849689102,4.4644629715\C,-2.6278083958,-6.2986647388,5.1064738461\C,-1.355169578,-6.8052123189,5.3467922103\C,-0.2446808026,-6.0866521021,4.934088355\C,-0.4010519083,-4.8709844497,4.2834359566\S,-4.8686718232,-0.8331734966,3.2198423765\C,-4.7915719783,-0.5103350286,4.9355645403\C,-5.0375520878,-1.5377081437,5.8377974146\C,-4.9084103369,-1.2864804535,7.1913099033\C,-4.5374983815,-0.0260123375,7.6592338944\C,-4.2938627036,0.9850136913,6.7311690645\C,-4.4150447232,0.7530494069,5.3725202616\C,-4.4304420947,0.240153605,9.1269722876\C,-

1.5348282266,-1.7954843373,0.3451460762\C,-2.5914580774,-2.6452473356,
0.0719282912\C,-2.9055517129,-2.9106270123,-1.2575706417\C,-2.1726686683,-
2.3388036436,-2.2912148918\C,-1.1025348552,-1.4962989494,-2.0193228366\C,-
0.784896937,-1.2306873439,-0.6962813243\O,-5.0156214058,0.3959168444,
2.5022773174\O,-5.7536426332,-1.930937109,2.9900844651\H,-3.7748402789,-
4.6889656742,4.2730042734\H,-3.5021674386,-6.8576678675,5.4194619422\H,-
1.2330241226,-7.7570574827,5.8503848718\H,0.7519621815,-6.4710793907
,5.1168920627\H,0.4822250141,-4.325912468,3.974868194\H,0.0439856417,-
2.0732363464,3.4074320181\H,-0.1422917589,-3.2534124114,2.1234926471\H,-
5.0981742108,-2.0844223921,7.9006315319\H,-5.3263828709,-2.5161202097,
5.4774098775\H,-4.2293210844,1.5433103144,4.6560696511\H,-4.004569477,
1.9705666279,7.0785058669\H,-3.7536192755,1.0702405445,9.3322861527\H,-
5.4097567868,0.5032018855,9.5383763963\H,-4.0777385444,-0.6413115286,
9.6645665231\H,-2.4354738066,-2.5584926673,-3.319472505\H,-3.7319883116,-
3.5725968209,-1.4879235141\H,-3.1703334516,-3.1004324764,0.8650929313\H,-
0.525545142,-1.0620831253,-2.8279356532\H,1.0183733732,0.1171384736,
3.1692955157\H,2.8175529329,1.4474713607,2.1054381364\H,2.9866070883,
1.5823679429,-0.346559694\H,1.3731804149,0.390112156,-1.791536346\\
Version=ES64L-G16RevB.01\HF=-2135.2941496\RMSD=4.562e-09\RMSF=
8.341e-07\Dipole=2.5312294,-0.407672,1.7674484\Quadrupole=-34.4240773,-
1.476824,35.9009013,0.4923791,-5.7444886,-5.1816626\PG=C01
[X(C28H22N2O2S2)]\\@

TS for 10c

HF=-2135.2276585 a.u (1, -278,3394cm^{-1})

1\1\GINC-R10N14\FTS\RPBE1PBE\def2TZVP\C28H22N2O2S2\
WURTHWE\06-Mar-2020\0\\# pbe1pbe/def2tzvp Opt=(ts,noeigentest,
readfc) geom=check guess=read freq pop=nbo emp=gd3bj nosym
scrf=(solvent=dichloromethane)\\TS for 10c\\0,1\C,-0.0600830678,-
0.0962309809,0.0617919924\C,0.0298259423,-0.1136481145,1.5221892221\
C,1.3863517473,-0.0266817468,1.8873210041\C,2.1967749667,0.0723687254,
0.6508213266\C,1.2461557743,-0.0258531803,-0.4580297608\C,1.772939051,-
0.0502781338,3.2141596148\C,0.7866793538,-0.1455658527,4.1889545098\C,-
0.5576874469,-0.218073944,3.835370247\C,-0.9467107599,-0.2061127949,
2.5000760553\C,-1.1425211371,-0.1508506942,-0.7969969194\C,-0.9045370882,-
0.143498297,-2.1699883134\C,0.3891534107,-0.0966038643,-2.6764682199\
C,1.4845864005,-0.0407414232,-1.8207371869\S,3.839985109,-0.151413937,
0.5202209505\N,4.3797305697,1.7646447362,-0.4759726472\S,5.5705000917,
1.2087377448,-1.622069065\O,4.9338578452,0.5839763752,-2.7456442843\
N,3.4455963591,2.375247924,-1.0864682837\C,2.3773923408,2.767541949,-
0.3468062794\C,1.2881368502,3.413118327,-1.0961106854\C,1.2479612352,
3.3446117885,-2.4897600052\C,0.2203699729,3.9474291213,-3.1947245618\C,-

0.7892637017,4.6213728191,-2.5215948915\C,-0.7562308186,4.6996399817,-1.1359395355\C,0.2762481735,4.1060422868,-0.4297538751\C,2.2820596652,2.4179237984,0.9774552719\C,6.2685620962,2.7304321882,-2.1232445574\C,5.7838460279,3.3625242678,-3.2606099679\C,6.3297971564,4.5761831243,-3.6349174497\C,7.3461023883,5.1708895139,-2.8868352002\C,7.8143856362,4.5113048464,-1.751150257\C,7.2812789004,3.2962923479,-1.3603424238\C,7.9060377817,6.4992155913,-3.2838430454\O,6.5135148002,0.4834390386,-0.8224879027\H,2.0235000722,2.7997304362,-3.0109204977\H,0.200855012,3.8800607647,-4.2763373767\H,-1.597234945,5.0866903269,-3.0741346383\H,-1.5338651735,5.2331950691,-0.6020731631\H,0.3011971038,4.2003754688,0.649136724\H,3.1731832583,2.1951467648,1.5447204593\H,1.3701772339,2.593030428,1.5293038753\H,5.9640433923,5.0722523049,-4.5269956494\H,4.9975072959,2.8997165381,-3.842303067\H,7.6508687448,2.7825869709,-0.4819669208\H,8.6127920592,4.9551001401,-1.1670881952\H,7.8755875286,6.6354227341,-4.3656748039\H,7.3190939634,7.3068018877,-2.8355529323\H,8.9358440185,6.6143919276,-2.9438406852\H,-1.7434185661,-0.1848620763,-2.8550874835\H,0.5448164995,-0.1013279735,-3.7486892405\H,2.4979873842,-0.0116963168,-2.2051399654\H,-2.1569759266,-0.2047404679,-0.4186403896\H,2.8214321458,0.0069094574,3.4841524054\H,1.0671843342,-0.1659397021,5.2354277474\H,-1.3106348695,-0.2919336853,4.6113346235\H,-1.9957125978,-0.27288308,2.2347796034\\Version=ES64L-G16RevB.01\HF=-2135.2276585\RMSD=5.342e-09\RMSF=1.057e-06\Dipole=-1.6640437,2.9624584,0.2110799\Quadrupole=-12.058361,8.6756078,3.3827532,29.0283882,-1.0187103,-12.0859347\PG=C01[X(C28H22N2O2S2)]\\@

1m

HF=-975.2167163 a.u. (0)

1\1\GINC-R10N32\FOpt\RPBE1PBE\def2TZVP\C15H10S1\WURTHWE\09-Feb-2021\0\ \# pbe1pbe/def2tzvp Opt=readfc geom=check guess=read Pop=NBO Freq emp= gd3bj nosym scrf=(solvent=dichloromethane)\\Educt 1m\\0,1\C,0.0456181386,-0.2770719488,0.019785706\C,0.0118539522,-0.0788157192,1.4158841719\C,1.2144407141,0.1619276201,2.0942119651\C,2.4224305389,0.2050909904,1.4328164972\C,2.4543307901,0.0565002988,0.0489500679\C,1.2809493787,-0.1643294134,-0.637401866\C,-1.2232492783,-0.0454347941,2.2102858899\S,-1.431437415,1.1231933263,3.3442372248\C,-1.1047761568,-0.540895469,-0.8124051315\C,-2.2852710658,-1.0819941299,-0.4719691179\C,-2.7297243683,-1.5490491581,0.820274292\C,-2.229156951,-1.1056292693,2.0622037072\C,-2.7544176602,-1.6567153283,3.2389121635\C,-3.7397021172,-2.6194868139,3.2103536661\C,-4.2689751933,-3.0255740794,1.9884299602\C,-3.7776819428,-2.4830594502,0.8218166452\H,-4.1918624884,-2.7891975903,-0.1323527469\H,-5.0621981509,-3.762827338,1.9521979767\H,-4.1079016172,-3.0432088329,4.1369076668\H,-2.3597176398,-1.3084063502,

4.1848668824\H,1.2986964084,-0.2727352985,-1.716137769\H,3.3941338164
,0.1140262683,-0.4872351492\H,3.3372545126,0.3697239463,1.9892331331\
H,1.1740231052,0.3104014554,3.1657788402\H,-2.9789165198,-1.2797297001,-
1.283619383\H,-0.947676498,-0.3482736448,-1.8693591459\\Version=ES64L-
G16RevB.01\HF=-975.2167163\RMSD=7.360e-09\RMSF=1.860e-06\Dipole=
0.2348835,-1.2229344,-1.1259307\Quadrupole=6.6605945,-4.2182755,-
2.442319,9.762549,2.6896425,-5.7201003\PG=C01[X(C15H10S1)]\\@

9m (from 1m and 7a)

HF=-2212.6224788 a.u. (0)

1\1\GINC-R10N11\FOpt\RPBE1PBE\def2TZVP\C30H24N2O2S2\
WURTHWE\10-Feb-2021\0\\# pbe1pbe/def2tzvp Opt=readfc geom=check
guess=read Pop=NBO Freq emp=gd3bj nosym scrf=(solvent=dichloromethane)
\\Product 9m (NNC) \\0,1\C,0.0392424484,0.267478909,0.0656142486\
C,0.0048574585,0.1491152349,1.4692274175\C,1.2286456994,0.0804175504,
2.1356380479\C,2.4483187586,0.1524304491,1.4825067206\C,2.4803421455,0.28
71544827,0.1070262807\C,1.2829106827,0.3336651089,-0.5773497514\C,-
1.2526222285,-0.0733588183,2.3096571286\N,-0.9323938159,0.181133242,
3.7317495184\N,-0.4993853048,-0.7071477406,4.6456994657\C,-0.214866704,-
1.9284591369,4.3936513545\C,-0.3486374459,-2.5607376548,3.0453579375\S,-
1.7426863918,-1.8438618838,2.1789088866\C,-2.592199888,0.6080505345,
1.9809587399\C,-3.0672070251,0.8596712404,0.6835906293\C,-4.361015373,
1.3879342509,0.5323324004\C,-5.1881504876,1.6461685632,1.6020969157\C,-
4.738453697,1.3361188733,2.8760733479\C,-3.4699771402,0.8164104012,
3.046460294\C,-2.3862213547,0.5808218095,-0.5637961182\C,-1.1008502876,
0.3228610175,-0.8218706604\S,-0.7655680507,1.7455349488,4.3553544972\O,-
1.5695919511,1.8181592377,5.5385653018\C,0.2825052216,-2.7292279391,
5.5263830792\C,0.0323062236,-2.3326289047,6.8422101976\C,0.5284858476,-
3.0665029018,7.9040610753\C,1.2826722741,-4.2114683469,7.6735389287\
C,1.5310960706,-4.6170750711,6.3713143394\C,1.0306382468,-3.885081659,
5.3045896816\C,0.910755261,1.8964486063,4.8461366118\C,1.3439778377,
1.3286066905,6.0387253408\C,2.6703357425,1.4604421115,6.4019177955\
C,3.572307836,2.1626021398,5.6005765882\C,3.108074025,2.7325604777,
4.4179415104\C,1.7849515069,2.6018805939,4.0321654284\C,4.9990726124,2.31
80904267,6.0208546937\O,-1.0083547353,2.6275866029,3.2540365267\H,-
0.5681007606,-1.4485709427,7.0166874157\H,0.3190718495,-2.7501443469,
8.9194686859\H,1.6682244511,-4.7872049921,8.5068123381\H,2.1182447392,-
5.5079105726,6.1809941045\H,1.2452622787,-4.21187918,4.2939678983\
H,0.5683953966,-2.4536336183,2.4574635761\H,-0.5683160745,-3.6246591753,
3.136247697\H,3.0149768146,1.0109219051,7.3267064138\H,0.6481894705,
0.7863186173,6.6636729379\H,1.4290118239,3.0367901852,3.1076400102\H,3.79
41668165,3.283201007,3.7842012892\H,5.6327393119,2.6035494372,

5.1807655027\H,5.0890297456,3.0955309005,6.7854586719\H,5.3861099882,
1.393584666,6.4532651691\H,3.4206862527,0.3454201021,-0.4278144949\
H,3.363169998,0.0971906335,2.0601823938\H,1.2548758273,-0.0444593415,
3.2065103709\H,1.2877356091,0.4290684464,-1.6575278766\H,-4.7095183998,
1.5867479282,-0.4750196696\H,-6.177284953,2.0590128051,1.4440980355\H,-
5.373593703,1.4846456553,3.7410807953\H,-3.1608436712,0.5571181708,
4.0481531966\H,-3.0396221702,0.6273418889,-1.4297465944\H,-0.8460223442,
0.1885717001,-1.868676767\\Version=ES64L-G16RevB.01\HF=-2212.6224788\
RMSD=3.298e-09\RMSF=6.082e-07\Dipole=2.7533317,-1.3979449,-0.247879\
Quadrupole=7.588917,-9.9339671,2.3450501,2.6115682,26.8141571,-
14.7629818\PG=C01[X(C30H24N2O2S2)]\\@

vdW (from 1m and 7a)
HF=-2212.5793122 a.u. (0)
1\1\GINC-R08N27\FOpt\RPBE1PBE\def2TZVP\C30H24N2O2S2\
WURTHWE\21-Mar-2021\0\\# pbe1pbe/def2tzvp Opt Pop=NBO Freq
emp=gd3bj nosym scrf=(solvent=dichloromethane)\\vdw-complex from 1m
and 7a\\0,1\C,-0.0235162154,-0.0359298647,-0.0008371596\C,-0.0006017884,-
0.0471329109,1.3883049216\C,1.197579799,-0.0148630903,2.0912766579\
C,2.3845397283,0.0463989453,1.3856549583\C,2.392565035,0.0664336601,-
0.0096266556\C,1.1748644257,0.0198137142,-0.6874009906\S,-1.5078487343,-
0.1678814527,2.2568774554\O,-2.5606424248,0.4556213425,1.5092938148\
C,3.6813191309,0.1592443809,-0.7613534661\N,-1.7982845063,-1.8825838747,
2.0269302906\N,-1.7758005263,-2.4995359367,3.0836643676\C,-1.9362713787,-
3.9069120864,2.9694408308\C,-2.7328221664,-4.5031762686,4.0518714647\C,-
2.9152284468,-3.8302128813,5.2603288846\C,-3.6909614558,-4.3900298854,
6.2634748489\C,-4.3010835915,-5.6210343108,6.0730205619\C,-4.1304854639,-
6.2928381947,4.8690176501\C,-3.3554671313,-5.7379233634,3.8657040579\C,-
1.2998418537,-4.5670174905,1.9991913921\O,-1.3105340912,0.1513994485,
3.6410103157\C,-5.3535452522,-0.4304196085,-0.7129311411\C,-5.3833563586,-
1.2902792786,0.4446367322\C,-4.8164439133,-2.5802470711,0.5209270051\C,-
4.962390221,-3.3066437037,1.7105500976\C,-5.6072888933,-2.7864739664,
2.8097667628\C,-6.1876308393,-1.5235816426,2.7289121145\C,-6.0915085442,-
0.8079866162,1.5565164553\C,-4.1102259767,-3.2486772875,-0.5776542608\S,-
4.3445502706,-4.8565411001,-0.8413702521\C,-3.1222947059,-2.5291033438,-
1.3941596663\C,-3.3344788854,-1.2546357639,-1.9608162303\C,-2.3693397095,-
0.752928401,-2.8509823966\C,-1.2000243577,-1.4284137456,-3.1172261786\C,-
0.9637610408,-2.6560589312,-2.504035713\C,-1.9241527206,-3.1994200707,-
1.6802847798\C,-4.4906591713,-0.4207968956,-1.7406967922\H,-2.445996572,-
2.8666218036,5.409757516\H,-3.821774989,-3.8573171121,7.198182799\H,-
4.9139726359,-6.0532702418,6.8552416458\H,-4.6142715988,-7.2487121547,
4.7058993394\H,-3.254758106,-6.255800282,2.9188190204\H,-0.7277789429,-

4.0265110057,1.2565928805\H,-1.2970283504,-5.6478512567,1.9626292221\H,3.3243992007,0.0773802899,1.9254010761\H,1.1904721694,-0.0323219838,3.1736564474\H,-0.9660782491,-0.0721926024,-0.5322866688\H,1.1665066509,0.0249445233,-1.7714281853\H,3.5841208471,-0.2475367707,-1.7684482106\H,3.9901759706,1.2048416913,-0.8557717621\H,4.4817850741,-0.3707126844,-0.243101086\H,-0.4690792699,-1.0022839921,-3.7943008225\H,-0.0394642946,-3.1918448552,-2.6833372903\H,-1.7687541149,-4.1719935701,-1.232026314\H,-2.5582302587,0.2054414374,-3.3215390267\H,-6.5543108384,0.1696322707,1.4821136403\H,-6.7145042196,-1.1055770944,3.5784745286\H,-5.6620494435,-3.3644831488,3.7246627002\H,-4.5325376107,-4.2973088336,1.7534641696\H,-6.0950341994,0.3626402341,-0.6995107307\H,-4.6132449563,0.3764640015,-2.4673573882\\Version=ES64L-G16RevB.01\HF=-2212.5793122\RMSD=6.750e-09\RMSF=3.204e-06\Dipole=2.2740592,0.1364263,-0.6285507\Quadrupole=0.7196855,-4.465192,3.7455065,-9.2594486,-11.1557799,-17.3996976\PG=C01[X(C30H24N2O2S2)]\\@

TS for 9m

HF=-2212.5687574 a.u (1, -262.4891cm^{-1})

1\1\GINC-R09N27\FTS\RPBE1PBE\def2TZVP\C30H24N2O2S2\WURTHWE\13-Feb-2021 \0\\# pbe1pbe/def2tzvp opt=(ts,noeigentest,readfc) geom=check guess=read scrf=(solvent= dichloromethane) freq pop=nbo emp=gd3bj nosym\\ TS for 9m \\0,1\C,0.0922161821,1.1673964191,0.3306975444\C,0.4340938887,0.0244420502,1.097713702\C,1.8066990755,-0.2226259746,1.2934794969\C,2.7957041255,0.5856586812,0.7883384358\C,2.4539591892,1.6883243011,0.0099379559\C,1.1251801585,1.954493687,-0.2132963806\C,-0.5012594882,-0.9484357765,1.6717541928\N,0.4096254564,-0.0544817047,4.4183989466\N,-0.1107375071,-0.8719367004,5.2150066239\C,0.1078222477,-2.2006576198,4.9431902312\C,1.0475697415,-2.5702571508,3.9996598674\S,0.0687939273,-2.5266430032,1.898023826\C,-1.8795693729,-0.6803442553,2.0758269186\C,-2.7263091286,0.3455953543,1.5761173097\C,-4.0253734594,0.4637214131,2.0944725781\C,-4.4968923616,-0.3439061075,3.103782395\C,-3.6760030887,-1.3514534528,3.5979973396\C,-2.4188815498,-1.5231686551,3.0685134579\C,-2.4148290287,1.2756150036,0.5288977986\C,-1.2303503399,1.6149835248,-0.0021473683\S,0.0239910811,1.5613873209,4.813358386\O,-0.4832560829,1.6893300341,6.151688273\C,-0.7530628366,-3.1422525591,5.6640446464\C,-1.4716475667,-2.7329445418,6.7922003412\C,-2.2971132631,-3.6191852988,7.462047286\C,-2.4344127873,-4.9278893098,7.0172429306\C,-1.7355170214,-5.3412320855,5.8915897026\C,-0.9036480008,-4.4594642191,5.221511402\C,1.6185537722,2.2769439203,4.7204457142\C,2.3950116167,2.3799349247,5.8675081571\C,3.6586809197,2.9340572542,5.7757694008\C,4.1611711286,3.383371604,4.5542916772\C,3.3584762836,3.270799226,3.4203672527\C,2.0918024048,2.7195156497,3.4933432952\

C,5.54082474,3.9535676979,4.4615566531\O,-0.7649916666,2.0394122726,
3.7100780344\H,-1.3745343999,-1.7111785719,7.1351634524\H,-2.8404085542,-
3.2843378088,8.3383447871\H,-3.085112078,-5.6185341881,7.5406569508\H,-
1.8428116942,-6.3562943269,5.5268573444\H,-0.3865700475,-4.7968840943,
4.3313279104\H,1.790575147,-1.8322283949,3.731279581\H,1.3426157084,-
3.6069476952,3.9080166894\H,4.2665889021,3.0264525563,6.6689832214\
H,2.0041107047,2.0391943327,6.8180949128\H,1.4689354911,2.6368497659,
2.6119738145\H,3.7311776438,3.6231429295,2.4647944739\H,5.6297311727,
4.6440808243,3.6220434311\H,5.8157814076,4.4783681079,5.377668564\
H,6.2736116938,3.1548665661,4.3104255159\H,3.2192028332,2.3278595908,-
0.4134769153\H,3.8339973983,0.356345721,0.9949070664\H,2.0882725053,-
1.0940374481,1.8629902332\H,0.8431887372,2.8064079171,-0.8211241474\H,-
4.6652811267,1.2361981884,1.6838176226\H,-5.4977624883,-0.2022765832,
3.4937087127\H,-4.0190077809,-2.0121469769,4.385090106\H,-1.8118540545,-
2.3393536618,3.4257219857\H,-3.2750962474,1.8199714991,0.1521234388\H,-
1.2557742627,2.3951588267,-0.7562997759\\Version=ES64L-G16RevB.01\HF=-
2212.5687574\RMSD=5.700e-09\RMSF=1.845e-06\Dipole=1.7986227,
0.3263485,-1.3403736\Quadrupole=22.2068619,1.2028199,-23.4096818,
13.723574,13.004707,-13.3242687\PG=C01 [X(C30H24N2O2S2)]\\@

10m (from 1m and 7a)

HF=-2212.6262902 a.u (0)

1\1\GINC-R08N03\FOpt\RPBE1PBE\def2TZVP\C30H24N2O2S2\
WURTHWE\10-Feb-2021\0\\# pbe1pbe/def2tzvp opt=readfc geom=check
guess=read pop=nbo freq emp=gd3bj nosym scrf= (solvent=dichloromethane)
\\Product 10m (NNS)\\0,1\C,-0.0450558047,0.4566567639, 0.2314111631\C,-
0.1207669517,0.067987743,1.5677072743\C,1.0582159931,0.0924342329,
2.3371949431\C,2.2373505283,0.5739371321,1.7553645868\C,2.2899497967,0.96
37776597,0.433290356\C,1.1398638869,0.8892668178,-0.3378943512\C,-
1.4539516117,-0.3192045212,2.1806314394\C,-2.642696495,-0.0344803135,
1.2626574008\C,-2.8662478759,1.4211481229,0.9434735235\N,-2.4704210217,
2.4161364734,1.6433939332\N,-1.8488767344,2.2108886018,2.8286939778\S,-
1.8395195383,0.7298923946,3.6510053359\C,-1.4494389584,-1.7576918042,
2.6652044406\C,-0.599822354,-2.1652157432,3.7127687396\C,-0.7273429011,-
3.4668223139,4.2170270426\C,-1.6056044685,-4.3772045417,3.6678296126\C,-
2.3938750878,-3.9928511432,2.5922741506\C,-2.3203952735,-2.6967367808,2
.1131312277\C,0.447700203,-1.3480143335,4.2966163391\C,1.1624060477,-
0.3815619206,3.7027506181\C,-3.5596926617,1.7569288145,-0.3171232321\C,-
3.4358603768,3.0335471825,-0.8718326155\C,-4.0804189319,3.353369569,-
2.0520823985\C,-4.8693565335,2.4085466186,-2.6991350605\C,-5.0026509791,
1.1408789521,-2.1555192117\C,-4.3482610926,0.8145533832,-0.9761837251\S,-
0.7704549149,3.4474301028,3.2108097078\O,0.140892772,3.6718352,

2.1289021139\C,-1.8477307632,4.8221221466,3.3221439954\C,-1.8959126055,
5.7443600888,2.2899155139\C,-2.7595872138,6.8228141176,2.3911761217\C,-
3.5741792318,6.9892078842,3.5076645562\C,-3.5061735047,6.0421606473,
4.5318633863\C,-2.6511019854,4.960918283,4.4475720096\C,-4.492405351,
8.1641037224,3.6230758818\O,-0.2665056606,3.108521436,4.508281815\H,-
2.8209983155,3.7659133343,-0.3642688895\H,-3.9657817764,4.3450530028,-
2.4744290572\H,-5.3749776992,2.6613647152,-3.62378381\H,-5.6184378059,
0.3981812551,-2.649182349\H,-4.4723606131,-0.1801579101,-0.565780947\H,-
3.5417826511,-0.4397735827,1.7304386229\H,-2.5224648257,-0.5664521666,
0.3165623363\H,-2.8034182909,7.547257417,1.5856617937\H,-1.2636978011,
5.613252193,1.4215576985\H,-2.6015639175,4.2301463721,5.2455084401\H,-
4.135370863,6.1570287448,5.4076388699\H,-5.4776786188,7.8595587965,
3.9817559062\H,-4.0977985711,8.889666339,4.3404104928\H,-4.6119613639,
8.6711587742,2.6654499468\H,3.2183890717,1.3242201111,0.0066442052\
H,3.1311138167,0.6170097181,2.3681940146\H,-0.9271260664,0.4444002949,-
0.3937208576\H,1.1536915505,1.1907889833,-1.3785492659\H,-2.9695915173,-
2.4215442987,1.2939482746\H,-3.0842780755,-4.6934011438,2.1378007269\H,-
1.6729620179,-5.3810941472,4.0699476208\H,-0.0917047026,-3.7602401768,
5.0453356674\H,1.9882037832,0.0381181728,4.2693680488\H,0.7492948461,-
1.6405088314,5.29799 50339\\Version=ES64L-G16RevB.01\HF=-2212.6262902\
RMSD=3.739e-09\RMSF=1.187e-06\Dipole=-1.9556518,-0.2840783,-0.9928155\
Quadrupole=1.6054277,12.5352795,-14.1407072,-13.1597633,-2.8730922,-
6.3250355\PG=C01[X(C30H24N2O2S2)]\\@

TS for 10m

HF=-2212.5358951 a.u (1, -251.2265cm^{-1})

1\1\GINC-R10N02\FTS\RPBE1PBE\def2TZVP\C30H24N2O2S2\
WURTHWE\12-Feb-2021 \0\\# pbe1pbe/def2tzvp opt=(ts,noeigentest,readfc)
geom=check guess=read freq pop=nbo emp=gd3bj nosym scrf=(solvent=
dichloromethane)\\ TS for 10m\\0,1\C,-0.3625619924,-0.1073322324,-
0.0545180222\C,0.0276093285,-0.3774399464,1.2506331404\C,1.3603439653,-
0.769473139,1.49634231\C,2.2744232893,-0.7451897006,0.4382821044\
C,1.8822921237,-0.4218415246,-0.8462680502\C,0.5501308695,-0.1255888938,-
1.0961060841\C,-0.856568696,-0.2852717446,2.4502395305\C,-
2.9602967533,0.6931557089,1.5990469356\C,-2.6758449835,1.9101485632,
1.0348958545\N,-2.1840861062,2.929179832,1.793520625\N,-2.0671666778,
2.7021735221,3.0267410008\S,-0.6380622465,0.9269581834,3.5650957235\C,-
1.2902325147,-1.6176171254,2.9761303679\C,-0.2816043122,-2.3697943789,
3.6177563355\C,-0.6331148743,-3.5769400432,4.2309479859\C,-1.9227384606,-
4.0676483234,4.1777874068\C,-2.8991430356,-3.3413656376,3.5121265912\C,-
2.5826998295,-2.1281120824,2.9228099511\C,1.1138738446,-1.963077085,
3.6855240619\C,1.8215113857,-1.2635233758,2.7817937376\C,-2.7469110727,

2.1840955049,-0.4071846091\C,-1.8860916297,3.1135595196,-0.992846799\C,-1.9358799546,3.3514308946,-2.3558803903\C,-2.8417327913,2.6644360595,-3.1531541728\C,-3.7053878537,1.7423176682,-2.5779143375\C,-3.6616176921,1.5062810982,-1.2136560212\S,-1.2814663226,4.0456078287,3.8179216237\O,-0.2180960065,4.5547594403,3.0026364703\C,-2.5927979711,5.1997216081,3.8599545684\C,-2.7211520391,6.12659388,2.8330026352\C,-3.778676808,7.0158618822,2.8641208199\C,-4.7110992096,6.9917122313,3.9022577692\C,-4.5600866198,6.0462546892,4.9153586374\C,-3.5087192517,5.1471885857,4.9021099088\C,-5.8338914282,7.9783608222,3.9376018286\O,-1.0190856734,3.5635741251,5.1418155906\H,-1.1674151892,3.6308150646,-0.3701480185\H,-1.256540168,4.0692717949,-2.8004978124\H,-2.8770044426,2.8495533733,-4.2203802961\H,-4.4241836809,1.2128784128,-3.1921313784\H,-4.3584241942,0.8055405803,-0.7682536685\H,-3.1832720717,0.6367892174,2.6539486478\H,-3.2509179839,-0.1341849555,0.9656730072\H,-3.8854230794,7.7443021065,2.0680206753\H,-1.9974994564,6.1461333682,2.0285141905\H,-3.3905482476,4.4159526374,5.6916378419\H,-5.2778926674,6.014451311,5.7272490912\H,-6.2018098016,8.1953275042,2.9335392517\H,-6.6635757728,7.6155365055,4.5450035909\H,-5.4943842503,8.9239707557,4.3712476976\H,2.6076616441,-0.4153073237,-1.6511337094\H,3.3053957545,-1.0155406289,0.6397886983\H,-1.3889031002,0.1419215579,-0.2694955218\H,0.2169536698,0.1117670094,-2.0996238807\H,-3.3700895378,-1.5745887615,2.4353510546\H,-3.9175690601,-3.7081640026,3.4625481032\H,-2.16674915,-5.0099065148,4.6536505776\H,0.1393605261,-4.1382086863,4.7454445533\H,2.8857853132,-1.1534004582,2.9672105349\H,1.6624869466,-2.3644732819,4.5324777413\\Version=ES64L-G16RevB.01\HF=-2212.5358951\RMSD=8.226e-09\RMSF=5.900e-07\Dipole=-2.5653294,-0.4188974,-1.8646733\Quadrupole=16.483117,5.4248372,-21.9079542,-24.021884,-11.4863347,-12.3770406\PG=C01[X(C30H24N2O2S2)]\\@

1n

HF=-976.4316442 a.u. (0)

1\1\GINC-R10N32\FOpt\RPBE1PBE\def2TZVP\C15H12S1\WURTHWE\10-Feb-2021\0\ \# pbe1pbe/def2tzvp Opt=readfc geom=check guess=read Pop=NBO Freq emp= gd3bj nosym scrf=(solvent= dichloromethane)\\Educt 1n\\0,1\C,-0.0509744032,0.6190787537,0.3832179939\ C,0.1944886834,0.185313682,1.6973866971\C,1.3489554061,-0.5800467844,1.9514656737\C,2.1906646844,-0.8754182027,0.8784344421\C,1.9229318935,-0.4602986227,-0.4112193653\C,0.7862123112,0.2976939564,-0.6618445845\C,-0.837181708,0.5343327217,2.6947761949\S,-1.6534688926,1.9527113114,2.6299725589\C,-1.2168342453,-0.452271419,3.7269054197\C,-2.5456871547,-0.8654340752,3.817201646\C,-2.9336881592,-1.7773637714,4.7832250598\C,-2.0001916232,-2.2579957049,5.6909443489\C,-0.6830862116,-1.8307244924,5.6169925512\C,-0.2721895849,-0.9392842464,4.6336450308\

H,3.0929097332,-1.4445342682,1.0779360235\H,2.6002889406,-0.7205293722,-1.2163051803\H,0.5535272305,0.6316877009,-1.6658082138\H,-0.9452540876,1.2015967488,0.2025426131\H,0.044573353,-2.1903101223,6.3369618212\H,-2.2987177678,-2.9608183011,6.4600883708\H,-3.9654520784,-2.1046138335,4.8327331791\H,-3.2677307054,-0.4673623168,3.1143319119\C,1.8163124223,-1.07522984,3.2951764678\C,1.1480869566,-0.4901953329,4.5263421069\H,1.7038862576,-2.1660717998,3.3166743428\H,2.892377929,-0.887783256,3.353670255\H,1.7012824554,-0.8073522662,5.4121898469\H,1.2000709752,0.6035040983,4.4921397743\\Version=ES64L-G16RevB.01\HF=-976.4316442\RMSD=7.008e-09\RMSF=8.890e-06\Dipole=1.1466488,-1.4051155,0.0378185\Quadrupole=1.9084913,-6.874804,4.9663128,1.3825374,5.5626083,-11.3290622\PG=C01[X(C15H12S1)]\\@

9n (from 1n and 7a)

HF=-2213.8438687 a.u. (0)

1\1\GINC-R09N43\FOpt\RPBE1PBE\def2TZVP\C30H26N2O2S2\WURTHWE\10-Feb-2021\0\\# pbe1pbe/def2tzvp Opt=readfc geom=check guess=read Pop=NBO Freq emp=gd3bj nosym scrf= (solvent= dichloromethane)\\ Product 9n NNC\\0,1\C,-0.0704597294,-0.0708985487,-0.1370535199\C,0.205855136,-0.3167091785,1.2106255679\C,1.5418550078,-0.4148192127,1.6082863946\C,2.5931624322,-0.2968864662,0.7214306838\C,2.3156048282,-0.0964346462,-0.6219600914\C,1.0024565561,0.0059211514,-1.0325553085\C,-1.456741157,0.07923746,-0.7936734877\N,-1.3652342635,1.1675986592,-1.7908940676\N,-1.8827651823,1.1836422117,-3.0313006679\C,-2.5977183665,0.2484670952,-3.5318595579\C,-2.9372588674,-1.0230964052,-2.8215844376\S,-1.5811219545,-1.494751616,-1.7516962885\C,-3.103628484,0.4738792401,-4.8977858374\C,-2.4888932942,1.4026319873,-5.7407849818\C,-2.9834642222,1.6349463945,-7.0110959566\C,-4.1012696277,0.9443295176,-7.4656677251\C,-4.7153940467,0.0164277548,-6.6387872467\C,-4.2178817095,-0.2218623264,-5.3659419224\S,-0.784647435,2.7041268421,-1.4006446958\C,-2.1307210493,3.7899237321,-1.6917216352\C,-2.4034856135,4.2371713697,-2.9787854586\C,-3.4767567149,5.0829322226,-3.1828508903\C,-4.2797679786,5.4999693565,-2.1200446736\C,-3.9770849254,5.0456908078,-0.8388798323\C,-2.910206488,4.1918309715,-0.6166713725\C,-5.4235572006,6.4345571246,-2.3544476077\C,-2.7476016799,0.2206794073,0.0081036037\C,-3.7539872576,1.038816304,-0.5037947464\C,-5.0045446468,1.1277207602,0.088740568\C,-5.2821644474,0.378963421,1.2161952756\C,-4.2931889022,-0.4432044753,1.7333049494\C,-3.0350308987,-0.5407288765,1.1504013674\O,-0.5126664937,2.6734562393,0.0048840245\O,0.2510931328,3.0277108494,-2.3347875873\H,-1.610778448,1.9280834079,-5.3870595508\H,-2.4900946384,2.3530428224,-7.6560587017\H,-4.4860977628,1.1254904603,-8.4624476918\H,-5.5876644644,-0.526825841,-6.9832046894\H,-4.7183979826,-0.9429464791,-4.7305375727\H,-

3.8672682516,-0.9322446947,-2.2517503932\H,-3.0548855746,-1.8416847169,-
3.5320800852\H,-3.6988086104,5.4273731824,-4.1869293951\H,-1.7833458823,
3.917047895,-3.804587664\H,-2.682178309,3.8339086189,0.3786677925\H,-
4.5885260754,5.3613123359,-0.0009367137\H,-6.1298430203,6.4157981413,-
1.5239918132\H,-5.0617094151,7.4616443295,-2.4608838513\H,-5.9565122456,
6.1828928253,-3.2731533658\H,-6.2533366515,0.4328877536,1.6940731941\H,-
5.751649351,1.784114223,-0.3415715203\H,-3.5777911924,1.6270293236,-
1.3915650528\C,-2.0026026827,-1.3844504752,1.8194387306\H,-4.4931275531,-
1.0280556554,2.6248217749\C,-0.8275198353,-0.5270430464,2.2751521896\
H,1.7468129725,-0.6015126345,2.6571640178\H,3.6156015015,-0.3767353514,
1.0718912631\H,3.1143441595,-0.0197843182,-1.3499634192\H,0.7963251602,
0.1661449205,-2.0826259731\H,-0.335511538,-1.0017253098,3.12624502\H,-
1.2056547669,0.436140216,2.6353480159\H,-2.4562587735,-1.8779745999,
2.6804115766\H,-1.6436026242,-2.1728710093,1.1497572803\\Version=ES64L-
G16RevB.01\HF=-2213.8438687\RMSD=4.448e-09\RMSF=1.295e-06\Dipole=-
3.0114955,-0.1729583,-0.2421688\Quadrupole=11.4464035,-11.4719434,
0.0255399,-16.7228691,14.1098468,-6.9212651\PG=C01
[X(C30H26N2O2S2)]\\@

1n + 7a -vdW-Complex
HF=-2213.795258 a.u (0)
1\1\GINC-R07N15\FOpt\RPBE1PBE\def2TZVP\C30H26N2O2S2\
WURTHWE\02-Mar-2021\0\\# pbe1pbe/def2tzvp opt=(maxstep=10,readfc)
geom=check guess=read scrf=(solvent=dichloromethane) freq pop=nbo
emp=gd3bj nosym\\1n + 7a -vdW-Complex \\0,1\C,0.3102227815,
0.1114224377,0.3442135861\C,-0.0085495082,-0.2419774772,1.658082551\
C,1.0095410412,-0.3601279363,2.6057976043\C,2.326277661,-0.1095022794,
2.267094535\C,2.6466243917,0.2316825506,0.9602459013\C,1.6420325129,
0.3340180316,0.0111497272\C,-1.3955381167,-0.5658133675,2.0548445925\S,-
1.6537228096,-1.9981462654,2.8162410058\C,-0.7727341223,0.2307062453,-
0.6762838454\C,-1.6573835033,1.430247229,-0.3938640566\C,-2.5697989347,
1.3414913324,0.7976372662\C,-2.48534958,0.4080775841,1.8499701445\C,-
3.4809659292,0.4290011297,2.8424299609\C,-4.5032091796,1.3498181058,
2.8415564878\C,-4.5716070917,2.2822719256,1.8144619911\C,-3.623164468,
2.2557211347,0.8116324294\C,-0.9190918292,1.038772081,5.6663954984\C,-
1.8894537252,1.9166052094,5.4009531442\C,-3.2507471579,1.9027163415,
5.9551075607\C,-4.00717408,3.072661463,6.022695694\C,-5.3018363768,
3.042746928,6.517204104\C,-5.8615836808,1.8474563858,6.9432874292\C,-
5.1154074099,0.677400241,6.8741368332\C,-3.8222539036,0.7039160566,
6.3824965666\N,-1.5944670251,3.063911727,4.6168063218\N,-0.8815125121,
2.8652877089,3.6416705014\S,-0.4606730153,4.358178873,2.8211303253\O,-
1.0752590443,5.4928619683,3.4464864089\C,1.2530136082,4.340173068,

3.1437234583\C,1.7695460513,5.1324972195,4.1613195116\C,3.1254994525,5.08
0369556,4.4254933849\C,3.9692867821,4.2421525463,3.6961359775\
C,3.4192236105,3.4479810995,2.6902315141\C,2.0674080123,3.489352945,
2.4057580515\C,5.4380892503,4.2125900838,3.9726209434\O,-0.7000689243,
4.0751725144,1.4357039275\H,-3.5766387306,4.0058077893,5.6837210525\H,-
5.8772689121,3.9599318867,6.5648310627\H,-6.8769051352,1.8240631728,
7.3213566741\H,-5.5482658267,-0.263460991,7.193321637\H,-3.2615687763,-
0.2201094819,6.3033148127\H,0.0590852486,1.1581709202,5.2200251463\H,-
1.0682053634,0.2290801,6.3674479095\H,3.5390612986,5.6990553087,
5.2139610881\H,1.1135752029,5.7808190319,4.7281977313\H,1.645854307,
2.8673058834,1.626042682\H,4.0608877959,2.7831063331,2.1232841191\
H,5.8703248904,3.2436396725,3.7198716829\H,5.9518015438,4.9685453202,
3.3707004704\H,5.6496625075,4.4301116767,5.0203167666\H,3.6774383188,0.41
47304845,0.6791923838\H,3.1014531054,-0.1862889637,3.0202079779\
H,0.74896426,-0.6523442456,3.615214057\H,1.8898433349,0.5846366051,-
1.0148589023\H,-3.6904055951,2.9674613075,-0.0043674696\H,-5.3640201205,
3.0215046434,1.7925836161\H,-5.2286110334,1.3531749024,3.6461672819\H,-
3.4036181283,-0.2888547311,3.6474713226\H,-2.2844348433,1.6228332267,-
1.269014819\H,-1.0305087868,2.3201225569,-0.2689419887\H,-0.328346297,
0.3419037225,-1.6670297368\H,-1.3775802832,-0.6825636535,-0.6983191191\\
Version=ES64L-G16RevB.01\HF=-2213.795258\RMSD=6.813e-
09\RMSF=7.810e-07\Dipole=1.9706076,0.4338433,0.6749444\
Quadrupole=18.2804011,-27.6283066,9.3479055,11.9645084,7.0333533,
4.9192994\PG=C01[X(C30H26N2O2S2)]\\@

TS for 9n

HF=-2213.786918 a.u. (1, -221.4444cm^{-1})

1\1\GINC-
R10N19\FTS\RPBE1PBE\def2TZVP\C30H26N2O2S2\WURTHWE\12-Feb-
2021 \0\\# pbe1pbe/def2tzvp opt=(ts,noeigentest,readfc) geom=check
guess=read freq pop=nbo emp=gd3bj nosym scrf=(solvent=dichloromethane)
\\ TS for 9n \\0,1\C,-1.4721969372,-0.9363240016,-1.0618680254\C,-
0.3580709554,-0.6517403295,-0.2287512825\C,0.914227126,-0.708034966,-
0.7811542858\C,1.1422686255,-1.0636198054,-2.1016937683\C,0.0710769107,-
1.4289777771,-2.8981078875\C,-1.202214275,-1.3726948548,-2.3740397362\C,-
2.8787585058,-0.725886991,-0.7170689315\N,-2.5043890468,1.9480911508,-
2.1215149483\N,-1.994049307,1.617848853,-3.2145017577\C,-2.6837785556,
0.6748877835,-3.9471830916\C,-3.9723700009,0.337029018,-3.5989371046\S,-
4.081218307,-1.1950477605,-1.7906358795\C,-1.9665968452,0.1228161865,-
5.1022365328\C,-0.8107267544,0.7427785619,-5.5838373486\C,-0.1208000785,
0.2108342992,-6.6601547115\C,-0.5614137945,-0.9546495811,-7.2726297795\C,-
1.7055685715,-1.5816965887,-6.7978319266\C,-2.4025696712,-1.0481654802,-

5.7265244539\S,-1.5731387413,3.1291444097,-1.3024463993\C,-2.7677111158,
4.4031604142,-1.1821916766\C,-2.8050565582,5.4057706648,-2.1426812637\C,-
3.7679447,6.3936047111,-2.045048888\C,-4.6997308117,6.3917169817,-
1.0067495357\C,-4.6370558777,5.3744180693,-0.0554260866\C,-3.6792906449,
4.3793926059,-0.1353268612\C,-5.7550370416,7.4486227679,-0.9296525403\C,-
3.3742559303,-0.0990544746,0.5237383707\C,-4.4925944441,0.742296791,
0.4357790998\C,-5.0631306713,1.3117779939,1.5551515583\C,-4.5413321254,
1.0275196883,2.810008441\C,-3.4325382339,0.2056030178,2.9141033709\C,-
2.8248575112,-0.3459260464,1.7911042914\O,-1.3441983074,2.5824189624,
0.0068094178\O,-0.4588308117,3.5837721162,-2.0864869679\H,-0.4556274226,
1.6418724367,-5.0976186024\H,0.7723274117,0.7087701287,-7.020238547\H,-
0.0165005485,-1.3731987036,-8.1106261712\H,-2.0561939814,-2.4969207768,-
7.2609040681\H,-3.2805320549,-1.5670870273,-5.3597334967\H,-4.5065888313,
1.012504193,-2.9443464668\H,-4.5707605699,-0.2787443576,-4.2560833412\H,-
3.7958637889,7.1856906342,-2.7851968067\H,-2.0793104042,5.4125868049,-
2.9462375053\H,-3.62937621,3.5917702815,0.6058554482\H,-5.3473222171,
5.3657141535,0.7640936446\H,-6.1266237738,7.5660862751,0.0888364709\H,-
5.3779100832,8.411241893,-1.2785097087\H,-6.6064732506,7.183292256,-
1.563898739\H,-4.9881746415,1.4528764686,3.701194961\H,-5.9134222085,
1.9750974234,1.4486664765\H,-4.8969536798,0.9613577994,-0.5423219779\C,-
1.5573951798,-1.1147594562,1.9466369911\H,-3.0100071657,-0.0087447293,
3.88974409\C,-0.4353587508,-0.3679534286,1.2389890167\H,1.759517288,-
0.484545928,-0.1393927938\H,2.154372119,-1.0877906735,-2.4888191767\
H,0.2182550351,-1.74897677,-3.9223005356\H,-2.040125638,-1.6799606177,-
2.9821130622\H,0.526649815,-0.6391771752,1.6790403673\H,-0.5607369352,
0.7087346238,1.3962678745\H,-1.3264472621,-1.2140551638,3.0081788922\H,-
1.6468666005,-2.1270759546,1.5391538873\\Version=ES64L-G16RevB.01\HF=-
2213.786918\RMSD=5.607e-09\RMSF=9.481e-07\Dipole=-1.4155313,0.3185062,
0.2812759\Quadrupole=4.7126272,-1.9742129,-2.7384143,-26.2679964,
0.2949862,3.5131805\PG=C01 [X(C30H26N2O2S2)]\\@

10n (from 1n and 7a)

HF=-2213.8343802 a.u (0)

1\1\GINC-R10N10\FOpt\RPBE1PBE\def2TZVP\C30H26N2O2S2\
WURTHWE\10-Feb-2021\0\\# pbe1pbe/def2tzvp Opt=readfc geom=check
guess=read Pop=NBO Freq emp=gd3bj nosym scrf=(solvent=dichloromethane)
\\Product 10n (NNS)\\0,1\C,-0.0259896739,-0.1010202247,-0.076071345\C,-
0.0297775488,-0.1583750366,1.3301943226\C,1.2005825559,-0.2869938145,
1.9859231479\C,2.4052760505,-0.3419326703,1.3111176393\C,2.4113130099,-
0.2569821676,-0.0705217406\C,1.2068856721,-0.142672249,-0.7339045196\C,-
1.3039147935,-0.0740149445,2.1726705949\C,-1.0291798118,-0.2153821844,
3.6727213602\C,-2.2054799645,0.1225274167,4.555392466\N,-3.1961444755,

0.867725194,4.2378217918\N,-3.2560974814,1.41379372,3.0022513416\S,-1.8337955551,1.6928378434,2.0834700268\C,-2.2450814348,-0.4596798812,5.9114741526\C,-3.4514558061,-0.5317960385,6.6127570681\C,-3.4944318194,-1.0710457038,7.8849831589\C,-2.3333916611,-1.5451449503,8.4855430988\C,-1.1314347801,-1.4782646912,7.7988861275\C,-1.0873315969,-0.9451022811,6.5186507552\S,-4.6045786931,2.3750350327,2.7482361012\C,-4.3286022918,3.7813061531,3.750025944\C,-4.719582157,3.7588569905,5.0828209998\C,-4.4379265188,4.8511913388,5.8828422656\C,-3.7725816585,5.9662395054,5.3740992944\C,-3.3888628977,5.9597084556,4.0340655402\C,-3.6589820328,4.8754326243,3.2180413247\C,-3.5017414222,7.1533785421,6.2425679876\C,-2.403453215,-1.0289379165,1.714976777\C,-3.028967736,-0.909965807,0.4640991124\C,-4.0393878275,-1.8034344501,0.1221936094\C,-4.4437055872,-2.8156023712,0.9759479012\C,-3.8187688544,-2.946075341,2.2026689388\C,-2.816473989,-2.0583396066,2.5606307011\O,-4.5367091516,2.7494089948,1.3686865841\O,-5.72662296,1.654033041,3.260697889\H,-4.3534242591,-0.16588038,6.1386387185\H,-4.4399799512,-1.128371305,8.4118396422\H,-2.3689134446,-1.9681632674,9.4827093594\H,-0.2202132327,-1.8426802305,8.2586666533\H,-0.1359671832,-0.896258737,6.0031753695\H,-0.2137690056,0.4534202198,3.9469229922\H,-0.6915015372,-1.2268378082,3.9081653773\H,-4.7399784278,4.8394214161,6.9242813161\H,-5.2371355169,2.8954830836,5.4798030631\H,-3.3627511623,4.8768870321,2.1765698468\H,-2.8699609668,6.8184585216,3.6228962909\H,-2.6502881157,7.7280768555,5.8764015609\H,-4.3689230764,7.8206926676,6.2567499705\H,-3.3050543232,6.8534076781,7.2729868135\H,-5.236361467,-3.4941731812,0.6829644054\H,-4.517315394,-1.6936892854,-0.8455200688\C,-2.6235089511,0.1242720382,-0.5347036414\H,-2.3599071503,-2.1935528203,3.5306380205\H,-4.107898812,-3.7310589416,2.8914088667\H,1.23646155,-0.3599525306,3.0622625104\H,3.3289720588,-0.4464239505,1.867671583\H,3.340992902,-0.2860340859,-0.6267216341\H,1.202728047,-0.0911700241,-1.81815871\C,-1.2011647374,-0.0606381585,-1.030301069\H,-3.2823967593,0.0454676713,-1.4023549315\H,-2.7619481952,1.1249414752,-0.126717896\H,-0.9910509876,0.7208367812,-1.7667971433\H,-1.1642534031,-1.0032113966,-1.5896203279\\Version=ES64L-G16RevB.01\HF=-2213.8343802\RMSD=4.984e-09\RMSF=7.855e-07\Dipole=2.2372081,0.3405148,1.2401726\Quadrupole=-32.7400991,8.5172046,24.2228946,1.602744,1.4266541,10.4193469\PG=C01 [X(C30H26N2O2S2)]\\@

TS for 10n

HF = -2213.7509673 a.u (1, -264.9009cm^{-1})

1\1\GINC-R07N17\FTS\RPBE1PBE\def2TZVP\C30H26N2O2S2\WURTHWE\15-Feb-2021\0\\# pbe1pbe/def2tzvp opt=(ts,noeigentest,readfc,maxstep=5) geom=check guess=read scrf=(solvent= dichloromethane) freq pop=nbo emp=gd3bj nosym\\TS for 10n\\0,1\C,0.0825161352,-0.0294487112,

0.1915746208\C,0.2807951289,-0.3255295908,1.5333551028\C,1.5315853072,-
0.8169564969,1.940785889\C,2.5637801846,-0.8978999006,1.0148195746\
C,2.3744041425,-0.5303822576,-0.3088411851\C,1.1199905477,-0.1183913109,-
0.7244114228\C,-0.7737430025,-0.2346827877,2.5903362775\S,-0.701211423,
0.921978075,3.7848845968\C,1.6848160745,-1.4000599496,3.3076700967\
C,0.8709628021,-2.6851700469,3.4206634006\C,-0.6405678229,-2.6320375226,
3.3238631539\C,-1.4343813228,-1.5254549075,2.9677611152\C,-2.8250942473,-
1.6436947337,3.0329316021\C,-3.4416010523,-2.8276544533,3.3876193649\C,-
2.6658834959,-3.9325890369,3.7042242121\C,-1.2900350861,-3.8160493088
,3.6789942382\C,-2.6197457526,1.0864227971,1.4365943059\C,-2.0074695776,
2.2064746534,0.9342616829\C,-1.8790714462,2.4998760537,-0.5017283765\C,-
0.7889002526,3.2265435862,-0.9821492161\C,-0.6625510928,3.4871749264,-
2.3360389662\C,-1.6180722356,3.022140468,-3.2300753877\C,-2.7067373714,
2.3003279995,-2.7605451411\C,-2.8389623757,2.0444396504,-1.4052312431\N,-
1.3659945806,3.0715495698,1.7575729483\N,-1.4265557418,2.834751298,
3.0027364566\S,-0.4197831223,3.9618680618,3.8748298568\O,0.8680747742,
4.0697798046,3.2554399043\C,-1.3107713533,5.4450842906,3.6277928986\C,-
0.9640439494,6.2851680816,2.5777925366\C,-1.6918519772,7.4437207751,
2.3806093486\C,-2.761267443,7.7740166179,3.2135729713\C,-3.0893579749,
6.9085555822,4.2561296512\C,-2.3733246049,5.74445405,4.4697364506\C,-
3.5222460672,9.0442901969,3.0065587157\O,-0.5394782368,3.5501764262,
5.2429940749\H,-0.034349639,3.5653484162,-0.2838050065\H,0.1926122555,
4.0463734967,-2.697519448\H,-1.5156163551,3.223499503,-4.2900545552\H,-
3.4624235535,1.9443787951,-3.4509374116\H,-3.7063183252,1.5043297007,-
1.0435528491\H,-2.9516378214,1.0977365758,2.4621000092\H,-3.0173634172,
0.3252752261,0.778322126\H,-1.4276214436,8.1059729656,1.5636761615\H,-
0.1347269914,6.0288148986,1.9316891734\H,-2.6273140309,5.0756134535,
5.2822779322\H,-3.9190995221,7.1512166829,4.9104651199\H,-3.5797168442,
9.3035094428,1.9483863084\H,-4.5343209004,8.9712127906,3.4060478114\H,-
3.0239564494,9.872864297,3.5191494053\H,3.1958190078,-0.5900778008,-
1.0133874323\H,3.5283361879,-1.2732109498,1.3405481445\H,-0.8912312555,
0.2753426253,-0.1550844628\H,0.9382276556,0.1401401743,-1.7610344869\H,-
3.4444574776,-0.7926887637,2.7952637723\H,-4.5234057803,-2.883325561,
3.4153812217\H,-3.1282596482,-4.8718706066,3.9840599939\H,-0.6783339064,-
4.6675895376,3.9607378685\H,2.7333907956,-1.6466691293,3.4849114626\
H,1.3914409606,-0.6896051263,4.0837334122\H,1.1075908242,-3.159937545,
4.3775147077\H,1.2284514805,-3.3788895385,2.6505047934\\Version=ES64L-
G16RevB.01\HF=-2213.7509673\RMSD=4.186e-09\RMSF=6.778e-07\Dipole=-
1.7344252,0.051883,-1.9357272\Quadrupole=4.2829266,21.3148587,-25.5977853,
-20.0541323,-8.1976797,-12.6094287\PG=C01[X(C30H26N2O2S2)]\\@

1i
HF=-785.0426912 a.u. (0)
1\1\GINC-R08N11\FOpt\RPBE1PBE\def2TZVP\C8H12O1S1\
WURTHWE\14-Feb-2020\0\\# pbe1pbe/def2tzvp Opt=readfc geom=check
guess=read scrf=(solvent=dichloromethane) Pop=NBO Freq emp=gd3bj
nosym\\1i Educt\\0,1\C,0.0872732654,-0.0000973704,-0.1136745034\C,
0.1785190402,-0.0000043865,1.4195276857\C,1.7145342261,-0.0001077294,
1.4138201545\C,1.6122855168,-0.000187838,-0.1076296596\O,-0.6399140115,
0.000108923,2.2914832111\C,2.3433417753,-1.2603116979,1.9942005467\
C,2.3435153024,1.2600740773,1.9940589892\S,2.7094145502,-0.0003176702, -
1.2767033248\C,-0.5313203581,1.2601714975,-0.7047742657\C,-0.5314626531,-
1.2603774482,-0.7046037702\H,3.4101071314,-1.2727339757,1.7617629793\
H,2.2179385805,-1.2663758874,3.078521009\H,1.8921179803,-2.1666717516,
1.587217124\H,3.4102812088,1.2723262052,1.7616146334\H,1.8924123664,
2.1664505184,1.5869785123\H,2.218117729,1.2662745157,3.078379237\H,-
0.3611287383,1.2755567912,-1.7831899567\H,-1.6065556865,1.2635304182,-
0.5167696985\H,-0.1010785595,2.1664229399,-0.2754156862\H,-0.3612795644,-
1.2759244716,-1.7830184078\H,-0.1013165921,-2.1666186868,-0.2751274796\H,-
1.6066973268,-1.2635937109,-0.5165924632\\Version=ES64L-G16RevB.01\HF=-
785.0426912\RMSD=5.583e-09\RMSF=5.540e-06\Dipole=0.0365281,-
0.0000268,-0.0378073\Quadrupole=-1.5444807,4.3564253,-
2.8119447,0.0008388,8.2509848,-0.0009799\PG=C01[X(C8H12O1S1)]\\@

9q (from 1i and 7a)
HF=-2022.4563956 a.u. (0)
1\1\GINC-R08N11\FOpt\RPBE1PBE\def2TZVP\C23H26N2O3S2\
WURTHWE\15-Feb-2020\0\\# pbe1pbe/def2tzvp Opt=(maxstep=8,readfc)
geom=check guess=read Pop=NBO Freq emp=gd3bj nosym scrf=(solvent=
dichloromethane)\\9q NNC-Product\\0,1\C,-0.0955905159,0.6179846118, -
0.0600441658\C,-0.0706783505,-0.8330422579,0.3905052888\O,0.7902420676,-
1.6669060695,0.3845991412\C,-1.6726787345,0.5024495183,0.0016182803\N,-
2.431202272,1.4838432542,0.7398886606\N,-3.7333580338,1.1060797579,
1.1182291147\C,-4.4257523543,0.4274843859,0.2801556353\C,-4.0694983295,
0.0473151343,-1.1273039017\S,-2.3642090229,0.2978723323,-1.6607008065\C,-
5.7685523649,0.0220771917,0.7449282033\C,-6.0439763639,-0.0240754124,
2.1132162336\C,-7.3032588144,-0.3758693282,2.5642437671\C,-8.3083770456,-
0.6878666188,1.6573168919\C,-8.0437202343,-0.6483672754,0.2965690887\C,-
6.7813090367,-0.3014111015,-0.1584009671\S,-2.4350495852,3.1284492017,
0.3804262611\C,-3.7718347743,3.4004693487,-0.7252189274\C,-3.5250487063,
3.4715696925,-2.0890659492\C,-4.5886112277,3.6385013489,-2.9589279752\C,-
5.8974155306,3.729403086,-2.4883067006\C,-6.1173152756,3.6650249944,-
1.1121082096\C,-5.066027435,3.5069077095,-0.2288479108\C,-7.0409236416,

3.9202616671,-3.4328341136\C,-1.531739374,-0.873516777,0.8018144296\O,-2.7413093146,3.7901509313,1.6119924944\O,-1.2094404567,3.4061611294,-0.3058891884\H,-5.2549632181,0.2166708948,2.8136686859\H,-7.5013296089,-0.4129381969,3.6290102955\H,-9.2942803715,-0.9652033884,2.0116721565\H,-8.8228795822,-0.888050111,-0.4172134631\H,-6.6009356345,-0.2680215237,-1.2253329901\H,-4.3606117116,-0.9897208198,-1.3003092665\H,-4.6886407747,0.6685812122,-1.7831720616\H,-4.3997581248,3.6937781634,-4.0252378723\H,-2.5118824246,3.3891796235,-2.4578006624\H,-5.2417596135,3.4514642014,0.8375484601\H,-7.1288470834,3.734457432,-0.7277737305\H,-7.2968653252,4.9812753365,-3.5110696947\H,-6.7937907238,3.5659922063,-4.434127971\H,-7.9327902283,3.3964641485,-3.0849212437\C,0.5471399798,0.8812914163,-1.4074763596\C,0.5739949211,1.4871979186,1.0092002716\C,-2.2827219243,-2.1074323948,0.3439116349\C,-1.6587338837,-0.7412750414,2.3200742128\H,-1.2528963599,-1.6437372407,2.7812325414\H,-1.1311723761,0.1181785783,2.7260282411\H,-2.7097647172,-0.6534759404,2.5978529614\H,-1.7889684208,-2.9853842868,0.7656395578\H,-3.3125922478,-2.102803909,0.7070692563\H,-2.2807282151,-2.2084527927,-0.7412973217\H,1.6267981492,0.7481208722,-1.3143234514\H,0.1955837072,0.194431444,-2.1796060897\H,0.3499463249,1.9064774576,-1.7221453309\H,1.5565237503,1.0634146269,1.2281197019\H,0.7042406368,2.5001184339,0.6363854329\H,0.0074564883,1.5402921505,1.9363491135\\Version=ES64L-G16RevB.01\HF=-2022.4563956\RMSD=2.388e-09\RMSF=7.126e-07\Dipole=-3.5913399,-0.7791846,-1.6159389\Quadrupole=37.0747269,-30.6832701,-6.3914568,2.3261389,14.0222959,-13.4765874\PG=C01 [X(C23H26N2O3S2)]\\@

1i + 7a vdW-complex
HF=-2022.4029297 a.u (0)
1\1\GINC-R09N10\FOpt\RPBE1PBE\def2TZVP\C23H26N2O3S2\WURTHWE\25-Feb-2021\0\\# pbe1pbe/def2tzvp opt=(maxstep=9) freq pop=nbo emp=gd3bj nosym scrf=(solvent= dichloromethane)\\ 1i + 7a vdW-complex \\0,1\C,-0.3659475506,0.0574479337,0.0306313998\C,-0.1911805496,0.2216414584,1.4041283961\C,1.0849092379,0.0686926544,1.9497880102\C,2.163796109,-0.2258551261,1.1313612133\C,1.9838008316,-0.3793214808,-0.2359222554\C,0.7152363929,-0.2360631129,-0.7830976758\C,-1.3478616828,0.5094236298,2.2658380539\C,-2.4246491904,1.2239751564,1.9362814418\N,-1.1776050711,0.0286044192,3.5882930168\N,-2.174364796,-0.421672791,4.1380496298\S,-1.8840019266,-0.9540549712,5.7711646072\O,-2.727781395,-0.1109319645,6.5663304836\C,-0.2023849045,-0.6380020015,6.1098809961\C,0.1715757783,0.6116097589,6.5869353008\C,1.5134892755,0.8799590285,6.7818241441\C,2.48788604,-0.0783365008,6.5020637499\C,2.0832536851,-1.3231780415,6.0214686804\C,0.7456560659,-1.610190664,5.8208241592\C,3.9344706964,0.215629259,6.7380809396\O,-2.1215555564,-2.3683610874,

5.7168038398\S,-3.2992917264,-2.2688491163,0.9335810909\C,-2.6560349462,-3.6276104719,1.4883759173\C,-3.2612330104,-4.8876571854,2.0993611214\C,-1.8345781551,-5.4550422714,2.0961469537\O,-1.3716455273,-6.5205678476,2.3820245639\C,-1.2249349751,-4.1504054234,1.5625042898\C,-0.3857908571,-3.4392378804,2.6212280557\C,-0.4917301033,-4.2392292718,0.2351754444\C,-3.85561757,-4.6758533212,3.4872761568\C,-4.2256615438,-5.6236465134,1.1800451171\H,1.226537436,0.1809608327,3.0173953685\H,3.1502882862,-0.3382899551,1.5660018453\H,2.8272944073,-0.6175932774,-0.8732671315\H,0.5643012364,-0.3692970681,-1.8479924868\H,-1.3602201494,0.1261718928,-0.3931660206\H,-3.2180511227,1.3842828218,2.6547750291\H,-2.5056791156,1.6938836335,0.9653691932\H,2.8289251802,-2.0779524919,5.7984638385\H,0.436396839,-2.5777547578,5.4475705594\H,-0.5818939663,1.3589632131,6.8011233281\H,1.8133872703,1.8528029859,7.1547894055\H,4.2054653362,-0.021454428,7.7714684743\H,4.5727875118,-0.3816516642,6.086023189\H,4.1558472995,1.271539881,6.5768401615\H,-4.0920540332,-5.6431198505,3.9349585329\H,-3.1773298363,-4.137657595,4.1504993814\H,-4.7760678444,-4.0948928793,3.3981121824\H,-4.4834813576,-6.5906711799,1.6159692735\H,-5.1372475769,-5.0336611728,1.0649932203\H,-3.7969792257,-5.7913671828,0.1903595789\H,0.4484468598,-4.7781612583,0.3675562592\H,-1.0848782869,-4.7541867748,-0.5223070989\H,-0.2697701205,-3.2315258554,-0.1235779352\H,0.5332132164,-4.0033113335,2.7934554898\H,-0.1239749269,-2.4410477522,2.2670496469\H,-0.920892,-3.3444211313,3.5670076164\\Version=ES64L-G16RevB.01\HF=-2022.4029297\RMSD=7.295e-09\RMSF=2.959e-06\Dipole=2.7840968,1.4811968,-1.0515384\Quadrupole=10.6478831,-8.856214,-1.791669,-4.0958863,33.2380452,16.3447583\PG=C01[X(C23H26N2O3S2)]\\@

TS1 for zwitterionic intermediate Z for 9q

HF=-2022.3926436 a.u.(1, -214.2616cm^{-1})

1\1\GINC-R08N33\FTS\RPBE1PBE\def2TZVP\C23H26N2O3S2\WURTHWE\08-Mar-2021 \0\\# pbe1pbe/def2tzvp opt=(ts,noeigentest,calcfc,maxstep=6) freq pop= nbo emp=gd3bj nosym scrf=(solvent=dichloromethane)\\ TS1 for zwitterionic intermediate Z for 9q \\0,1\C,-0.0289386837,0.1557780445,-0.0220291722\C,-0.0156276805,0.0562964413,1.3744100637\C,1.2238597291,0.0001162567,2.018217604\C,2.4045186255,0.0387124653,1.2944225302\C,2.3778300349,0.1320202085,-0.0898252278\C,1.1529217024,0.1896455467,-0.7414670514\C,-1.2746411067,0.0304800749,2.1299794558\C,-1.3574732985,-0.0221207301,3.5129439394\N,-2.410132941,0.129786665,1.3710237013\N,-3.5053459039,0.2723664896,1.9626309728\S,-4.836920099,0.262011919,0.9053612107\O,-5.3434185038,-1.0841325397,0.9106903023\C,-4.2215626646,0.6529432296,-0.6862800574\C,-4.0646641939,1.9844663034,-1.0452132337\C,-3.5021688174,2.2895573082,-2.2715948568\C,-3.0889475854,1.2834438328,-3.1441527535\C,-3.2575115086,-

0.0456290943,-2.7578574086\C,-3.8179244953,-0.36821229,-1.5350142791\C,-2.5005052941,1.6219196827,-4.4767197733\O,-5.6872228397,1.3226649548,1.3752614038\S,-1.258897507,2.2197801082,3.890443179\C,-2.6833405282,2.847771045,4.2991004609\C,-3.7697237952,2.5156100726,5.3165784893\C,-4.6609730725,1.3186196632,5.0125334368\C,-3.3375309759,4.1504761646,3.8491522599\C,-3.8761988155,4.0933779287,2.4222436844\C,-2.5152224822,5.4077350053,4.0885377978\C,-4.4025671971,3.858353966,4.9149691218\O,-5.3603150118,4.4576754526,5.3038147282\C,-3.2573000094,2.4797505945,6.7523553514\H,-0.9794425779,0.2077502282,-0.5369850745\H,1.1160076282,0.2654175271,-1.8224824536\H,3.3022412294,0.1612680934,-0.6545442804\H,3.3524492793,-0.0052239161,1.81834876\H,1.2807202165,-0.0727472107,3.097405701\H,-2.3248645878,-0.2129387341,3.9567972372\H,-0.499261765,-0.2897081381,4.1115241671\H,-3.3776088138,3.3281405642,-2.5573953416\H,-4.3818932834,2.7695177463,-0.3705322186\H,-3.9404915552,-1.4005974042,-1.2336635638\H,-2.937819294,-0.8395972834,-3.4236787796\H,-3.285929046,1.6784915764,-5.2366236664\H,-1.9963391049,2.5888884874,-4.4540176941\H,-1.7877035638,0.8619149607,-4.799822548\H,-5.5395417869,1.3662418422,5.6590924694\H,-4.9848830022,1.3008618567,3.9729972596\H,-4.1303733278,0.3890312764,5.2256166757\H,-4.105427871,2.453663906,7.4390918655\H,-2.6558948327,1.5808908709,6.9026374639\H,-2.6431954131,3.3498231351,6.9896086462\H,-3.1359886106,6.2885064229,3.914669096\H,-2.1257715657,5.4515429361,5.1069134783\H,-1.6723520409,5.4325049979,3.3946172598\H,-4.4697840484,4.9881188652,2.224243932\H,-3.0358733013,4.0660890431,1.7247500529\H,-4.4977575478,3.2138769575,2.2532510245\\Version=ES64L-G16RevB.01\HF=-2022.3926436\RMSD=3.792e-09\RMSF=1.220e-06\Dipole=3.1835734,0.8323929,-0.4054359\Quadrupole=-40.8176163, 7.3823372, 33.4352792,2.5626379,17.5241849,-5.0687835\PG=C01[X(C23H26N2O3S2)]\\@

Zwitterionic intermediate Z for 9q

HF=-2022.403018 a.u (0)

1\1\GINC-R08N08\FOpt\RPBE1PBE\def2TZVP\C23H26N2O3S2\WURTHWE\08-Mar-2021\0\\# pbe1pbe/def2tzvp opt=(maxstep=8) freq pop=nbo emp=gd3bj nosym scrf=(solvent=dichloro methane)\\ zwitterionic Z intermediate for 9q \\0,1\C,0.2241311216,-0.2845436396,0.1860900077\C,0.1718881995,-0.1396020681,1.580138702\C,1.367847311,-0.2544976798,2.2949663881\C,2.5662608183,-0.5133366915,1.645320864\C,2.600434209,-0.6634327006,0.2683023699\C,1.4183381812,-0.5476095398,-0.4562158878\C,-1.1075751202,0.1269773546,2.2409650834\C,-1.1814847584,0.5875190003,3.6464511286\N,-2.2018621999,0.0497267685,1.5210757987\N,-3.3314016811,0.3992257353,2.0779556533\S,-4.592625888,0.0990457833,1.0595097932\O,-4.7966901939,-1.323955924,0.9195168759\C,-4.1461245587,0.7237423182,-0.5271250426\C,-

4.4673141706,2.0291408698,-0.8674223497\C,-4.0680078316,2.5325360996,-2.0946662446\C,-3.3433247589,1.7507182409,-2.9912296459\C,-3.0344617827,0.4399720636,-2.6286534226\C,-3.4324251649,-0.0779353367,-1.4090030042\C,-2.9321752468,2.2909250329,-4.3249003693\O,-5.6918154107,0.885856802,1.568755769\S,-1.2940686796,2.4010741207,3.3803173459\C,-2.7693431608,2.8483809857,3.878814983\C,-3.7297904395,2.4482440303,4.9977150115\C,-4.7106234205,1.3026598089,4.7953931682\C,-3.4638434841,4.1596517503,3.5701352955\C,-4.2638818243,4.061984921,2.2648165301\C,-2.639430569,5.4320336056,3.6332065994\C,-4.3560677493,3.837787133,4.7771726356\O,-5.2021988499,4.4477746985,5.355108792\C,-3.0200554146,2.3411686538,6.348247011\H,-0.6929484524,-0.1811021067,-0.3797221903\H,1.4316862957,-0.6552472886,-1.535115897\H,3.5370406656,-0.8645651789,-0.2382666956\H,3.4779800215,-0.6007251609,2.2251934683\H,1.379552025,-0.1549374053,3.3732068201\H,-2.095475065,0.2676461182,4.1392757141\H,-0.3089991954,0.389767216,4.2595900347\H,-4.3242824844,3.5521580988,-2.3617919813\H,-5.0367420833,2.6397326429,-0.1789089803\H,-3.1953757229,-1.097366486,-1.1347643658\H,-2.4725644276,-0.1845352112,-3.3151091707\H,-3.655161625,2.0044488068,-5.0947939636\H,-2.8782244803,3.3802823531,-4.3132097208\H,-1.9609847579,1.8968703292,-4.6292673996\H,-5.4729219521,1.3848033025,5.5730657396\H,-5.1841800428,1.3315248764,3.8169669448\H,-4.2119031839,0.3392801756,4.9009163355\H,-3.7740125949,2.3469856479,7.1372037662\H,-2.4643652562,1.4035957162,6.4063325272\H,-2.3295384698,3.1679390827,6.5210371499\H,-3.3060608052,6.2944031736,3.5845011006\H,-2.0577859839,5.4931567152,4.5539272093\H,-1.9556814139,5.4807785389,2.7830239474\H,-4.9341458512,4.9209101299,2.2003996113\H,-3.5707300095,4.0824013758,1.4221027483\H,-4.8492061384,3.1435557178,2.2097827567\\Version=ES64L-G16RevB.01\HF=-2022.403018\RMSD=6.608e-09\RMSF=3.921e-06\Dipole=3.5402853,2.6993474,0.9847448\Quadrupole=-45.1662667,10.4823557,34.683911,-10.6033122,13.9608596,4.6191774\PG=C01[X(C23H26N2O3S2)]\\@

TS2 for zwitterionic intermediate Z for 9q

HF=-2022.4017695 a.u. (1, -67.9594cm^{-1})

1\1\GINC-R08N07\FTS\RPBE1PBE\def2TZVP\C23H26N2O3S2\WURTHWE\09-Mar-2021\0\\# pbe1pbe/def2tzvp opt=(ts,noeigentest, calcfc,maxstep=8) freq pop= nbo emp=gd3bj nosym scrf=(solvent= dichloromethane)\\ TS2 for zwitterionic intermediate Z for 9q \\0,1\C,0.0015615338,-0.0254382252,0.0065343091\C,0.0021954345,0.0094908559,1.4029771946\C,1.2353222778,0.0499160322,2.0652715066\C,2.41986879,0.0700437448,1.3532071272\C,2.4059955639,0.0394504897,-0.0368295444\C,1.1918737129,-0.0091442707,-0.7046709262\C,-1.2449580772,0.0236994719,2.1740990224\C,-2.5139681211,-0.4866652631,1.6169219423\S,-2.5622267017,-2.1058802845,2.4735792325\C,-3.5341055788,-1.7926977236,

3.7637621598\C,-4.9725908326,-1.2418344573,3.8551767177\C,-5.2783856982,0.2032335966,4.2015134121\N,-1.1790277467,0.4044620655,3.4246166971\N,-2.274462605,0.3656385563,4.1501322484\S,-1.9812734936,1.1523086363,5.5899367877\O,-1.9232962275,2.5656853672,5.3079658866\C,-0.3934212343,0.6732476658,6.1836285909\C,0.745798686,1.2866001487,5.6723533444\C,1.9905520945,0.8888022164,6.121574853\C,2.1248817449,-0.1119558161,7.08548185\C,0.9704718784,-0.6961535156,7.5973568457\C,-0.2849851301,-0.3127953373,7.1511295883\C,3.4809350972,-0.5366507063,7.5540140924\O,-2.9839579685,0.6977332652,6.5218548525\C,-3.7205474939,-2.7886583596,4.909159509\C,-3.6735670648,-4.2570302164,4.4954224669\C,-5.1400556274,-2.2209012752,5.0264762627\O,-6.0524522792,-2.4573006697,5.7597813055\C,-2.8646472727,-2.5487012032,6.1442441999\C,-5.8053476632,-1.6888982487,2.6482229505\H,1.2433515062,0.0605325749,3.1477463098\H,3.3639646958,0.0992825484,1.8854660872\H,3.3357160635,0.0490055498,-0.59354821\H,1.1671694692,-0.0302080328,-1.7880491314\H,-0.9317661321,-0.0434975126,-0.5430096862\H,-3.3840516654,0.0892585003,1.923244648\H,-2.5197292833,-0.6711184107,0.5484038641\H,1.0527530615,-1.4607526476,8.3620528801\H,-1.1758088022,-0.763492145,7.5667814988\H,0.6480517292,2.070706208,4.934109018\H,2.8784237125,1.3666060401,5.7209753439\H,4.0629829521,0.3215510077,7.8983030599\H,3.4127492503,-1.2555029808,8.3707749916\H,4.0441223361,-1.0005366226,6.7395211072\H,-6.3534815034,0.2788063065,4.3787253962\H,-4.7479592284,0.5272464526,5.0920814997\H,-5.0166525417,0.86697615,3.3774295857\H,-6.8622305099,-1.5472648137,2.8797829446\H,-5.5569746118,-1.0791313464,1.7774163809\H,-5.6422195651,-2.7369215848,2.394194798\H,-4.045208694,-4.8695831981,5.3185153378\H,-4.2813633261,-4.456475001,3.611728314\H,-2.6437603153,-4.5547283602,4.2856713756\H,-3.1756834228,-3.2392483655,6.9301985494\H,-1.8165941155,-2.7380061047,5.904081708\H,-2.9691788555,-1.5273778648,6.502381812\\Version=ES64L-G16RevB.01\HF=-2022.4017695\RMSD=5.567e-09\RMSF=7.490e-07\Dipole=1.3792324,-2.9328151,-2.6242571\Quadrupole=12.3477107,7.2039798,-19.5516905,10.6075505,38.2498962,-26.2548119\PG=C01 [X(C23H26N2O3S2)]\\@

10q (from 1i and 7a)

HF=-2022.4613472 a.u. (0)

1\1\GINC-R08N28\FOpt\RPBE1PBE\def2TZVP\C23H26N2O3S2\WURTHWE\ 16-Feb-202 0\0\\# pbe1pbe/def2tzvp Opt=(maxstep=8,readfc) geom=check guess=read Pop=NBO Freq emp=gd3bj nosym scrf=(solvent=dichloromethane) \\ 10q NNS-Product\\0,1\C,0.1963029522,-0.048644251,0.4424462251\C,-0.9660857791,0.310979988,1.1183812972\C,-0.9707098434,1.3596244964,2.025222932\C,0.205210784,2.0559588477,2.2544092895\C,1.3862372443,1.7073

520676,1.6061275161\C,1.3614105156,0.6472926005,0.6962804683\S,-2.426328395,-0.6124962312,0.857418433\O,-3.4918206082,-0.0200702284,1.6083425633\C,2.6626662928,2.4349613349,1.883144789\N,-2.1742917606,-2.1022493348,1.6012461088\S,-2.4283374812,-2.1549777955,3.3195948637\C,-1.0530860379,-3.2123077548,3.7859320152\C,-1.0550650725,-4.4072079676,2.8428357168\C,-0.9023962976,-4.004835045,1.4066899938\N,-1.4075626932,-2.9436123174,0.8978523782\C,-0.1074639018,-4.8467965063,0.4924763414\C,0.3410491976,-4.3397520426,-0.730600574\C,1.0883173047,-5.1235334738,-1.589287446\C,1.4007636039,-6.4353979432,-1.2498802227\C,0.9538297779,-6.9526404401,-0.0447512089\C,0.2082622048,-6.1650631546,0.8209707481\O,-2.5736607452,-0.8853741292,-0.5369226145\H,0.0932997843,-3.3204388487,-0.9974436076\H,1.4339317742,-4.7093119571,-2.529465216\H,1.9883028877,-7.0484663888,-1.9231971343\H,1.1829583425,-7.976377047,0.2272901395\H,-0.1406151724,-6.5983249005,1.7502639151\H,-1.9794496961,-4.9734107593,2.9809946707\H,-0.2379388901,-5.0775925163,3.1047248804\H,0.2056066276,2.8808191411,2.9578930174\H,-1.8849795567,1.6218211509,2.5410328282\H,0.1815850392,-0.8643030677,-0.2690477789\H,2.2714655783,0.3683919605,0.1767185478\H,3.1657473935,2.7132181008,0.9546760196\H,2.4876254222,3.3380096221,2.4678260992\H,3.35181511,1.7973219212,2.4443683888\C,-1.1641514985,-3.6208759334,5.3120575362\C,0.3808100031,-2.5918706144,4.0505780697\C,0.3122033842,-3.2590674416,5.41624437\O,1.1383890919,-3.4799661513,6.2561948036\C,-2.0253129734,-2.7032323365, 6.1792242154\C,-1.4958109058,-5.0669347168,5.6284827696\C,0.4268714444,-1.076356197,4.1968901649\C,1.5348378194,-3.0375511232,3.169167864\H,-1.3763509313,-5.2315345975,6.7009138465\H,-2.5339111332,-5.2867530438,5.3682711922\H,-0.8484182051,-5.7747882967,5.1111317575\H,-1.8235958091,-2.9175389488,7.2301952609\H,-1.8303054619,-1.6440583312,6.0114027655\H,-3.0851889368,-2.8949271997,5.9954118384\H,2.468805821,-2.6667678097,3.5957500542\H,1.6207710229,-4.1207093922,3.0841834339\H,1.4348848772,-2.617150687,2.1663254387\H,1.3614642487,-0.7940071534,4.6858006771\H,0.4027465641,-0.6110994304,3.2119441753\H,-0.4011835329,-0.67420921,4.7779984827\\Version=ES64L-G16RevB.01\HF=-2022.4613472\RMSD=6.796e-09\RMSF=1.921e-06\Dipole=2.2441014,-0.359812,0.9538492\Quadrupole=-15.5772539,19.4280143,-3.8507603,4.6139469,-12.5617994,0.186872\PG=C01 [X(C23H26N2O3S2)]\\@

TS for 9q

HF=-2022.3777236 a.u. (1, -270.4678 cm^{-1})

1\1\GINC-R08N39\FTS\RPBE1PBE\def2TZVP\C23H26N2O3S2\WURTHWE\08-Mar-2020 \0\\# pbe1pbe/def2tzvp Opt=(ts,noeigentest, readfc) geom=check guess=read freq pop=nbo emp=gd3bj nosym scrf=(solvent=dichloromethane)\\ TS for 9q \\0,1\C,0.1543957247,

0.0127801442,0.011184154\C,0.0445979859,-0.1132782885,1.3897719241\
C,1.1696384583,-0.2743197713,2.1885008843\C,2.4169805701,-0.3055905764,
1.5918686607\C,2.5572884562,-0.1797542243,0.2101249264\C,1.409810047,-
0.0251919627,-0.5673169178\S,-1.5270461534,0.0198556168,2.1396041853\O,-
2.5612197648,-0.3119595554,1.206141903\C,3.9081455094,-0.2368699628,-
0.4282046777\N,-1.749743036,1.7160368535,2.4698979724\N,-0.7043467638,
2.2024029029,2.9723263662\C,-0.6920354403,3.5452903394,3.2070136715\C,-
1.7380316906,4.3365926226,2.8094129571\C,0.5583014146,4.0600643348,
3.7848043368\C,0.5709435048,5.2594142515,4.4964666879\C,1.7534811312,
5.7537554408,5.0215728123\C,2.9394437128,5.0527233784,4.8509684199\
C,2.9340628397,3.8514614469,4.1547675478\C,1.754163599,3.357476677,3.62468
14771\O,-1.5008896616,-0.6040801879,3.4313507366\C,-1.7932863584,
4.2060262973,0.3864509591\C,-0.4905384036,4.7777751977,-0.2228054907\
C,0.2093067707,3.8616327993,-1.2103923201\S,-2.3005498765,2.6424002979,
0.3641665458\C,-2.6431509042,5.434938234,-0.0598173788\C,-3.8423221864,
5.083849995,-0.9246879178\C,-3.0318715917,6.492926928,0.9668911056\C,-
1.3936287119,5.845610156,-0.8444980712\O,-1.2031331033,6.6840394946,-
1.6782516373\C,0.5258838827,5.4155969827,0.7172293115\H,1.7506763829,
2.4294898596,3.0668915077\H,3.8565616657,3.2994478743,4.0167488001\
H,3.8639277821,5.439335725,5.2636224535\H,1.7469282558,6.6847698613,
5.576087152\H,-0.3538837273,5.7998616601,4.6615996846\H,-2.7184541153,
3.9057216153,2.674935054\H,-1.6690632664,5.4074125584,2.921436408\
H,1.5031091406,0.0720199972,-1.6432156434\H,-0.7333063998,0.1443666062,-
0.5929766592\H,1.0615173858,-0.3711820886,3.2606749628\H,3.2998707674,-
0.4269392054,2.209600579\H,4.6827100502,0.1261600705,0.2485369497\
H,4.1600439397,-1.2685006974,-0.6924718953\H,3.9384270763,0.3522248297,-
1.3457569347\H,-3.489879432,7.3341662999,0.4434564452\H,-3.7626525499,
6.0901084248,1.670706802\H,-2.1779240857,6.8780794766,1.5237150673\H,-
4.2354410856,5.9889680624,-1.3913633457\H,-3.5787369827,4.3709898334,-
1.7076154638\H,-4.6277047089,4.6397602554,-0.3099379319\H,1.2191456814,
6.0198057358,0.1287223444\H,0.0646187032,6.0597713494,1.4653700848\
H,1.0951028299,4.6431475549,1.2340378209\H,0.9872541097,4.4150072912,-
1.7399077457\H,0.673968485,3.0297499884,-0.6765221959\H,-0.4879342251,
3.4474967109,-1.9401537301\\Version=ES64L-G16RevB.01\HF=-2022.3777236\
RMSD=9.033e-09\RMSF=6.714e-07\Dipole=2.5977032,1.9916079,0.0491534\
Quadrupole=3.4290743,5.1399416,-8.5690159,-7.2158341,7.8784068,27.5120413\
PG=C01[X(C23H26N2O3S2)]\\@

11a

HF=-1202.6606138 a.u (0)

1\1\GINC-R09N16\FOpt\RPBE1PBE\def2TZVP\C16H18N2O1S1\
WURTHWE\15-Feb-2020\0\\# pbe1pbe/def2tzvp Opt=(maxstep=8,readfc)

geom=check guess=read Pop=NBO Freq emp=gd3bj nosym scrf=(solvent= dichloromethane)\\11a product after elimination\\0,1\N,-2.2308542831,-1.7656836403,2.2145857764\C,-1.7594510288,-2.7011593463,3.2436393109\S,0.0253164193,-2.5856728294,3.2386761334\C,0.216719055,-2.9562793518,1.5859664335\C,-0.8086553503,-2.806735599,0.7074596305\N,-1.8437772549,-1.9442875894,1.0494184301\C,-2.4022721241,-2.4492085777,4.6500778581\C,-3.5542644719,-1.4527716016,4.6426305398\C,-2.394664322,-4.1571316192,3.1842469872\C,-3.5565680764,-4.2989887985,2.2064046944\C,-0.7567319602,-3.2586357711,-0.6852442877\C,-1.4779676892,-2.6052328026,-1.6868416966\C,-1.4212514865,-3.0531274457,-2.9960387798\C,-0.6476068534,-4.1574223644,-3.3290993437\C,0.0640034742,-4.8187958414,-2.3378285328\C,0.0033684498,-4.3789024319,-1.0255183397\C,-2.8923110184,-3.8884713446,4.6005371354\O,-3.4465490007,-4.5853300782,5.4025251505\C,-1.4611490274,-2.1663590307,5.8092392251\C,-1.4729492452,-5.3595756594,3.0993728146\H,-2.0728003405,-1.7385379624,-1.4289784304\H,-1.982228757,-2.5329401789,-3.7640344297\H,-0.6064188067,-4.5055967469,-4.3545321338\H,0.658305368,-5.6910425553,-2.5843551045\H,0.5338189989,-4.9223844667,-0.2515059254\H,1.2139622147,-3.2309175252,1.2637377493\H,-4.1085923588,-5.2098631446,2.4455478852\H,-4.2487549901,-3.4578099189,2.2510491515\H,-3.1855884959,-4.38001445,1.1827004859\H,-2.0309153893,-6.2476259023,3.4037986944\H,-1.1203876884,-5.5078109936,2.0776408671\H,-0.6072582698,-5.263554096,3.755400814\H,-2.0235829156,-2.1982513865,6.7438765215\H,-0.6552596307,-2.8994190487,5.8812866622\H,-1.0210763611,-1.1713267165,5.7105374472\H,-4.0842585445,-1.5235079648,5.5943071556\H,-3.1714204515,-0.4375283786,4.5268027843\H,-4.2616988424,-1.6364867768,3.834626449\\Version=ES64L-G16RevB.01\HF=-1202.6606138\RMSD=5.862e-09\RMSF=1.475e-06\Dipole=1.1444287,-0.382703,-0.6216292\Quadrupole=-3.5533183,4.4433261,-0.8900078,-10.9119578,10.0066762,11.4132912\PG=C01[X(C16H18N2O1S1)]\\@

***p*-Tolyl sulfinic acid 12**

HF=-819.7609899 a.u (0)

1\1\GINC-R09N16\FOpt\RPBE1PBE\def2TZVP\C7H8O2S1\WURTHWE\15-Feb-2020\0\ \# pbe1pbe/def2tzvp Opt=(maxstep=8,readfc) geom=check guess=read Pop=N BO Freq emp=gd3bj nosym scrf=(solvent= dichloromethane)\\12: pTol-Sulfinic acid\\0,1\C,-0.0075143323,-0.1453753017,-0.1268213732\C,-0.0509764712,-0.3349947177,1.2493367372\C,1.1199325555,-0.2550758852,1.978650675\C,2.3424315096,0.0015083206,1.3531617243\C,2.3587632993,0.1717748581,-0.0275218028\C,1.191617028,0.0918170657,-0.7734373072\H,-0.9939528978,-0.5323169522,1.7467572375\H,1.0901866089,-0.394595506,3.0540337023\C,3.599983543,0.0943238805,2.1589252197\H,3.300464604,0.3660119977,-0.5293147894\H,1.199713315,

0.220669182,-1.849069481\S,-1.4896194071,-0.3239173212,-1.092899062\O,-2.245012295,0.9460037278,-0.3787344754\O,-1.1315137152,0.0499358583,-2.4655638099\H,-3.1977793453,0.8122262222,-0.4790439926\H,4.4780185693,0.1718059602,1.517415583\H,3.5780587206,0.9726834001,2.8100338059\H,3.7193477623,-0.7799847894,2.8030214173\\Version=ES64L-G16RevB.01\HF=-819.7609899\RMSD=3.994e-09\RMSF=1.565e-06\Dipole=0.2165909,-0.2928595,1.7205771\Quadrupole=11.0546291,-5.2740712,-5.7805579,0.6259801,-0.3715821,-0.3506783\PG=C01[X(C7H8O2S1)]\\@

TS for 1i+7a (NNC), Addition to C=O bond

HF=-2022.3688762 a.u. (1, -297.9983cm^{-1})

1\1\GINC-R08N27\FTS\RPBE1PBE\def2TZVP\C23H26N2O3S2\WURTHWE\12-Mar-2020\0\\# pbe1pbe/def2tzvp Opt=(ts,noeigentest,readfc) geom=check guess=read scrf=(solvent=dichloromethane) freq pop=nbo emp=gd3bj nosym\\TS for 1i+7a (NNC) (C=O-addition)\\0,1\C,-0.3156714279,-0.2369831923,0.2381909757\C,-0.248970395,-0.0280513028,1.6226719981\C,1.0178639869,0.1322247095,2.1932391264\C,2.1655995574,0.0911175181,1.4160596492\C,2.0837492659,-0.115333575,0.0473263408\C,0.8320093282,-0.2811387859,-0.5330544704\C,-1.4805217656,0.0268468702,2.418878488\N,-2.6334566437,-0.1020926142,1.7684894327\N,-3.7201234314,0.060114408,2.4315328821\S,-5.0896097496,-0.0320616643,1.4993075081\O,-6.0193269882,-0.8651431785,2.218944171\C,-4.6446739952,-0.8163968849,-0.01004644\C,-4.2367010037,-0.0489674698,-1.091278438\C,-3.8213916252,-0.678675174,-2.2514538185\C,-3.8047765758,-2.0692132944,-2.3487139349\C,-4.2190016627,-2.8176954194,-1.2481817648\C,-4.6353990818,-2.2016850746,-0.0806786662\C,-3.376590186,-2.7421347556,-3.614581926\O,-5.5094308065,1.3192971305,1.2005805008\C,-1.5023355624,0.188180751,3.8335263319\O,-2.1193714978,1.6712539861,4.3751688246\C,-2.3594637074,2.6731805647,3.714845698\C,-3.1876691202,3.8532639038,4.1568334797\C,-2.9367772817,4.421677597,5.5390786841\C,-1.8672185071,3.3511036507,2.4331113767\C,-2.3732719148,2.9170272141,1.0632039928\C,-2.5954798199,4.58977259,2.9659877146\S,-2.6672107465,6.1042405853,2.4630828235\C,-0.3475016636,3.5247366527,2.440728\C,-4.675288466,3.5360746048,3.922766559\H,-1.2869941197,-0.366672565,-0.2230368552\H,0.7474362181,-0.444243378,-1.6019249016\H,2.9811773886,-0.1471732734,-0.5590099801\H,3.1321033337,0.2219204662,1.8897070702\H,1.1254014904,0.3006571318,3.2580804782\H,-2.2896578122,-0.3667958563,4.3353790744\H,-0.5640949632,0.1891104087,4.3728399477\H,-3.499022593,-0.0808752606,-3.0971896671\H,-4.2450450379,1.031103801,-

1.0182613274\H,-4.9515774927,-2.7880662469,0.7731617354\H,-4.211007946,-3.90067556,-1.3065377187\H,-4.2318339719,-2.8800180223,-4.2832676307\H,-2.6364138806,-2.1455619188,-4.1499239487\H,-2.9537928556,-3.7280589716, -3.4164612367\H,-5.2312528103,4.473158939,3.9767600642\H,-4.8600071492, 3.0680432813,2.9550557485\H,-5.0214903751,2.8593862294,4.7051693342\H,-3.4914196072,5.3552593949,5.6442094519\H,-3.2846155782,3.7213713319, 6.2996603367\H,-1.8785039615,4.6274172419,5.7043704626\H,-0.0986880932,4.3026501027,1.7168081618\H,0.031618421,3.8308025984, 3.41717501\H,0.1358434978,2.5937768693,2.1451726214\H,-2.225026711, 3.7631348248,0.3889618552\H,-1.8046985851,2.0659733604,0.6934798237\H,-3.4312493576,2.659109 4997,1.0772297377\\Version=ES64L-G16RevB.01\HF=-2022.3688762\RMSD=3.754e-09\RMSF=1.560e-06\Dipole=3.1721972, 0.7674747,0.5960845\Quadrupole=-41.008038,7.162435,33.8456029,0.1041996, 12.2149359,18.6685396\PG=C01[X(C23H26N2O3S2)]\\@

1i+7a (NNC) Addition to C=O bond

HF=-2022.4348601 a.u. (0)

1\1\GINC-R09N18\FOpt\RPBE1PBE\def2TZVP\C23H26N2O3S2\WURTHWE\15-Feb-2020\0\\# pbe1pbe/def2tzvp Opt=(maxstep=8,readfc) geom=check guess=read Pop=NBO Freq emp=gd3bj nosym scrf=(solvent= dichloromethane) 1i+7a (NNC) C=O addition\\0,1\C,0.0240293644,-0.0546454234,0.1430489388\C,0.118977292,0.0681915276,1.5294045243\C,1.3662580742,0.3097544606,2.1078509769\C,2.4939891244,0.4198656507,1.3150179601\C,2.3930674881,0.2894078099,-0.0648155765\C,1.157251095, 0.0507003886,-0.6476694939\C,-1.0709541227,-0.0743758153,2.3803671912\C,-2.4185104161,0.0636644431,1.735389312\O,-3.4967883499,0.1886867833, 2.6385118197\C,-3.0927979851,0.4850999548,3.9484930475\N,-2.0775220134,-0.448070796,4.3832602867\N,-0.8870274771,-0.3529022011,3.6175111789\S,-2.3869021786,-2.0812767819,4.6592242854\O,-1.2436448475,-2.5693034209, 5.3671982892\C,-4.3584355793,0.7726702734,4.8361544484\C,-5.6944883224, 0.3250862806,4.277914628\C,-2.6560198629,2.0040611601,4.1224386619\C,-2.3710843288,2.8335200768,2.8825088225\O,-3.6906881579,-2.1767076228, 5.2409856544\C,-4.270993088,0.4313075337,6.3289631521\C,-4.038757952, 2.240997975,4.681607301\S,-4.8835434216,3.5776068171,4.966176258\C,-1.567669299,2.2496028494,5.1637848907\C,-2.4331785476,-2.8511488218 ,3.0825650199\C,-1.2488403998,-3.2270331027,2.4612409129\C,-1.2958444959,-3.7607771756,1.1861865073\C,-2.5100936561,-3.9353089075,0.5232097509\C,-3.685862666,-3.5658283341,1.1764177085\C,-3.6564808301,-3.0226548818, 2.4481585951\C,-2.5515068141,-4.5314259708,-0.8473923853\H,1.4333076319, 0.4142840677,3.1831824509\H,3.4567368237,0.6133105664,1.7731607775\H,3.2775219061,0.3775130205,-0.6848393052\H,1.0731750875,-0.0547116896,-

1.7226959517\H,-0.9304570301,-0.2526434113,-0.3290104639\H,-2.4091054177,
0.9078957805,1.0411968888\H,-2.5920739925,-0.8429001962,1.1442386772\H,-
4.638787999,-3.69946924,0.6766424982\H,-4.5695498289,-2.7229571331,
2.9453082431\H,-0.3039508799,-3.0875215499,2.9701513961\H,-0.3720180293,-
4.0418684943,0.6930593335\H,-2.612327823,-5.6222657459,-0.7854782379\H,-
3.4227676744,-4.1857874258,-1.4050824829\H,-1.6516025527,-4.2864000738,-
1.4132229741\H,-1.5481987695,3.3136700384,5.4064658264\H,-1.7237690217,
1.6882272307,6.0820536903\H,-0.59739725,1.9647217477,4.7530891896\H,-
2.4096969856,3.8868033873,3.1671562947\H,-1.3756096276,2.6303218418,
2.4833572093\H,-3.1186402297,2.6748558949,2.1057495395\H,-6.492036255,
0.7791903563,4.869002363\H,-5.8193967582,0.6220880784,3.2379576454\H,-
5.7804807333,-0.7596735173,4.3529940972\H,-4.9217168228,1.1243717817,
6.8656925469\H,-4.6098211739,-0.5857020301,6.5020181391\H,-3.2628709178,
0.527192421,6.7278380275\\Version=ES64L-G16RevB.01\HF=-2022.4348601\
RMSD=6.425e-09\RMSF=5.811e-07\Dipole=1.1140665,-0.476779,-3.9719059\
Quadrupole=12.1853576,9.9589874,-22.1443451,11.8348811,20.2091236,
11.8607859\PG=C01[X(C23H26N2O3S2)]\\@

TS for 1i+7a (NNO) Addition to C=O bond
HF=-2022.3565188 a.u. (1, -429.2590 cm^{-1})
1\1\GINC-R09N13\FTS\RPBE1PBE\def2TZVP\C23H26N2O3S2\
WURTHWE\10-Mar-2020\0\\# pbe1pbe/def2tzvp Opt=(ts,noeigentest,
readfc) geom=check guess=read scrf=(solvent=dichloro methane) freq pop=nbo
emp=gd3bj nosym\\TS for 1i+7a (NNO)-C=O-addition\\0,1\C,-0.2450818418,
0.488521147,0.2944945644\C,-0.2956089825,0.2544557146,1.6721042834\
C,0.8951802821,0.2263939662,2.4014557929\C,2.107587385,0.4402676683,
1.7704378115\C,2.148114487,0.6814662446,0.4032821511\C,0.9696544364
,0.7015572492,-0.3317890287\C,-1.5733094329,0.0281962112,2.3386915411\C,-
1.858539831,0.3232380369,3.6665554228\C,-3.2102707354,1.7133870738,
3.6780667982\O,-4.1624999849,1.2617713536,2.9987419059\N,-3.6671980283,-
0.655789119,2.0948976323\N,-2.6045077081,-0.3371766108,1.5257216452\S,-
4.9780197107,-0.8204484637,0.9543564867\O,-4.726622602,-2.08536266,
0.3229846042\C,-3.3881822398,2.0952763788,5.2081685357\C,-2.7010535383,
3.3977757598,4.8625296812\S,-2.2461837151,4.6465330277,5.7699301151\C,-
2.5477584994,3.1171421974,3.3818115186\C,-1.1213521406,3.2387067569,
2.8846324987\C,-4.7706402672,0.4728325269,-0.1972029737\C,-5.3389285406,
1.7120291104,0.0656221912\C,-5.1313155816,2.7455239409,-0.8291530082\C,-
4.3540696499,2.5642848424,-1.9727817332\C,-3.7987591655,1.3075325233,-
2.2125600043\C,-3.9994981406,0.2597281317,-1.3328799279\C,-4.1018917087,
3.7011527814,-2.9106444642\O,-6.1658470736,-0.6287543517,1.7256522773\C,-
4.8698924431,2.28926716,5.5210426076\C,-2.7312882346,1.3097952546,
6.3268878065\C,-3.466372388,3.9765440112,2.5192773459\H,-1.1687032579,

0.5251103947,-0.26884541\H,0.9981764753,0.8931193499,-1.3979132319\
H,3.0984255348,0.8504029356,-0.089352876\H,3.0256063437,0.409968862,
2.3449250679\H,0.8742635352,0.0116292495,3.4632424755\H,-2.584626994,-
0.3247586279,4.1471700188\H,-1.0528252889,0.6940297959,4.2826974167\H,-
5.5787619147,3.7142512556,-0.636527041\H,-5.9279264646,1.8566810078,
0.9610429269\H,-3.5724803598,-0.7166271766,-1.5212146697\H,-3.2030717087,
1.1480399459,-3.104416704\H,-3.2021381606,4.247107467,-2.6102159802\H,-
3.9450570563,3.3480903743,-3.9306300213\H,-4.9306480784,4.4102308906,-
2.9072566783\H,-2.9314668293,1.810623151,7.2764183402\H,-3.1527611109,
0.3032767494,6.3814529739\H,-1.6497702271,1.2361247074,6.2187510017\H,-
4.9750891745,2.7934527969,6.4834856914\H,-5.3768649865,2.8782143754,
4.7570838456\H,-5.3580755029,1.3145131452,5.5740065111\H,-0.8196110345,
4.2874447127,2.9351340631\H,-0.4146321689,2.662225074,3.480653441\H,-
1.0522245231,2.9116181957,1.8462768149\H,-3.118108866,5.0111520744,
2.5362499495\H,-3.4433088989,3.6097855966,1.4915107518\H,-4.500060528,
3.9474152343,2.863634811\\Version=ES64L-G16RevB.01\HF=-2022.3565188
\RMSD=5.076e-09\RMSF=1.936e-06\Dipole=2.8835817,1.5579383,-1.3127246\
Quadrupole=-16.5669721,1.2203163,15.3466558,-18.0215084,15.573721,-
16.1694893\PG=C01[X(C23H26N2O3S2)]\\@

1i+7a (NNO) Addition to C=O bond
HF=-2022.4016417 a.u. (0)
1\1\GINC-R10N25\FOpt\RPBE1PBE\def2TZVP\C23H26N2O3S2\
WURTHWE\15-Feb-2020\0\\# pbe1pbe/def2tzvp Opt=(maxstep=8,readfc)
geom=check guess=read Pop=NBO Freq emp=gd3bj nosym scrf=(solvent=
dichloromethane)\\ 1i+7a (NNO) C=O-addition\\0,1\C,0.0313834557,
0.2962213103,-0.0383348507\C,-0.0423329156,-0.2452671108,1.2444477936\
C,1.0540727346,-0.9502587473,1.7470967699\C,2.1989505418,-1.0994289557,
0.9862379683\C,2.2667728448,-0.5501472123,-0.2887347664\C,1.1816263003,
0.1480989125,-0.7973314564\C,-1.2447940042,-0.0839696527,2.0669801727\C,-
2.5826436282,0.2655933689,1.5115590931\C,-3.6462535201,-0.6714882297,
2.0653813998\O,-3.327603418,-0.9835451359,3.4063557984\N,-2.3620648435,-
0.0919729976,3.9663366264\N,-1.1440238886,-0.2170654728,3.3400328183\S,-
2.2410652317,-0.5329975449,5.6199551594\O,-1.1971362581,0.3020142092,
6.1249000766\C,-5.1351299465,-0.2280887091,1.8245778051\C,-5.3759897003,
1.1174892009,1.1633367191\C,-3.9151703002,-1.9877108788,1.2495723585\C,-
3.0136792441,-2.2889255145,0.0648309286\C,-1.7297634061,-2.1994772388,
5.6057314177\C,-0.3751034869,-2.494862912,5.5093648082\C,0.0166604452,-
3.8180345383,5.4210611203\C,-0.9219900969,-4.8496347632,5.4261258654\C,-
2.2742126206,-4.5232196712,5.5226132365\C,-2.6862280978,-3.2062863116,
5.609414885\C,-0.4855272335,-6.2781377669,5.3569479672\O,-3.572436403,-
0.4309393072,6.1288653473\C,-5.2695595922,-1.4142672799,0.8942960159\S,-

6.4075498817,-1.867897503,-0.1469729721\C,-6.0153324008,-0.3673117243,
3.0621474648\C,-4.0434858001,-3.2368502346,2.1195628019\H,0.9860779407,-
1.38889245,2.7348316044\H,3.0408103133,-1.6541966927,1.3836937146\
H,3.16271523,-0.6719394373,-0.8861533053\H,1.2291043733,0.5804934239,-
1.7896992553\H,-0.8026715068,0.854360593,-0.445742624\H,-2.8139319676,
1.2742144974,1.8740200572\H,-2.6054516051,0.2870652468,0.4275583868\H,-
3.0157600741,-5.3143073383,5.5246651988\H,-3.7360321169,-2.9533264128,
5.6779025297\H,0.35330562,-1.6949302804,5.5061529307\H,1.0716765214,-
4.0568667138,5.3457610198\H,-0.3205719782,-6.6739731194,6.3637632051\H,-
1.2428858107,-6.9018616565,4.8803589697\H,0.4511396843,-6.3809786583,
4.8075382528\H,-6.4255658238,1.1902809945,0.8720889526\H,-5.1577836697,
1.929869473,1.8602761962\H,-4.7720545189,1.2539797401,0.2652095596\H,-
7.0609541579,-0.2543649842,2.7702655681\H,-5.8945374308,-1.3302459972,
3.5557479574\H,-5.7604791635,0.4131485532,3.7814436877\H,-3.393437385,-
3.1666987041,-0.4609160593\H,-2.9741894305,-1.4691959773,-0.653035192\H,-
1.9988015436,-2.5066795888,0.4059733657\H,-4.4617278308,-4.0430329387,
1.5143742361\H,-3.0586684487,-3.5363113495,2.4817384512\H,-4.6868766309,-
3.0842966682,2.9845304238\\Version=ES64L-G16RevB.01\HF=-2022.4016417\
RMSD=4.146e-09\RMSF=7.846e-07\Dipole=1.6579838,-1.7724789,-1.8736575\
Quadrupole=-5.7278046,28.4256563,-22.6978518,-5.5570527,7.2912398,-
26.0708373\PG=C01[X(C23H26N2O3S2)]\\@

4. References

[1] Gaussian 16, Revision B.01, Frisch, M. J.; Trucks, G. W.; Schlegel, H. B.; Scuseria, G. E.; Robb, M. A.; Cheeseman, J. R.; Scalmani, G.; Barone, V.; Petersson, G. A.; Nakatsuji, H.; Li, X.; Caricato, M.; Marenich, A. V.; Bloino, J.; Janesko, B.G.; Gomperts, R.; Mennucci, B.; Hratchian, H. P.; Ortiz, J. V.; Izmaylov, A. F.; Sonnenberg, J. L.; Williams-Young, D.; Ding, F.; Lipparini, F.; Egidi, F.; Goings, J.; Peng, B.; Petrone, A.; Henderson, T.; Ranasinghe, D.; Zakrzewski, V. G.; Gao, J.; Rega, N.; Zheng, G.; Liang, W.; Hada, M.; Ehara, M.; Toyota, K.; Fukuda, R.; Hasegawa, J.; Ishida, M.; Nakajima, T.; Honda, Y.; Kitao, O.; Nakai, H.; Vreven, T.; Throssell, V; Montgomery, Jr., J. A.; Peralta, J. E.; Ogliaro, F.; Bearpark, M. J.; Heyd, J. J.; Brothers, E. N.; Kudin, K. N.; Staroverov, V. N.; Keith, T. A.; Kobayashi, R.; Normand, J.; Raghavachari, K.; Rendell, A. P.; Burant, J. C.; Iyengar, S. S.; Tomasi, J.; Cossi, V; Millam, J. M.; Klene, M.; Adamo, C.; Cammi, R.; Ochterski, J. W.; Martin, R. L.; Morokuma, K.; Farkas, O.; Foresman, J. B.; Fox, D. J. Gaussian, Inc., Wallingford CT, 2016.

[2] Becke, A. D. Density-functional thermochemistry. III. The role of exact exchange. *J. Chem. Phys.* **1993**, *98*, 5648–5652.

[3] Lee, C.; Yang, W.; Parr, R. G. Development of the Colle-Salvetti correlation-energy formula into a functional of the electron density. *Phys. Rev. B* **1988**, *37*, 785–789.
[4] Grimme, S.; Ehrlich, S.; Goerigk, L. Effect of the damping function in dispersion corrected density functional theory. *J. Comput. Chem.* **2011**, *32*, 1456–1465.
[5] Grimme, S.; Hansen, A.; Brandenburg, J. G.; Bannwarth, C. Dispersion-corrected mean-field electronic structure methods. *Chem. Rev.* **2016**, *116*, 5105–5154.
[6] Perdew, J. P.; Burke, K.; Ernzerhof, M. Generalized gradient approximation made simple. *Phys. Rev. Lett.* **1996**, *77*, 3865–3868.
[7] Perdew, J. P.; Burke, K.; Ernzerhof, M. Errata: Generalized gradient approximation made simple. *Phys. Rev. Lett.* **1997**, *78*, 1396.
[8] Adamo, C.; Barone, V. Toward reliable density functional methods without adjustable parameters: The PBE0 model. *J. Chem. Phys.* **1999**, *110*, 6158–6169.
[9] Ernzerhof, M.; Scuseria, G. E. Assessment of the Perdew-Burke-Ernzerhof exchange-correlation functional. *J. Chem. Phys.* **1999**, *110*, 5029–5036.
[10] Weigend, R.; Ahlrichs, R. Balanced basis sets of split valence, triple zeta valence and quadruple zeta valence quality for H to Rn: Design and assessment of accuracy. *Phys. Chem. Chem. Phys.* **2005**, *7*, 3297–3305.
[11] Tomasi, J.; Mennucci, B.; Cammi, R. Quantum Mechanical Continuum Solvation Models. *Chem. Rev.* **2005**, *105*, 2999–3093.
